# New Hybrid Hydrazinyl Thiazole Substituted Chromones: As Potential *α*-Amylase Inhibitors and Radical (DPPH & ABTS) Scavengers

**DOI:** 10.1038/s41598-017-17261-w

**Published:** 2017-12-05

**Authors:** Uzma Salar, Khalid Mohammed Khan, Sridevi Chigurupati, Muhammad Taha, Abdul Wadood, Shantini Vijayabalan, Mehreen Ghufran, Shahnaz Perveen

**Affiliations:** 1 H. E. J. Research Institute of Chemistry, International Center for Chemical and Biological Sciences, University of Karachi, Karachi, 75270 Pakistan; 2 Department of Pharmaceutical chemistry, Faculty of Pharmacy, AIMST University, Semeling, 08100 Bedong, Kedah Malaysia; 3 Department of Clinical Pharmacy, Institute for Research and Medical Consultations (IRMC), Imam Abdulrahman Bin Faisal University, Dammam, P.O. Box 31441 Saudi Arabia; 4 Department of Biochemistry, Computational Medicinal Chemistry Laboratory, UCSS, Abdul Wali Khan University, Mardan, Pakistan; 5 PCSIR Laboratories Complex, Karachi, Shahrah-e-Dr. SalimuzzamanSiddiqui, Karachi, 75280 Pakistan

## Abstract

Current research is based on the identification of novel inhibitors of *α*-amylase enzyme. For that purpose, new hybrid molecules of hydrazinyl thiazole substituted chromones **5**–**27** were synthesized by multi-step reaction and fully characterized by various spectroscopic techniques such as EI-MS, HREI-MS, ^1^H-NMR and ^13^C-NMR. Stereochemistry of the iminic bond was confirmed by NOESY analysis of a representative molecule. All compounds **5–27** along with their intervening intermediates **1–4**, were screened for *in vitro α*-amylase inhibitory, DPPH and ABTS radical scavenging activities. All compounds showed good inhibition potential in the range of IC_50_ = 2.186–3.405 *µ*M as compared to standard acarbose having IC_50_ value of 1.9 ± 0.07 *µ*M. It is worth mentioning that compounds were also demonstrated good DPPH (IC_50_ = 0.09–2.233 *µ*M) and ABTS (IC_50_ = 0.584–3.738 *µ*M) radical scavenging activities as compared to standard ascorbic acid having IC_50_ = 0.33 ± 0.18 *µ*M for DPPH and IC_50_ = 0.53 ± 0.3 *µ*M for ABTS radical scavenging activities. In addition to that cytotoxicity of the compounds were checked on NIH-3T3 mouse fibroblast cell line and found to be non-toxic. *In silico* studies were performed to rationalize the binding mode of compounds (ligands) with the active site of *α*-amylase enzyme.

## Introduction

Diabetes mellitus (DM) is a metabolic disorder caused by the insufficient insulin secretion and decreased insulin activity which leads to the disruption of carbohydrate, protein, and fat metabolism^1^. Insulin is a peptide hormone which is responsible to reduce gluconeogenesis, increases the glucose consumption, and drops the blood glucose level^2^. However, failure in insulin secretion or disturbance in insulin sensitivity give rise to uncontrolled blood glucose levels (hyperglycemia) and ultimately results in DM. In addition to that continuous complications of DM further brings out to neuropathy, nephropathy, retinopathy, microangiopathy as well as cardiovascular diseases^1^.

Treatment of type-II DM includes a number of therapeutic approaches such as stimulation of the endogenous insulin secretion, reduction of insulin’s demand, and inhibition of carbohydrate degradation^3^. One of therapeutic strategies is to reduce the post-prandial glucose levels by retarding the absorption of glucose. This could possibly be done by the inhibition of enzymes, *α*-glucosidase and *α*-amylase, those are responsible to hydrolyze oligosaccharides and disaccharides into monosaccharides^1,4–6^. The *α*-amylase (*α*-1,4-glucan-4-glucanohydrolases; E.C. 3.2.1.1) is one of the main enzyme secreted by the pancreas (about 5–6%) and salivary glands, and shows a significant role in digestion or breakdown of starch and glycogen and usually found in microbes, plants, and higher organisms^7,8^. Inhibitors of *α*-amylase enzyme such as acarbose, function by delaying the carbohydrate digestion and cause a decreased rate of glucose absorption and accordingly diminishing the postprandial plasma glucose level^9,10^. However, adverse effects are also associated such as abdominal discomfort, meteorism, flatulence, and diarrhea which lead to discontinuation of therapy^1^. Some natural products such as flavonoids and phenolic compounds has been identified as *α*-amylase inhibitors^1,11–13^. However, the synthetic inhibitors are rarely discovered. There is an urgent need for the discovery of novel therapeutic agents for the management of type-II diabetes mellitus.

Chromone or 4*H*-chromen-4-one is a naturally occurring heterocycle based on benzopyrone scaffold and widely distributed in nature mainly in plants. It is also the core fragment of several flavonoids *e.g*. flavones and isoflavones. Chromone derived compounds have a wide-range of biological activities such as antioxidant, antihypertensive, antiinflammatory, anticancer, antifungal, antibacterial, antiviral, antimutagenic, and phytotoxic activities^14^. Chromones have also been reported to possess lipoxygenase, thymidine phosphorylase, cyclooxygenase, tyrosine and protein kinase inhibitory activities^15–19^. Similarly, heterocyclic ring thiazole has also reported to be the main part of countless medicinally important molecules due to its notable biological activities^20,21^.

**Figure 1:**
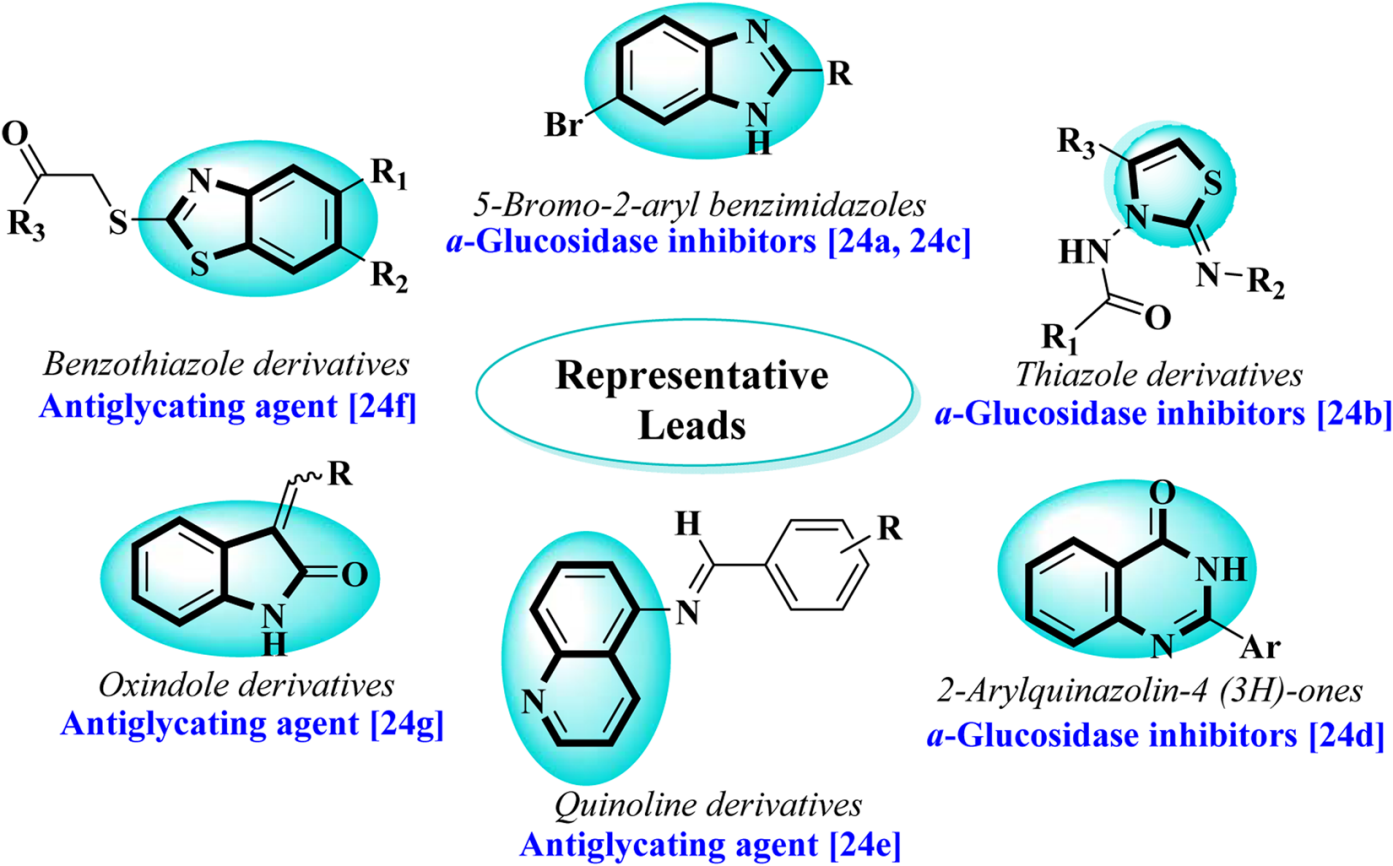
Identified representative lead candidates.

A number of reports available on hybrid scaffolds based on thiazole linked with chromone scaffold with some biological potentials^22–25^. Nevertheless, there is no report available on this hybrid class for their *α*-amylase inhibitory activity. Our research group has identified many lead scaffolds having antiglycation and *α*-glucosidase inhibitory activities (Fig. 1), as a possible treatment for diabetic management^26–31^.

**Figure 2:**
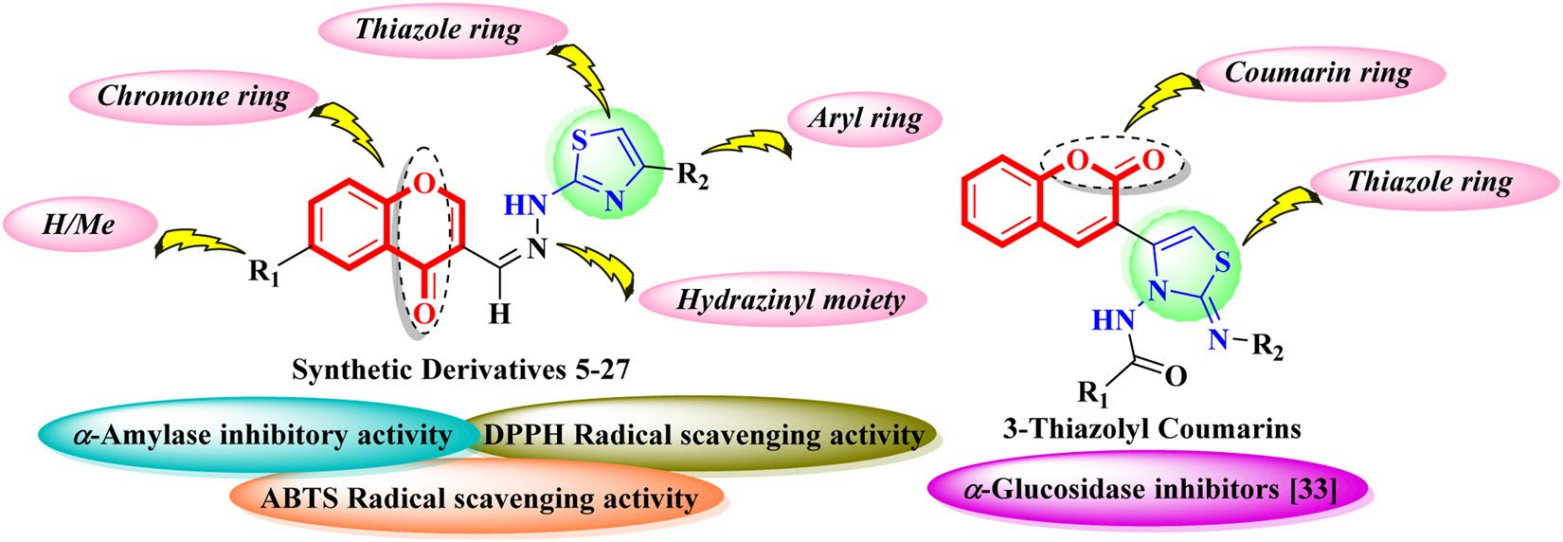
Rationale of the current study.

We have also reported 3-thiazolyl coumarin as potent inhibitors of *α*-glucosidase enzyme^32^. It is worth-mentioning that newly synthesized compounds have close structural resemblance with the 3-thiazolyl coumarins (Fig. 2), so that we decided to explore the new hybrid hydrazinyl thiazole substituted chromones **5**–**27** along with the intervening intermediates for *α*-amylase inhibitory activity in order to identify novel inhibitors. Furthermore, by keeping in mind that excess free radical formation is also associated with the diabetic patients, so that synthetic analogs were also evaluated for their radical scavenging activities (DPPH and ABTS). To the best of our knowledge, except compounds **1–5** ^33–36^ all compounds are new.

## Results and Discussion

### Chemistry

**Figure 3:**
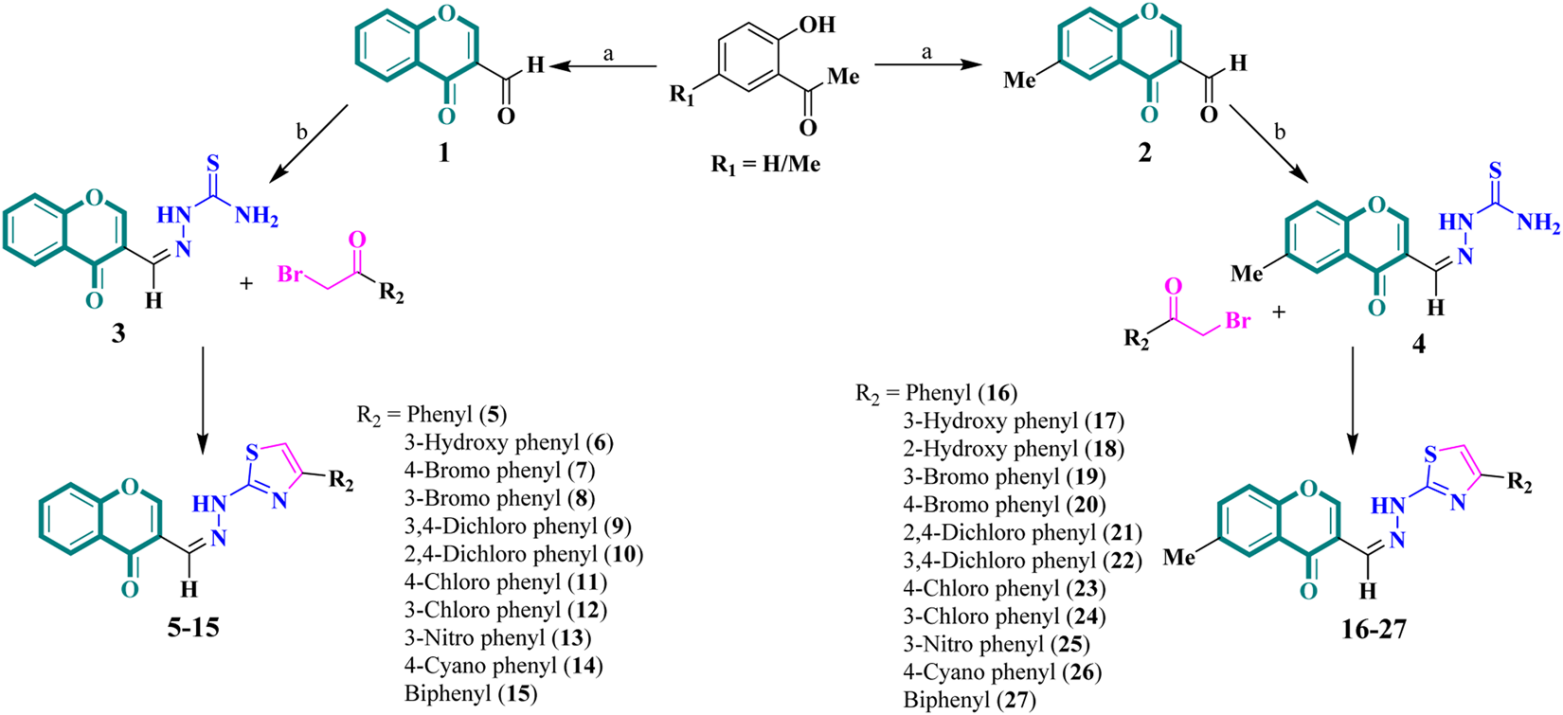
Syntheses of hydrazinyl thiazole substituted chromone derivatives **5–27** *via* intervening intermediates **1–4**; **Reaction conditions**: (a) POCl_3_, DMF; (b) Thiosemicarbazide, AcOH, EtOH, Reflux, 2 h; (c) Et_3_N, EtOH, Reflux, 3 h.

New hybrid hydrazinyl thiazole substituted chromones **5–27** were synthesized by multi-step reaction. First, chromone-3-carbaldehyde **1** and 6-methylchromone-3-carbaldehyde **2** were synthesized by reacting 2-hydroxy acetophenone and 5-methyl-2-hydroxy acetophenone with the dimethyl formamide (DMF) in the presence of phosphoryl chloride (POCl_3_)^14^. In the next step, chromone-3-carbaldehyde derivatives (**1** and **2**) were condensed with thiosemicarbazide in ethanol to afford their corresponding thiosemicarbazone derivatives (**3** and **4**), in the presence of glacial acetic acid. These thiosemicarbazone derivatives (**3** and **4**) were reacted with different phenacyl bromides which underwent a cyclization reaction in the presence of triethylamine^32^ resulting in the formation of desired products (Fig. 3). Reaction progress was checked by periodic thin layer chromatography (TLC). Chemical structures of compounds **1–27** were elucidated by using spectroscopic techniques such as EI-MS, HREI-MS, ^1^H-NMR and ^13^C-NMR.

**Figure 4:**
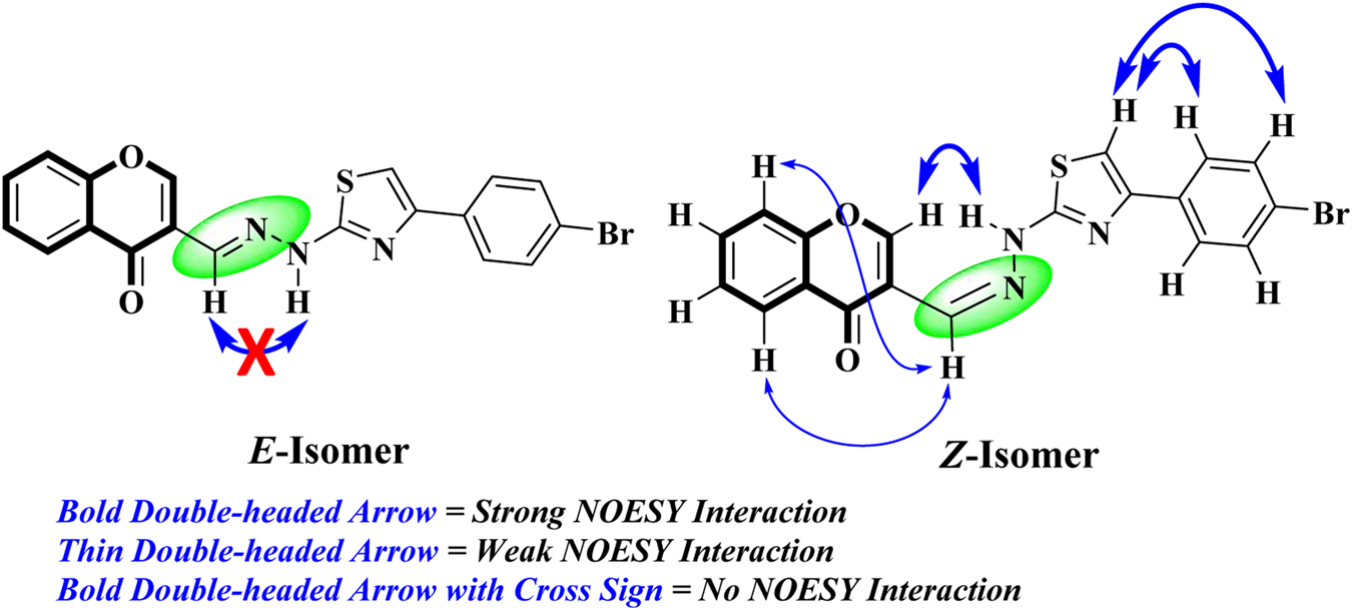
Distinctive NOESY interactions.

To confirm the stereochemical assignment of iminic double bond, NOESY (nuclear overhauser enhancement spectroscopy) was performed on a representative derivative **7**. Many interactions were observed in the NOESY spectrum, some of them confirmed the (*Z*) stereochemistry of the iminic double bond. Strong NOESY interaction between the NH proton and CH-2 of chromone ring was observed which can only be observed in case of *Z*-isomer. Similarly, absence of NOESY interaction between the NH and H-C = N protons further confirms the *Z*-stereochemistry of resulting isomer (Fig. 4). Other interactions such as strong interactions of H-5ʹ with H-2ʹʹ/H-6ʹʹ and H-3ʹʹ/H-5ʹʹ as well as weak interactions of iminic proton with H-5 and H-8 were also observed.

### Mass Spectrometry

Low resolution EI-MS of compound **7** displayed the molecular ion peak [M]^+^ at *m/z* 425 and [M + 2]^+^ at *m/z* 427 which confirmed the presence of bromine substitution. High resolution EI-MS displayed [M]^+^ at *m/z* = 424.9806 with a composition of C_19_H_12_BrN_3_O_2_S (Calcd. 424.9834) which also confirmed the formation of desired compound. Low resolution EI-MS spectrum showed many characterisric fragments. A fragmentation pattern is discussed below.

### Structure-fragmnetation pattern

**Figure 5:**
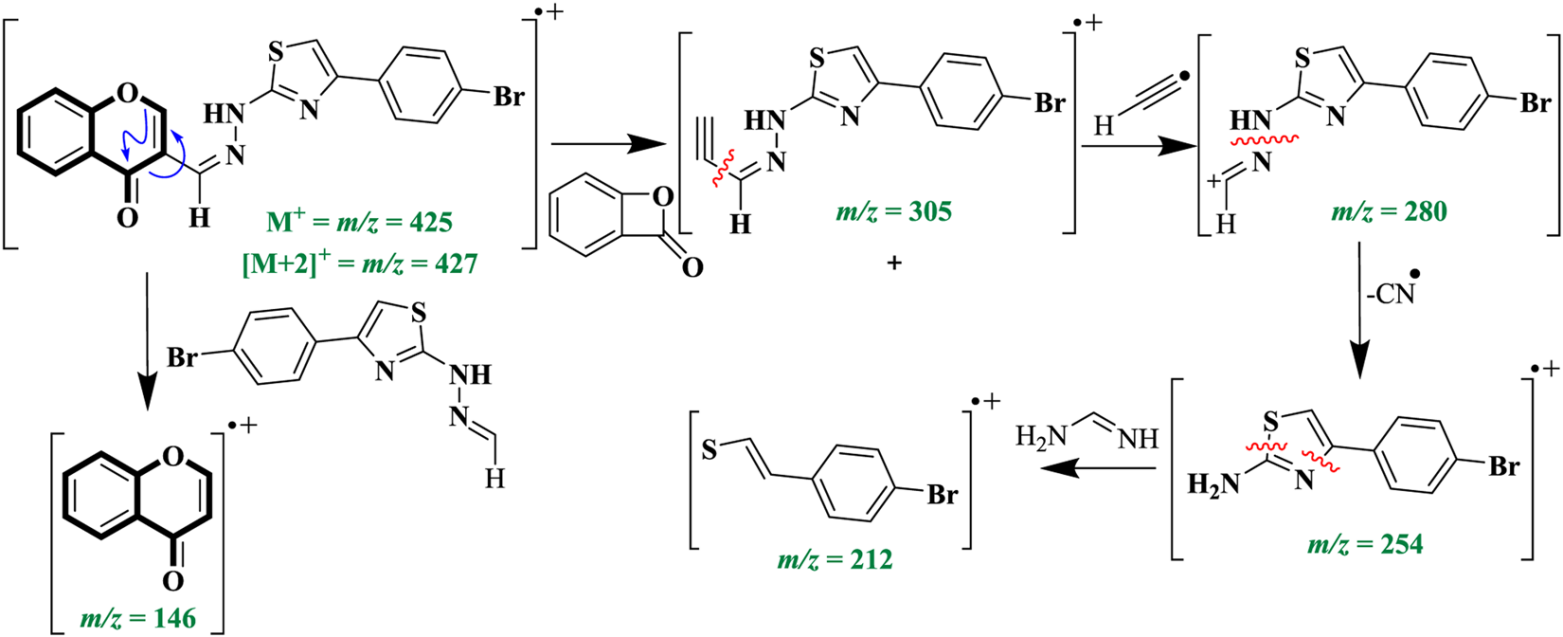
Key EI-MS fragmentation of compound **7**.

The molecular ion at *m/z* 425 was fragmented to afford a radical cation at *m/z* 305 by the neutral loss of 7-oxabicyclo[4.2.0]octa-1,3,5-trien-8-one molecule. The resulting radical cation further cleaved to give a cation at *m/z* 280 by the loss of hydrogen cyanide radical. Cation obtained at *m/z* 280 undergo two successive cleavage to afford radical cations at *m/z* 254 and *m/z* 212 with the losses of nitrile radical and neutral formimidamide molecule, respectively. Similarly, molecular ion at *m/z* 425 also fragmented in another fasion to give a chromone radical cation at *m/z* 146 by the neutral loss of rest of the molecule (Fig. 5).

### *In vitro* biological activities

All hybrid hydrazinyl thiazole substituted chromones **5**–**27** along with the intervening intermediates **1**–**4** were evaluated to check their *α*-amylase inhibitory^37–39^, DPPH^40–42^ and ABTS^43^ radical scavenging, and cytotoxic^44^ activities. Results depicted in Table 1 showed that all compounds displayed comparable *α*-amylase inhibitory activities in the range of IC_50_ = 2.186 ± 0.03–3.405 ± 0.21 *µ*M as compared to standard acarbose IC_50_ = 1.9 ± 0.07 *µ*M. All analogs were also showed good DPPH and ABTS radical scavenging activities in the ranges of IC_50_ = 0.09 ± 0.17–2.233 ± 0.6 *µ*M and IC_50_ = 0.584 ± 0.07–3.738 ± 0.6 *µ*M, respectively, as compared to standard ascorbic acid (IC_50_ = 0.33 ± 0.18 *µ*M and IC_50_ = 0.53 ± 0.3 *µ*M, respectively). It is worth-mentioning that all compounds were found to be non-toxic when tested on NIH-3T3 mouse fibroblast cell line by using the standard MTT colorimetric assay^44^.

**Table 1:** *α*-Amylase inhibitory, DPPH, and ABTS radical scavenging activities of hydrazinyl thiazole substituted chromones **5**–**27**, and intervening intermediates **1**–**4**.

Compounds	Structures	*α*-Amylase inhibitory activity	DPPH radical scavenging activity	ABTS radical scavenging activity
		IC_50_ ± SEM^a^	IC_50_ ± SEM^a^	IC_50_ ± SEM^a^
**1**		3.405 ± 0.21	2.23 ± 0.79	3.738 ± 0.6
**2**		3.400 ± 0.08	2.233 ± 0.6	2.23 ± 0.06
**3**		3.401 ± 0.18	1.905 ± 0.06	1.916 ± 0.09
**4**		2.842 ± 0.08	1.145 ± 0.12	1.258 ± 0.1
**5**		2.826 ± 0.06	1.113 ± 0.15	1.083 ± 0.15
**6**		3.382 ± 0.23	1.895 ± 0.03	1.892 ± 0.01
**7**		2.706 ± 0.05	0.892 ± 0.09	0.83 ± 0.25
**8**		2.707 ± 0.05	0.912 ± 0.23	0.914 ± 0.19
**9**		2.64 ± 0.05	0.314 ± 0.12	0.784 ± 0.12
**10**		2.335 ± 0.08	0.09 ± 0.17	0.677 ± 0.26
**11**		2.741 ± 0.1	0.922 ± 0.22	0.918 ± 0.14
**12**		2.767 ± 0.07	1.050 ± 0.03	1.083 ± 0.08
**13**		3.197 ± 0.13	1.745 ± 0.04	1.892 ± 0.05
**14**		2.877 ± 0.05	1.294 ± 0.1	1.261 ± 0.07
**15**		3.131 ± 0.28	1.406 ± 0.05	1.643 ± 0.04
**16**		2.99 ± 0.05	1.368 ± 0.2	1.319 ± 0.2
**17**		3.024 ± 0.04	1.336 ± 0.09	1.384 ± 0.05
**18**		3.065 ± 0.02	1.659 ± 0.6	1.734 ± 0.05
**19**		2.186 ± 0.03	0.781 ± 0.18	0.584 ± 0.07
**20**		2.335 ± 0.08	0.611 ± 0.29	0.66 ± 0.16
**21**		2.357 ± 0.1	0.684 ± 0.13	0.71 ± 0.09
**22**		2.428 ± 0.05	0.688 ± 0.17	0.719 ± 0.1
**23**		2.669 ± 0.06	0.84 ± 0.1	0.82 ± 0.18
**24**		2.749 ± 0.15	0.99 ± 0.12	1.02 ± 0.04
**25**		2.97 ± 0.09	1.307 ± 0.06	1.303 ± 0.11
**26**		2.887 ± 0.08	1.295 ± 0.13	1.277 ± 0.04
**27**		2.937 ± 0.06	1.336 ± 0.07	1.29 ± 0.07
**Standards**	Acarbose^b^	1.9 ± 0.07		
	Ascorbic acid^c^		0.33 ± 0.18	0.53 ± 0.3

IC_50_^a^ (Mean ± Standard deviation); Acarbose^b^ (Standard Inhibitor for *α*-amylase inhibitory activity); Ascorbic acid^c^ (Standard for DPPH and ABTS radical scavenging activity).

### Structure-activity relationship (SAR) for α-amylase inhibitory activity

**Figure 6:**
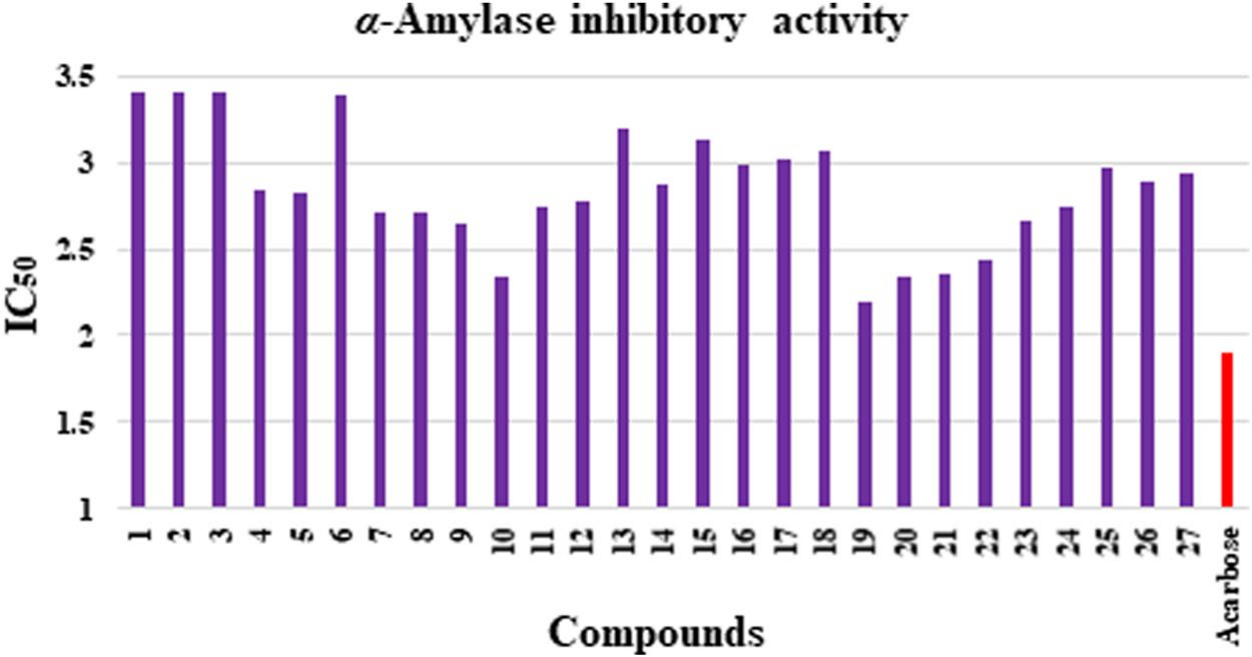
Comparison of *α*-amylase inhibitory activity of compounds.

Synthetic molecules possess very unique structural features (Fig. 2) and these features or pharmacophores are cordially playing their role in exhibiting *α*-amylase inhibition. However, the difference in the inhibitory activity is attributed by the varying features or groups present at aromatic rings *i.e*. R_1_ and R_2_. Figure 6 revealed that intervening intermediates **1** and **2** showed similar but two fold less *α*-amylase inhibition as compared to standard acarbose. However, thiosemicarbazone intermediate **4** with methyl substitution on chromone found to be better active than intermediate **3** which shows that the methyl substitution is influencing the binding interactions of compound with the active site of enzyme. The influence of methyl group is seemingly persists after the thiazole ring formation. Compound **5** with unsubstituted phenyl ring (R_2_) showed inhibitory activity comparable to standard. Incorporation of methyl group as R_1_ in compound **16** leads to slight decreased *α*-amylase inhibition potential. Compounds **6**, **17**, and **18**, having phenol as R_2_, showed decreased *α*-amylase inhibition as compared to the unsubstituted analogs **5** and **16**. Comparison of inhibitory activity of compound **6** with closely related compounds **17** and **18** revealed that incorporation of methyl group leads to increased activity. Amongst the halogens (Br and Cl) containing compounds, derivatives **7** and **8** with 4ʹʹ-bromo and 3ʹʹ-bromo phenyl group as R_2_, respectively, showed good and comparable *α*-amylase inhibition. However, analogs **20** and **19** with an additional methyl group as R_1_, showed increased activity. In case of mono-chlorinated derivatives, 6-methyl substituted compounds **23** and **24** having 4ʹʹ-chloro and 3ʹʹ-chloro substitutions on phenyl ring (R_2_), respectively, showed almost similar *α*-amylase inhibitory activity. Structurally similar analogs without methyl group as R_1_, *i.e*. **11** and **12** displayed slight decreased activities than **23** and **24**. Dichloro substituted derivatives **9**, **10**, **21**, and **22** were found to be more active than mono chloro substituted analogs which confirmed that chloro groups are actively participating in the activity. Amongst the 3ʹʹ-nitro substituted derivatives, compound **25** with methyl substitution as R_1_, demonstrated better *α*-amylase inhibitory activity as compared to compound **13**. In case of 4ʹʹ-cyano substituted anlogs **14** and **26**, both compounds showed almost similar activities which showed that presence of methyl group in compound **26** didn’t really make any difference in the activity. Compounds **15** and **27** having biphenyl ring as R_2_, also showed good activities. Amongst them compound **27** with methyl substitutions as R_2_, showed superior activity as compared to compound **15** which lacks the methyl group (Fig. 6).

### Structure-activity relationship (SAR) for DPPH and ABTS radical scavenging activities

**Figure 7:**
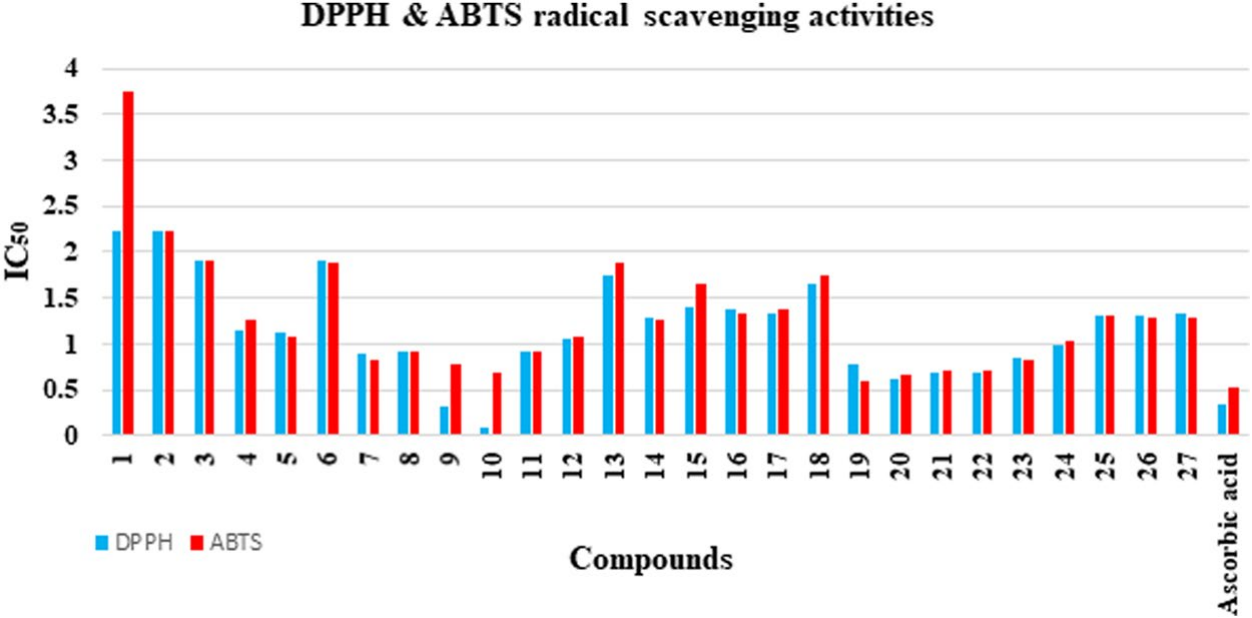
Comparison of DPPH & ABTS radical scavenging activities of compounds.

Variation in the DPPH and ABTS radical scavenging activities are resulted of varying structural features of compounds such as R_1_ and R_2_. Figure 7 depicts that the intervening intermediates **1** and **2** showed similar DPPH radical scavenging activities, however, compound **2** with methyl group as R_1_ showed better ABTS radical scavenging potential than compound **1**. Similarly, methyl bearing thiosemicarbazone intermediate **4** showed enhanced DPPH and ABTS radical scavenging activities as compared to non-methylated compound **3**. In case of thiazole ring containing compounds **5**–**27**, compound **5** with unsubstituted phenyl ring (R_2_) showed comparable DPPH and ABTS radical scavenging activities to standards. Incorporation of methyl group as R_1_ in compound **16** leads to slight decreased DPPH and ABTS radical scavenging activities. Phenol ring (R_2_) containing compounds **6**, **17**, and **18**, demonstrated diminished DPPH and ABTS radical scavenging activities as compared to the unsubstituted analogs **5** and **16**. In case of bromo substituted compounds, compound **7** and **8** with 4ʹʹ-bromo and 3ʹʹ-bromo phenyl group as R_2_, respectively, showed good and comparable DPPH and ABTS radical scavenging activities. However, their structurally similar analogs **19** and **20** with an additional methyl group as R_1_, showed enhanced activities. In case of mono-chlorinated derivatives, 4-chloro substituted derivative **23** showed better DPPH and ABTS radical scavenging activities as compared to 3-chloro substituted analog **24**. Nonetheless, their non-methylated structurally similar analogs *i.e*. **11** and **12** displayed slight decreased activities. Dichloro substituted analogs **9**, **10**, **21**, and **22** were showed superior activities than mono chloro substituted analogs. 4ʹʹ-Cyano substituted anlogs **14** and **26** showed almost similar activities. Furthermore, 3ʹʹ-nitro substituted derivative **25** with methyl substitution as R_1_, demonstrated better DPPH and ABTS radical scavenging activities as compared to compound **13**. Compounds **15** and **27** with biphenyl ring as R_2_, also showed good activities (Fig. 7).

Limited structure-activity relationship suggested that all compounds showed almost closed *α*-amylase inhibitory, DPPH, and ABTS radical scavenging activities. It indicates that all structural features including R_1_ and R_2_ are positively contributing in the activities. However, it was noticed that the halogen bearing molecules were found to have better activities than other groups such as OH, CN, NO_2_, and Ph. As well as most of the compounds having methyl group as R_1_ were found to be more active than the compounds without methyl substitution. In order to understand the binding interactions of compounds (ligands) with the active site of *α*-amylase enzyme, molecular modeling study was carried out.

### *In silico* studies

MOE-Dock module implemented in MOE program^45^ was utilized to explore the binding conformations of the compounds within the active site of *α*-amylase enzyme. The default parameters of MOE-Dock program were used in the docking protocol. At the end of docking experiment, the best conformations on the basis of docking score were analyzed for hydrogen bonding/arene-arene/arene-cation interactions. From the docking calculation study, it was observed that the top-ranked conformations of almost all compounds were well accommodated inside the active site of *α*-amylase enzyme and were involved in various type of interactions with the active site residues of *α*-amylase enzyme. *i.e*., Trp58, Trp59, Tyr62, Leu162, Arg195, Asp197, Glu233, Asp300, Asp356 etc. The detail of the docking scores and interactions for all compounds are collected in Table 2. Compound **19** exhibited good inhibitory potential with docking score of −9.7919 against *α*-amylase enzyme. Such lower values indicated good fitness of the compound in the binding pocket of the target enzyme and formation of a stable inhibitor protein complex. Compound **20** also showed good but slightly inferior inhibitory potential as compared to compound **19** with docking score of −8.9694 against *α*-amylase (Table 2).

**Table 2:** Docking scores and report of predicted interactions of docked conformations.

Compounds	Docking scores	Interactions Report				
		Ligand	Receptor	Interaction	Distance	E (kcal/mol)
**1**	−5.9012	C 13	OD1 ASP 197	H-donor	3.72	−0.6
		6-ring	CD2 LEU 162	*π*-H	4.23	−0.7
**2**	−5.1034	—				
**3**	−5.239	N 22	OD1 ASP 197	H-donor	3.26	−1.6
		S 25	NH2 ARG 195	H-acceptor	4	−0.9
		6-ring	5-ring TRP 59	*π*-*π*	3.81	−0.0
**4**	−7.0132	N 22	OD1 ASP 300	H-donor	3.15	−2.9
		N 25	OD2 ASP 300	H-donor	3.16	−2.3
**5**	−7.1717	6-ring	5-ring TRP 59	*π*-*π*	3.9	−0.0
		6-ring	6-ring TRP 59	*π*-*π*	3.93	−0.0
**6**	−6.13	O 38	OD2 ASP 300	H-donor	3.28	−1.6
**7**	−7.299	BR 38	OE1 GLU 233	H-donor	3.71	−1.9
		6-ring	CG PRO 54	*π*-H	4.08	−0.9
**8**	−7.2813	S 27	O TYR 62	H-donor	4.35	−0.8
		6-ring	6-ring TRP 59	*π*-*π*	3.9	−0.0
**9**	−7.9712	CL 37	OE1 GLU 233	H-donor	3.58	−1.4
		6-ring	N GLY 306	*π*-H	4.28	−0.6
		6-ring	N GLY 306	*π*-H	3.81	−0.7
**10**	−9.7828	N 20	6-ring TYR 62	H-*π*	4.68	−0.8
		6-ring	6-ring TRP 59	*π*-*π*	3.94	−0.0
**11**	−7.1399	S 27	OD1 ASP 197	H-donor	4.11	−0.7
		6-ring	CD1 LEU 162	*π*-H	4.5	−0.6
		6-ring	5-ring TRP 59	*π*-*π*	3.9	−0.0
**12**	−7.1292	S 27	OD2 ASP 356	H-donor	3.71	−0.9
**13**	−6.2391	S 27	O THR 163	H-donor	4.07	−0.6
**14**	−6.9892	5-ring	CB TRP 59	*π*-H	4.48	−0.9
**15**	−6.1362	N 20	O THR 163	H-donor	3.02	−4.7
		S 27	O TRP 59	H-donor	4.28	−0.5
**16**	−6.6492	S 26	OD1 ASP 356	H-donor	4.41	−0.4
**17**	−6.5001	S 26	OD1 ASP 197	H-donor	4.23	−1.5
		6-ring	6-ring TRP 59	*π*-*π*	3.51	−0.0
		6-ring	5-ring TRP 59	*π*-*π*	3.63	−0.0
		6-ring	5-ring TRP 59	*π*-*π*	3	−0.0
**18**	−6.4289	O 41	OD1 ASP 197	H-donor	1.7	−5.3
		6-ring	6-ring TRP 59	*π*-*π*	3.75	−0.0
		6-ring	5-ring TRP 59	*π*-*π*	3.78	−0.0
**19**	−9.7919	C 1	OD1 ASP 197	H-donor	2.2	−0.3
		N 19	OD1 ASP 356	H-donor	3.3	−0.5
		O 15	CD2 HIS 305	H-acceptor	3.8	−0.3
		5-ring	6-ring TRP 59	*π*-*π*	3.79	−0.0
**20**	−8.9694	C 1	OD1 ASP 197	H-donor	2.4	−0.3
		C 4	OD1 ASP 300	H-donor	1.9	−0.0
		C 8	5-ring HIS 101	H-*π*	4.87	−0.2
		5-ring	5-ring TRP 59	*π*-*π*	3.94	−0.0
**21**	−8.5183	C 28	OD1 ASP 300	H-donor	2.4	−1
		S 30	OD2 ASP 356	H-donor	3.24	−1.6
		C 16	6-ring TRP 59	H-*π*	3.6	−0.3
		N 19	5-ring TRP 59	H-*π*	3.47	−1.9
		6-ring	6-ring TRP 58	*π*-*π*	4.6	−0.2
**22**	−8.2417	C 8	OD1 ASP 356	H-donor	2.31	−0.5
		C 24	OD1 ASP 197	H-donor	3.33	−0.3
		S 26	O TYR 62	H-donor	3.58	−0.8
		CL 40	OE1 GLU 233	H-donor	3.31	−1.4
		6-ring	CB ALA 198	*π*-H	3.66	−0.2
**23**	−7.3154	C 24	OD2 ASP 197	H-donor	2.96	−1.8
**24**	−7.3456	C 24	OD1 ASP 300	H-donor	3.02	−1.6
**25**	−6.5103	5-ring	6-ring TRP 59	*π*-*π*	3.92	−0.0
**26**	−6.8201	O 15	OG1 THR 163	H-acceptor	3.16	−0.7
**27**	−6.7643	S 26	OD1 ASP 197	H-donor	2.9	−1.3
		5-ring	CD2 LEU 162	*π*-H	4.34	−0.6
**Standard**	−11.843	C 19	OD1 ASP 300	H-donor	3.42	−0.7
		C 36	OD1 ASP 300	H-donor	3.09	−1.2
		C 36	OD2 ASP 300	H-donor	2.96	−0.8
		O 61	OD1 ASP 197	H-donor	2.67	−4.2
		O 65	OD1 ASP 356	H-donor	2.83	−3.3
		O 69	OD1 ASP 197	H-donor	2.6	−1.6
		O 79	OG1 THR 163	H-donor	2.81	−1.8
		C 5	6-ring TRP 59	H-π	3.64	−0.6

**Figure 8:**
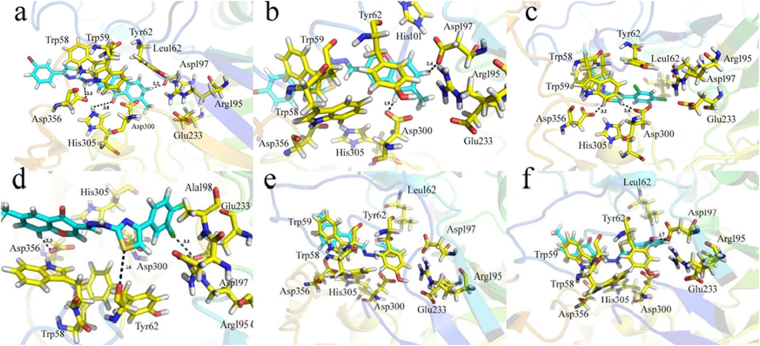
Docking conformations of compounds on *α*-amylase enzyme. (**a**) 3D binding mode of compound **19**. (**b**) 3D binding mode of compound **20**. (**c**) 3D binding mode of compound **21**. (**d**) 3D binding mode of compound **22**. (**e**) 3D binding mode of compound **17**. (**f**) 3D binding mode of compound **18** in binding cavity of *α*-amylase enzyme. Ligands are shown in cyan color.

Compound **19** has shown good interactions with the active site residues of the receptor protein Asp197, His305 and Asp356 (Fig. 8a). Asp197 formed strong H-donor interaction with the compound and His305 is involved in a strong H-acceptor bond of E**-**0.3 Kcal/mol (Table 2). Asp356 formed H-donor interaction with the -NH group of the ligand while Trp59 formed arene-arene linkage with the thiazole moiety of the compound. Compound **20** formed two H-donor, one H-π and one arene-arene valuable interactions with the enzyme. Asp197 and Asp300 showed H-donor interactions with the compound. Trp59 and His101 formed arene-arene and cation-π contact with the thiazole and benzene moiety of compound (Fig. 8b). The good inhibitory potency of the compound **19** is due to the different position of the bromine atom as compared to compound **20**. Presence of electronegative groups like halogens, observed to be actively participated in the activity and among halogens, Br containing compounds were found superior than Cl.

In case of compounds **21** and **22**, it was observed that both compounds have almost similar structure, biological activities and also similar binding interactions with the polar residues. Docking conformation of compound **21** showed that it was making two H-donor, two cation-π and one arene-arene contacts with the active residues of the enzyme (Fig. 8c). Compound **22** formed four H-donor and one *π*-H interactions with the Tyr62, Asp197, Glu233, Asp356 and Ala198 residues of the enzyme, respectively (Fig. 8d). The good inhibitory potential of the compound **21** over compound **22** is due to the diverse positions of the halogen group (-Cl).

**Figure 9:**
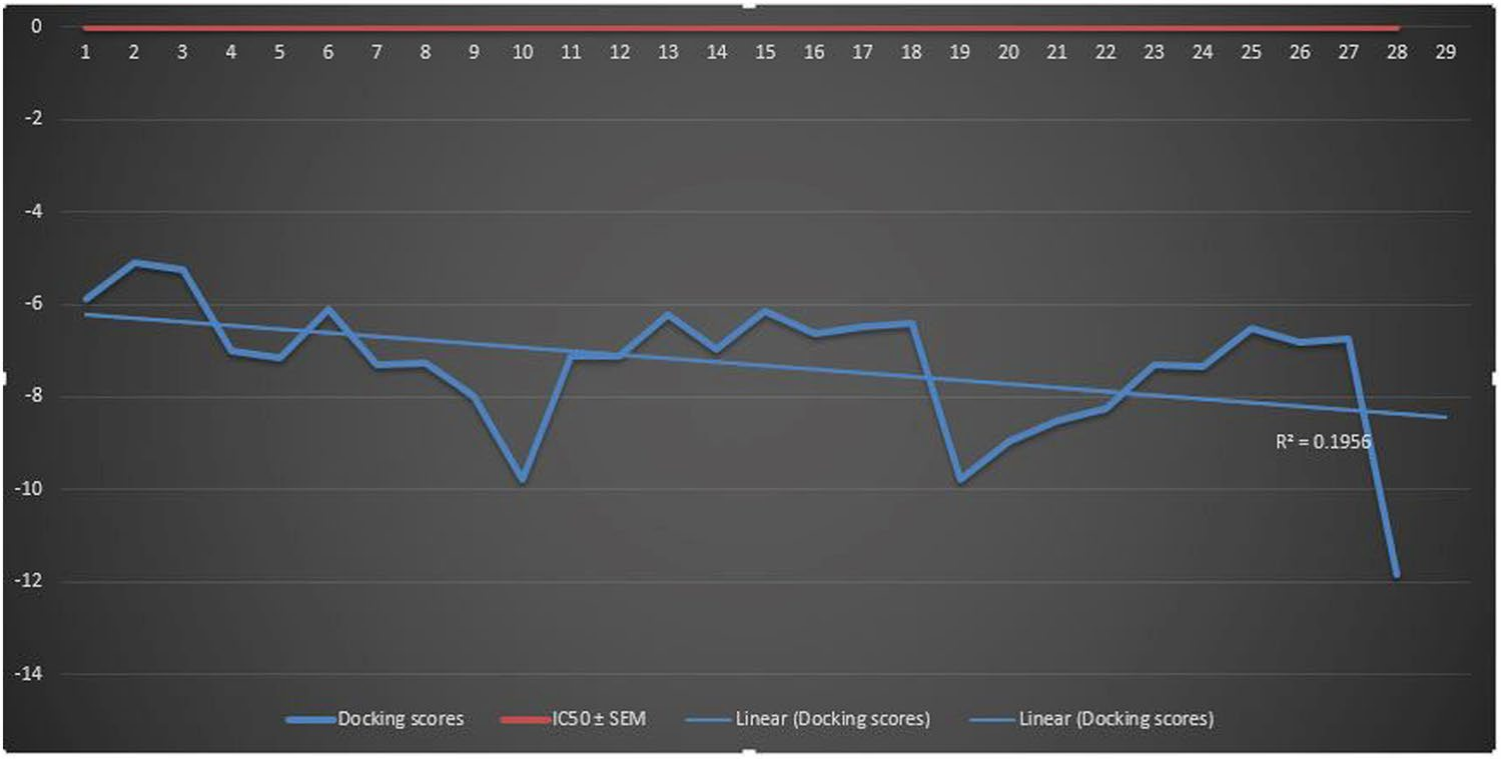
A correlation graph for predicted docking score and IC_50_ values.

The compounds having moderate biological activities such as **17** and **18**, having similar structure demonstrated almost similar binding pattern as shown in Table 2 and Fig. 8e and f. The more effectiveness of the compound **17** as compared to the compound **18** is due to the electronegative OH group at *meta* position. Overall a good correlation was observed between the docking study and biological evaluation of active compounds. The correlation graph and the correlation coefficient values are given in Fig. 9.

## Conclusion

New synthetic hybrid molecules of hydrazinyl thiazole substituted chromones **5–27** along with intervening intermediates **1**–**4** were evaluated for *in vitro α*-amylase inhibitory, DPPH and ABTS radical scavenging activities. Limited structure-activity relationship revealed that the compounds bearing halogen were found to be more active than the other groups such as OH, CN, NO_2_, and Ph, and compounds with methyl group as R_1_ were also found better active than the compounds without methyl substitution. All compounds showed good activities as compared to respective standards and also found to be non-toxic. Current study has identified a whole series of lead molecules which can be used in further advance research in order to obtain a powerful inhibitor for *α*-amylase enzyme for the development of insulin-independent antidiabetic agents.

## Experimental

### Materials and Methods

All chemicals were purchased from Sigma-Aldrich, USA. All reagents were of analytical grade and used as received. ^1^H and ^13^C-NMR experiments were performed on Avance Bruker AM 300, 400, and 500 MHz instruments. Electron impact mass spectrometric (EI-MS and HREI-MS) experiments were carried out on Finnigan MAT-311A (Germany) mass spectrometer. Thin layer chromatography (TLC) was performed on pre-coated silica gel aluminum plates (Kieselgel 60, 254, E. Merck, Germany). TLC chromatograms were visualized under UV light at 254 and 365 nm or by applying iodine vapors. Melting points of the compounds were determined on a Stuart^®^ SMP10 melting point apparatus and are uncorrected.

### General procedure for the synthesis of 3-formyl chromone derivatives 1 and 2

Dry dimethyl formamide (50 mmol) was taken in round-bottomed flask of 250 mL and 50 mmol of POCl_3_ was added drop wise into it with constant stirring at room temperature. After the complete addition of POCl_3_, reaction mixture was heated for at least 2 h at 50 °C. Then 2-hydroxy acetophenone/2-hydroxy-5-methyl acetophenone was added into it and further heated for 5 h at 70 °C. Reaction completion was monitored by TLC.

### General procedure for the synthesis of thiosemicarbazone derivatives of chromone 3 and 4

Chromone derivatives **1**/**2** (1 mmol) and thiosemicarbazide (1 mmol) were taken in 15 mL of ethanol into a 100 mL round-bottommed flask. Then few drops of glacial acetic acid were added into the reaction mixture and refluxed for 2 h. Course of reaction was checked by TLC analysis. Precipitates were appeared in the reaction flask which were collected *via* filtration, washed with distilled water, and dried in air. Solid products were crystallized from ethyl acetate.

### General procedure for the synthesis of hybrid hydrazinyl thiazole chromones 5–27

Thiosemicarbazone derivative **3**/**4** (1 mmol) and phenacyl bromide derivative (1 mmol) were taken in 15 mL of ethanol into a 100 mL round-bottommed flask. Triethylamine (1 mmol) was added into the reaction mixture and refluxed for 3 to 4 h. Completion of reaction was checked by TLC analysis. After reaction completion, reaction flask was kept overnight at room temperature. Precipitates were appeared in the reaction flask which were filtered, washed with distilled water, and dried in air. Solid compounds were crystallized from ethyl acetate.

### 4-Oxo-4*H*-chromene-3-carbaldehyde (1)

Yield: 75%; M.p.: 150–153 °C; ^1^H-NMR (400 MHz, DMSO-*d*_6_): *δ* 10.11 (s, 1 H, H-C=O), 8.91 (s, 1 H, H-2), 8.15 (dd, *J*_5,7_ = 1.2, *J*_5,6_ = 8.0 Hz, 1 H, H-5), 7.90 (dt, *J*_7,5_ = 1.6, *J*_7,6_ = *J*_7,8_ = 8.4 Hz, 1 H, H-7), 7.76 (d, *J*_8,7_ = 8.5 Hz, 1 H, H-8), 7.60 (t, *J*_6,5_ = *J*_6,7_ = 7.6 Hz, 1 H, H-6); ^13^C-NMR (300 MHz, DMSO-*d*_6_):*δ* 181.3, 174.8, 163.4, 155.5, 135.1, 126.6, 125.2, 124.6, 119.9, 118.8; EI-MS *m/z* (% rel. abund.): 174 (M^+^, 7), 146 (100), 120 (76), 104 (76), 92 (42); HREI-MS Calcd for C_10_H_7_O_3_: *m/z* = 174.0317, found 174.0382.

### 6-Methyl-4-oxo-4*H*-chromene-3-carbaldehyde (2)

Yield: 73%; M.p.: 173–175 °C; ^1^H-NMR (400 MHz, DMSO-*d*_6_): *δ* 10.11 (s, 1 H, H-C=O), 8.89 (s, 1 H, H-2), 7.93 (bd.s, 1 H, H-5), 7.71 (m, 2 H, H-7, 8), 2.44 (s, 3 H, CH_3_); ^13^C-NMR (400 MHz, DMSO-*d*_6_):*δ* 188.3, 174.8, 163.1, 153.8, 136.4, 136.0, 124.5, 124.3, 119.8, 118.6, 20.3; EI-MS *m/z* (% rel. abund.): 188 (M^+^, 13), 160 (100), 134 (95), 118 (37), 106 (25), 90 (48); HREI-MS Calcd for C_11_H_8_O_3_: *m/z* = 188.0473, found 188.0483.

### (*Z*)-2-((4-Oxo-4*H*-chromen-3-yl)methylene) hydrazinecarbothioamide (3)

Yield: 72%; M.p.: 240–242 °C; ^1^H-NMR (400 MHz, DMSO-*d*_6_): *δ* 11.52 (s, 1 H, NH), 9.16 (s, 1 H, -N=CH-), 8.24 (s, 1 H, NH), 8.17 (s, 1 H, NH), 8.11 (dd, *J*_5,6_ = 0.8, *J*_5,7_ = 8.0 Hz, 1 H, H-5), 8.08 (s, 1 H, H-2), 7.85 (dt, *J*_7,5_ = 1.2, *J*_7,6_ = *J*_7,8_ = 8.4 Hz, 1 H, H-7), 7.72 (d, *J*_8,7_ = 8.4 Hz, 1 H, H-8), 7.55 (t, *J*_6,5_ = *J*_6,7_ = 7.6 Hz, 1 H, H-6); ^13^C-NMR (400 MHz, DMSO-*d*_6_):*δ* 178.0, 174.7, 155.7, 155.1, 134.4, 133.9, 125.9, 125.1, 123.3, 118.6, 118.3; EI-MS *m/z* (% rel. abund.): 247 (M^+^, 34), 205 (42), 172 (100), 146 (16), 120 (47), 92 (39); HREI-MS Calcd for C_11_H_9_N_3_O_2_S: *m/z* = 247.0415, found 247.0413.

### (*Z*)-2-((6-Methyl-4-oxo-4*H*-chromen-3-yl)methylene)hydrazinecarbothioamide (4)

Yield: 74%; M.p.: 245–247 °C; ^1^H-NMR (300 MHz, DMSO-*d*_6_): *δ* 11.51 (s, 1 H, NH), 9.13 (s, 1 H, -N=CH-), 8.23 (s, 1 H, NH), 8.17 (s, 1 H, NH), 8.06 (bd.s, 1 H, H-5), 7.88 (s, 1 H, H-2), 7.66 (m, 2 H, H-7, 8), 2.43 (s, 3 H, CH_3_); ^13^C-NMR (400 MHz, DMSO-*d*_6_):*δ* 178.0, 174.6, 155.0, 154.0, 135.6, 135.5, 134.1, 124.3, 123.0, 118.4, 118.1, 20.4; EI-MS *m/z* (% rel. abund.): 261 (M^+^, 45), 219 (34), 202 (30), 186 (100), 160 (10), 134 (81); HREI-MS Calcd for C_12_H_11_N_3_O_2_S: *m/z* = 261.0572, found 261.0557.

### (*Z*)-3-((2-(4-Phenylthiazol-2-yl)hydrazono)methyl)-4*H*-chromen-4-one (5)

Yield: 70%; M.p.: 223–225 °C; ^1^H-NMR (400 MHz, DMSO-*d*_6_): *δ* 12.23 (s, 1 H, NH), 8.72 (s, 1 H, -N=CH-), 8.14 (s, 1 H, H-5′), 8.14 (d, *J*_5,6_ = 8.0 Hz, 1 H, H-5), 7.85 (d, *J*_2′′,3′′/6′′,5′′_ = 7.2 Hz, 3 H, H-7, 2′′, 6′′), 7.72 (d, *J*_8,7_ = 8.4 Hz, 1 H, H-8), 7.56 (t, *J*_6,5_ = *J*_6,7_ = 7.6 Hz, 1 H, H-6), 7.41 (t, *J*_3′′,2′′/5′′,6′′_ = *J*_3′′,4′′/5′′,4′′_ = 7.2 Hz, 2 H, H-3′′, 5′′), 7.33 (s, 1 H, H-2), 7.31 (t, *J*_4′′,3′′_ = *J*_4′′,5′′_ = 11.2 Hz, 1 H, H-4′′); ^13^C-NMR (400 MHz, DMSO-*d*_6_):*δ* 174.5, 171.1, 155.6, 155.2, 150.3, 134.5, 133.8, 133.2, 129.1, 129.1, 128.6, 127.4, 127.4, 125.8, 125.2, 123.4, 118.5, 118.2, 105.1; EI-MS *m/z* (% rel. abund.): 347 (M^+^, 100), 227 (65), 200 (11), 176 (52), 146 (21); HREI-MS Calcd for C_19_H_13_N_3_O_2_S: *m/z* = 347.0728, found 347.0731.

### (*Z*)-3-((2-(4-(3-Hydroxyphenyl)thiazol-2-yl)hydrazono)methyl)-4*H*-chromen-4-one (6)

Yield: 80%; M.p.: 235–237 °C; ^1^H-NMR (400 MHz, DMSO-*d*_6_): *δ* 11.52 (s, 1 H, NH), 9.16 (s, 1 H, -N=CH-), 8.24 (bd.s, 1 H, H-2′′), 8.17 (s, 1 H, H-5′), 8.11 (m, 4 H, H-5, 4′′, 5′′, 6′′), 7.85 (t, *J*_7,6_ = *J*_7,8_ = 8.0 Hz, 1 H, H-7), 7.72 (d, *J*_8,7_ = 8.4 Hz, 2 H, H-2, 8), 7.55 (t, *J*_6,5_ = *J*_6,7_ = 7.6 Hz, 1 H, H-6); ^13^C-NMR (300 MHz, DMSO-*d*_6_):*δ* 177.8, 171.4, 157.3, 155.8, 155.1, 150.1, 134.7, 134.3, 133.7, 130.5, 125.8, 125.0, 123.2, 120.2, 118.8, 118.2, 116.1, 115.7, 105.2; EI-MS *m/z* (% rel. abund.): 247 (28), 213 (8), 205 (45), 188 (34), 172 (85), 146 (16), 120 (100); HREI-MS Calcd for C_11_H_9_N_3_O_2_S: *m/z* = 247.0415, found 247.0404.

### (*Z*)-3-((2-(4-(4-Bromophenyl)thiazol-2-yl)hydrazono)methyl)-4*H*-chromen-4-one (7)

Yield: 75%; M.p.: 230–232 °C; ^1^H-NMR (400 MHz, DMSO-*d*_6_): *δ* 12.23 (s, 1 H, NH), 8.72 (s, 1 H, -N=CH-), 8.15 (s, 1 H, H-5′), 8.13 (d, *J*_5,6_ = 8.0 Hz, 1 H, H-5), 7.86 (t, *J*_7,6_ = *J*_7,8_ = 7.2 Hz, 1 H, H-7), 7.80 (d, *J*_3′′,2′′/5′′,6′′_ = 8.4 Hz, 2 H, H-3′′, 5′′), 7.72 (d, *J*_8,7_ = 8.4 Hz, 1 H, H-8), 7.60 (d, *J*_2′′,3′′/6′′,5′′_ = 8.4 Hz, 2 H, H-2′′, 6′′), 7.56 (t, *J*_6,5_ = *J*_6,7_ = 7.6 Hz, 1 H, H-6), 7.41 (s, 1 H, H-2); ^13^C-NMR (400 MHz, DMSO-*d*_6_):*δ* 177.0, 171.3, 155.8, 155.0, 150.4, 134.7, 133.8, 132.2, 131.5, 131.5, 127.5, 127.5, 125.8, 125.0, 123.4, 123.1, 118.7, 118.2, 105.2; EI-MS *m/z* (% rel. abund.): 425 (M^+^, 97), 427 (M + 2, 100), 305 (76), 280 (9), 254 (40), 212 (12), 146 (30); HREI-MS Calcd for C_19_H_12_BrN_3_O_2_S: *m/z* = 424.9834, found 424.9806.

### (*Z*)-3-((2-(4-(3-Bromophenyl)thiazol-2-yl)hydrazono)methyl)-4*H*-chromen-4-one (8)

Yield: 78%; M.p.: 225–227 °C; ^1^H-NMR (400 MHz, DMSO-*d*_6_): *δ* 12.25 (s, 1 H, NH), 8.72 (s, 1 H, -N=CH-), 8.15 (s, 1 H, H-5′), 8.13 (d, *J*_5,6_ = 8.8 Hz, 1 H, H-5), 8.03 (bd.s, 1 H, H-2′′), 7.86 (m, 2 H, H-4′′, 5′′), 7.72 (d, *J*_6′′,5′′_ = 8.0 Hz, 1 H, H-6′′), 7.56 (t, *J*_7,6_ = *J*_7,8_ = 7.2 Hz, 1 H, H-7), 7.49 (bd.s, 1 H, H-2), 7.49 (d, *J*_8,7_ = 9.2 Hz, 1 H, H-8), 7.38 (t, *J*_6,5_ = *J*_6,7_ = 8.0 Hz, 1 H, H-6); ^13^C-NMR (400 MHz, DMSO-*d*_6_):*δ* 177.2, 171.3, 155.8, 155.3, 150.1, 134.9, 122.2, 134.3, 133.7, 129.8, 129.4, 128.7, 125.8, 125.5, 125.0, 123.2, 118.7, 118.2, 105.1; EI-MS *m/z* (% rel. abund.): 425 (M^+^, 93), 427 (M + 2, 100), 410 (11), 307 (78), 280 (11), 254 (42), 172 (26), 146 (28); HREI-MS Calcd for C_19_H_12_BrN_3_O_2_S: *m/z* = 424.9834, found 424.9842.

### (*Z*)-3-((2-(4-(3,4-Dichlorophenyl)thiazol-2-yl)hydrazono)methyl)-4*H*-chromen-4-one (9)

Yield: 73%; M.p.: 245–247 °C; ^1^H-NMR (400 MHz, DMSO-*d*_6_): *δ* 12.26 (s, 1 H, NH), 8.73 (s, 1 H, -N=CH-), 8.15 (s, 1 H, H-5′), 8.13 (d, *J*_5,6_ = 8.0 Hz, 1 H, H-5), 8.07 (d, *J*_2′′,6′′_ = 2.0 Hz, 1 H, H-2′′), 7.85 (m, 2 H, H-7, 5′′), 7.72 (d, *J*_6′′,5′′_ = 8.4 Hz, 1 H, H-6′′), 7.67 (d, *J*_8,7_ = 8.8 Hz, 1 H, H-8), 7.56 (s, 1 H, H-2), 7.56 (t, *J*_6,5_ = *J*_6,7_ = 8.0 Hz, 1 H, H-6); ^13^C-NMR (400 MHz, DMSO-*d*_6_):*δ* 177.7, 171.4, 155.8, 155.2, 150.4, 134.5, 133.7, 133.1, 132.6, 132.3, 130.8, 128.9, 127.2, 125.7, 125.3, 123.2, 118.7, 118.4, 105.1; EI-MS *m/z* (% rel. abund.): 415 (M^+^, 94), 417 (M + 2, 64), 295 (100), 244 (46), 208 (12), 172 (21), 146 (17), 120 (13); HREI-MS Calcd for C_19_H_12_Cl_2_N_3_O_2_S: *m/z* = 414.9949, found 414.9956.

### (*Z*)-3-((2-(4-(2,4-Dichlorophenyl)thiazol-2-yl)hydrazono)methyl)-4*H*-chromen-4-one (10)

Yield: 77%; M.p.: 243–245 °C; ^1^H-NMR (400 MHz, DMSO-*d*_6_): *δ* 12.24 (s, 1 H, NH), 8.73 (s, 1 H, -N=CH-), 8.15 (s, 1 H, H-5′), 8.13 (d, *J*_5,6_ = 8.0 Hz, 1 H, H-5), 7.90 (d, *J*_6′′,5′′_ = 8.8 Hz, 1 H, H-6′′), 7.87 (t, *J*_7,6_ = *J*_7,8_ = 11.2 Hz, 1 H, H-7), 7.72 (d, *J*_8,7_ = 8.4 Hz, 1 H, H-8), 7.68 (d, *J*_3′′,5′′_ = 2.0 Hz, 1 H, H-3′′), 7.56 (t, *J*_6,5_ = *J*_6,7_ = 7.6 Hz, 1 H, H-6), 7.51 (dd, *J*_5′′,3′′_ = 2.0, *J*_5′′,6′′_ = 8.4 Hz, H-5′′), 7.42 (s, 1 H, H-2); ^13^C-NMR (400 MHz, DMSO-*d*_6_):*δ* 177.7, 171.1, 155.5, 155.2, 150.0, 135.8, 134.3, 133.8, 133.4, 130.8, 130.1, 128.2, 127.5, 125.8, 125.0, 123.4, 118.7, 118.2, 105.1; EI-MS *m/z* (% rel. abund.): 415 (M^+^, 61), 417 (M + 2, 49), 380 (50), 295 (100), 244 (41), 202 (23), 172 (27); HREI-MS Calcd for C_19_H_12_Cl_2_N_3_O_2_S: *m/z* = 414.9949, found 414.9936.

### (*Z*)-3-((2-(4-(4-Chlorophenyl)thiazol-2-yl)hydrazono)methyl)-4*H*-chromen-4-one (11)

Yield: 74%; M.p.: 239–241 °C; ^1^H-NMR (400 MHz, DMSO-*d*_6_): *δ* 12.23 (s, 1 H, NH), 8.72 (s, 1 H, -N=CH-), 8.15 (s, 1 H, H-5′), 8.13 (d, *J*_5,6_ = 8.0 Hz, 1 H, H-5), 7.87 (d, *J*_2′′,3′′/6′′,5′′_ = 8.4 Hz, 2 H, H-3′′, 5′′), 7.87 (t, *J*_6,5_ = *J*_6,7_ = 6.8 Hz, 1 H, H-6), 7.72 (d, *J*_8,7_ = 8.4 Hz, 1 H, H-8), 7.56 (t, *J*_7,6_ = *J*_7,8_ = 7.6 Hz, 1 H, H-7), 7.46(d, *J*_3′′,2′′/5′′,6′′_ = 8.4 Hz, 2 H, H-2′′, 6′′), 7.40 (s, 1 H, H-2); ^13^C-NMR (400 MHz, DMSO-*d*_6_):*δ* 177.8, 171.6, 155.8, 155.0, 150.0, 134.5, 134.2, 133.8, 131.2, 128.5, 128.5, 127.1, 127.1, 125.7, 125.0, 123.4, 118.7, 118.2, 105.1; EI-MS *m/z* (% rel. abund.): 381 (M^+^, 100), 383 (M + 2, 38), 261 (95), 234 (7), 210 (37), 168 (19), 146 (13); HREI-MS Calcd for C_19_H_12_ClN_3_O_2_S: *m/z* = 381.0339, found 381.0323.

### (*Z*)-3-((2-(4-(3-Chlorophenyl)thiazol-2-yl)hydrazono)methyl)-4*H*-chromen-4-one (12)

Yield: 75%; M.p.: 228–230 °C; ^1^H-NMR (400 MHz, DMSO-*d*_6_): *δ* 12.25 (s, 1 H, NH), 8.72 (s, 1 H, -N=CH-), 8.15 (s, 1 H, H-5′), 8.13 (d, *J*_5,6_ = 8.0 Hz, 1 H, H-5), 7.88 (bd.s, 1 H, H-2′′), 7.87 (m, 2 H, H-4′′, 5′′), 7.72 (d, *J*_8,7_ = 8.4 Hz, 1 H, H-8), 7.56 (t, *J*_7,6_ = *J*_7,8_ = 7.6 Hz, 1 H, H-7), 7.50 (bd.s, 1 H, H-2), 7.45 (t, *J*_6,5_ = *J*_6,7_ = 7.6 Hz, 1 H, H-6), 7.36 (d, *J*_6′′,5′′_ = 8.0 Hz, 1 H, H-6′′); ^13^C-NMR (400 MHz, DMSO-*d*_6_):*δ* 176.7, 170.9, 155.7, 153.5, 148.9, 136.6, 134.5, 133.7, 133.4, 130.4, 127.2, 125.9, 125.2, 125.1, 124.0, 123.2, 118.6, 118.4, 105.4; EI-MS *m/z* (% rel. abund.): 381 (M^+^, 100), 383 (36), 261 (94), 234 (8), 210 (49), 172 (21), 146 (26); HREI-MS Calcd for C_19_H_12_ClN_3_O_2_S: *m/z* = 381.0339, found 381.0307.

### (*Z*)-3-((2-(4-(3-Nitrophenyl)thiazol-2-yl)hydrazono)methyl)-4*H*-chromen-4-one (13)

Yield: 71%; M.p.: 235–237 °C; ^1^H-NMR (400 MHz, DMSO-*d*_6_): *δ* 12.35 (s, 1 H, NH), 8.74 (s, 1 H, -N=CH-), 8.66 (s, 1 H, H-2′′), 8.30 (d, *J*_5,6_ = 7.6 Hz, 1 H, H-5), 8.16 (m, 3 H, H-5′, 4′′, 5′′), 7.87 (t, *J*_7,6_ = *J*_7,8_ = 6.8 Hz, 1 H, H-7), 7.72 (d, *J*_8,7_ = *J*_6′′,5′′_ = 8.4 Hz, 2 H, H-8, 6′′),7.67 (bd.s, 1 H, H-2), 7.56 (t, *J*_6,5_ = *J*_6,7_ = 7.6 Hz, 1 H, H-6); ^13^C-NMR (400 MHz, DMSO-*d*_6_):*δ* 177.4, 171.4, 155.8, 155.2, 150.3, 148.5, 134.3, 133.8, 133.7, 133.5, 130.5, 125.8, 125.2, 123.8, 123.4, 122.6, 118.7, 118.3, 105.2; EI-MS *m/z* (% rel. abund.): 392 (M^+^, 67), 375 (71), 272 (100), 221 (20), 172 (22), 146 (21), 120 (32); HREI-MS Calcd for C_19_H_12_N_4_O_4_S: *m/z* = 392.0579, found 392.0577.

### (*Z*)-4-(2-(2-((4-Oxo-4*H*-chromen-3-yl)methylene)hydrazinyl)thiazol-4-yl)benzonitrile (14)

Yield: 72%; M.p.: 225–227 °C; ^1^H-NMR (400 MHz, DMSO-*d*_6_): *δ* 12.30 (s, 1 H, NH), 8.73 (s, 1 H, -N=CH-), 8.16 (s, 1 H, H-5′), 8.13 (d, *J*_5,6_ = 6.8 Hz, 1 H, H-5), 8.03 (d, *J*_2′′,3′′/6′′,5′′_ = 8.4 Hz, 2 H, H-2′′, 6′′), 7.87 (d, *J*_3′′,2′′/5′′,6′′_ = 8.4 Hz, 3 H, H-7, 3′′, 5′′), 7.72 (d, *J*_8,7_ = 8.4 Hz, 1 H, H-8), 7.65 (s, 1 H, H-2), 7.56 (t, *J*_6,5_ = *J*_6,7_ = 7.2 Hz, 1 H, H-6); ^13^C-NMR (400 MHz, DMSO-*d*_6_):*δ* 177.9, 171.6, 155.8, 155.2, 150.3, 140.9, 140.7, 134.5, 133.8, 131.7, 129.3, 129.3, 128.1, 128.1, 127.9, 127.9, 127.6, 127.1, 127.1, 125.8, 125.2, 123.4, 118.7, 118.2, 105.3; EI-MS *m/z* (% rel. abund.): 372 (M^+^, 92), 252 (100), 225 (8), 201 (43), 172 (17), 159 (23), 146 (14); HREI-MS Calcd for C_20_H_12_N_4_O_2_S: *m/z* = 372.0681, found 372.0669.

### (*Z*)-3-((2-(4-(Biphenyl-4-yl)thiazol-2-yl)hydrazono)methyl)-4*H*-chromen-4-one (15)

Yield: 79%; M.p.: 234–236 °C; ^1^H-NMR (400 MHz, DMSO-*d*_6_): *δ* 12.25 (s, 1 H, NH), 8.73 (s, 1 H, -N=CH-), 8.16 (s, 1 H, H-5′), 8.14 (d, *J*_5,6_ = 7.2 Hz, 1 H, H-5), 7.95 (d, *J*_2′′,3′′/6′′,5′′_ = 8.0 Hz, 2 H, H-2′′, 6′′), 7.85 (t, *J*_7,6_ = *J*_7,8_ = 7.2 Hz, 1 H, H-7), 7.72 (bd.d, *J*_3′′,2′′/5′′,6′′/2′′′,3′′′/6′′′,5′′′_ = 8.8 Hz, 4 H, H-3′′, 5′′, 2′′′, 6′′′), 7.56 (t, *J*_6,5_ = *J*_6,7_ = *J*_4′′′,3′′′_ = *J*_4′′′,5′′′_ = 7.6 Hz, 1 H, H-6, 4′′′), 7.49 (t, *J*_3′′′,2′′′/5′′′,6′′′_ = 7.6 Hz, 1 H, H-3′′′,5′′′), 7.40 (s, 1 H, H-2), 7.38 (d, *J*_8,7_ = 7.2 Hz, 1 H, H-8); ^13^C-NMR (400 MHz, DMSO-*d*_6_):*δ* 177.7, 171.4, 155.8, 155.2, 150.3, 135.8, 134.5, 133.9, 133.5, 130.8, 130.2, 128.1, 127.5, 125.8, 125.2, 123.4, 118.7, 118.2, 105.1; EI-MS *m/z* (% rel. abund.): 423 (M^+^, 100), 303 (48), 252 (35), 210 (23), 178 (8), 165 (7), 146 (6); HREI-MS Calcd for C_25_H_17_N_3_O_2_S: *m/z* = 423.0810, found 423.0820.

### (*Z*)-6-Methyl-3-((2-(4-phenylthiazol-2-yl)hydrazono)methyl)-4*H*-chromen-4-one (16)

Yield: 79%; M.p.: 235–237 °C; ^1^H-NMR (400 MHz, DMSO-*d*_6_): *δ* 12.21 (s, 1 H, NH), 8.69 (s, 1 H, -N=CH-), 8.14 (s, 1 H, H-5′), 7.91 (bd.s, 1 H, H-5), 7.85 (d, *J*_2′′,3′′/6′′,5′′_ = 8.0 Hz, 2 H, H-2′′, 6′′), 7.65 (m, 2 H, H-7, 8), 7.41 (t, *J*_3′′,2′′/5′′,6′′/3′′,4′′/5′′,4′′_ = 7.6 Hz, 2 H, H-3′′, 5′′), 7.33 (s, 1 H, H-2), 7.31 (t, *J*_4′′,3′′/4′′,5′′_ = 7.2 Hz, 1 H, H-4′′), 2.44 (s, 3 H, CH_3_); ^13^C-NMR (400 MHz, DMSO-*d*_6_):*δ* 177.8, 172.0, 155.1, 154.2, 150.1, 135.7, 135.4, 134.2, 133.1, 129.3, 129.3, 128.8, 127.6, 127.6, 124.4, 123.1, 118.4, 118.0, 105.2, 20.5; EI-MS *m/z* (% rel. abund.): 361 (M^+^, 100), 227 (100), 200 (7), 176 (32), 160 (16), 134 (57); HREI-MS Calcd for C_20_H_15_N_3_O_2_S: *m/z* = 361.0885, found 361.0900.

### (*Z*)-3-((2-(4-(3-Hydroxyphenyl)thiazol-2-yl)hydrazono)methyl)-6-methyl-4*H*-chromen-4-one (17)

Yield: 74%; M.p.: 237–239 °C; ^1^H-NMR (400 MHz, DMSO-*d*_6_): *δ* 11.51 (s, 1 H, NH), 9.13 (s, 1 H, -N=CH-), 8.23 (s, 1 H, H-5′), 8.17 (s, 1 H, H-2), 8.07 (bd.s, 1 H, H-5), 7.88 (s, 1 H, H-2′′), 7.66 (m, 5 H, H-7, 8, 4′′, 5′′, 6′′), 2.43 (s, 3 H, CH_3_); ^13^C-NMR (400 MHz, DMSO-*d*_6_):*δ* 177.9, 172.0, 157.6, 155.1, 154.2, 150.0, 135.7, 135.6, 134.5, 134.2, 130.7, 124.4, 123.2, 120.0, 118.5, 118.0, 115.9, 115.7, 105.1, 20.5; EI-MS *m/z* (% rel. abund.): 377 (M^+^, 2), 261 (27), 219 (28), 202 (34), 186 (80), 134 (100); HREI-MS Calcd for C_20_H_13_N_3_O_3_S: *m/z* = 377.0834, found 377.0830.

### (*Z*)-3-((2-(4-(2-Hydroxyphenyl)thiazol-2-yl)hydrazono)methyl)-6-methyl-4*H*-chromen-4-one (18)

Yield: 71%; M.p.: 230–232 °C; ^1^H-NMR (400 MHz, DMSO-*d*_6_): *δ* 11.51 (s, 1 H, NH), 9.13 (s, 1 H, -N=CH-), 8.23 (bd.s, 1 H, H-5), 8.17 (s, 1 H, H-5′), 8.16 (s, 1 H, H-2), 8.06 (bd.s, 1 H, H-8), 7.88 (bd.s, 1 H, H-7), 7.66 (m, 4 H, H-3′′, 4′′, 5′′, 6′′), 2.43 (s, 3 H, CH_3_); ^13^C-NMR (400 MHz, DMSO-*d*_6_):*δ* 178.0, 172.5, 155.4, 155.0, 154.2, 147.7, 135.7, 135.4, 134.2, 131.6, 130.2, 124.4, 123.1, 121.9, 120.6, 118.5, 118.0, 117.7, 105.2, 20.5; EI-MS *m/z* (% rel. abund.): 377 (M^+^, 10), 318 (7), 261 (16), 219 (14), 202 (36), 186 (38), 160 (10), 134 (100); HREI-MS Calcd for C_20_H_15_N_3_O_3_S: *m/z* = 377.0834, found 377.0831.

### (*Z*)-3-((2-(4-(3-Bromophenyl)thiazol-2-yl)hydrazono)methyl)-6-methyl-4*H*-chromen-4-one (19)

Yield: 71%; M.p.: 239–241 °C; ^1^H-NMR (400 MHz, DMSO-*d*_6_): *δ* 12.23 (s, 1 H, NH), 8.69 (s, 1 H, -N=CH-), 8.14 (s, 1 H, H-5′), 8.03 (s, 1 H, H-5), 7.91 (s, 1 H, H-2′′), 7.85 (d, *J*_8,7_ = 8.0, 1 H, H-8), 7.67 (m, 2 H, H-4′′, 6′′), 7.49 (s, 1 H, H-2), 7.49 (d, *J*_7,8_ = 7.2 Hz, 1 H, H-7), 7.38 (t, *J*_5′′,4′′_ = *J*_5′′,6′′_ = 8.0 Hz, 1 H, H-5′′), 2.44 (s, 3 H, CH_3_); ^13^C-NMR (400 MHz, DMSO-*d*_6_):*δ* 177.9, 170.7, 155.3, 154.2, 150.0, 135.7, 135.4, 135.3, 134.2, 131.6, 131.0, 128.2, 126.6, 124.2, 123.1, 122.3, 118.5, 118.0, 105.2, 20.6; EI-MS *m/z* (% rel. abund.): 439 (M^+^, 88), 441 (M + 2, 91), 307 (100), 254 (30), 227 (10), 186 (20), 160 (17), 134 (73); HREI-MS Calcd for C_20_H_14_BrN_3_O_2_S: *m/z* = 438.9990, found 438.9993.

### (*Z*)-3-((2-(4-(4-Bromophenyl)thiazol-2-yl)hydrazono)methyl)-6-methyl-4*H*-chromen-4-one (20)

Yield: 73%; M.p.: 235–237 °C; ^1^H-NMR (400 MHz, DMSO-*d*_6_): *δ* 12.22 (s, 1 H, NH), 8.69 (s, 1 H, -N=CH-), 8.14 (s, 1 H, H-5′), 7.91 (s, 1 H, H-5), 7.80 (d, *J*_2′′,3′′/6′′,5′′_ = 8.4 Hz, 2 H, H-2′′, 6′′), 7.67 (m, 4 H, H-7, 8, 3′′, 5′′), 7.41 (s, 1 H, H-2), 2.44 (s, 3 H, CH_3_); ^13^C-NMR (400 MHz, DMSO-*d*_6_):*δ* 177.9, 172.0, 155.2, 154.1, 150.3, 135.7, 135.6, 134.2, 132.2, 132.2, 132.0, 128.4, 128.4, 124.4, 123.2, 123.0, 118.5, 118.2, 105.1, 20.5; EI-MS *m/z* (% rel. abund.): 439 (M^+^, 88), 441 (M + 2, 90), 305 (100), 280 (5), 254 (3), 212 (9), 186 (13), 160 (15), 134 (37); HREI-MS Calcd for C_20_H_14_BrN_3_O_2_S: *m/z* = 438.9990, found 438.9992.

### (*Z*)-3-((2-(4-(2,4-Dichlorophenyl)thiazol-2-yl)hydrazono)methyl)-6-methyl-4*H*-chromen-4-one (21)

Yield: 78%; M.p.: 245–247 °C; ^1^H-NMR (400 MHz, DMSO-*d*_6_): *δ* 12.22 (s, 1 H, NH), 8.70 (s, 1 H, -N=CH-), 8.14 (s, 1 H, H-5′), 7.91 (s, 1 H, H-5), 7.89 (d, *J*_8,7_ = 8.4, 1 H, H-8), 7.68 (m, 3 H, H-7, 3′′, 6′′), 7.51 (d, *J*_5′′,6′′_ = 8.4 Hz, 1 H, H-5′′), 7.42 (s, 1 H, H-2), 2.44 (s, 3 H, CH_3_); ^13^C-NMR (400 MHz, DMSO-*d*_6_):*δ* 177.9, 172.1, 155.3, 154.2, 150.4, 135.6, 135.4, 134.0, 133.5, 132.8, 132.4, 130.8, 128.9, 127.1, 124.4, 123.1, 118.5, 118.0, 105.2, 20.3; EI-MS *m/z* (% rel. abund.): 429 (M^+^, 64), 431 (M + 2, 44), 394 (29), 295 (100), 244 (32), 202 (14), 186 (24), 160 (14), 144 (6), 134 (64); HREI-MS Calcd for C_20_H_13_Cl_2_N_3_O_2_S: *m/z* = 429.0106, found 429.0091.

### (*Z*)-3-((2-(4-(3,4-Dichlorophenyl)thiazol-2-yl)hydrazono)methyl)-6-methyl-4*H*-chromen-4-one (22)

Yield: 72%; M.p.: 246–248 °C; ^1^H-NMR (400 MHz, DMSO-*d*_6_): *δ* 12.25 (s, 1 H, NH), 8.70 (s, 1 H, -N=CH-), 8.17 (s, 1 H, H-5′), 8.07 (s, *J*_5,7_ = 4.0 Hz, 1 H, H-5), 7.91 (s, 1 H, H-2′′), 7.84 (dd, *J*_7,5_ = 1.6, *J*_7,8_ = 8.8 Hz, 1 H, H-7), 7.67 (d, *J*_5′′,6′′_ = *J*_6′′,5′′_ = 8.4 Hz, 2 H, H-5′′, 6′′), 7.62 (d, *J*_8,7_ = 8.0, 1 H, H-8), 7.55 (s, 1 H, H-2), 2.43 (s, 3 H, CH_3_); ^13^C-NMR (400 MHz, DMSO-*d*_6_):*δ* 177.8, 172.2, 155.3, 154.1, 150.0, 135.7, 135.5, 134.2, 133.5, 132.8, 132.4, 130.8, 128.9, 127.1, 124.4, 123.2, 118.5, 118.0, 105.2, 20.5; EI-MS *m/z* (% rel. abund.): 429 (M^+^, 86), 431 (M + 2, 61), 295 (100), 244 (27), 204 (12), 186 (15), 134 (54); HREI-MS Calcd for C_20_H_14_Cl_2_N_3_O_2_S: *m/z* = 429.0106, found 429.0098.

### (*Z*)-3-((2-(4-(4-Chlorophenyl)thiazol-2-yl)hydrazono)methyl)-6-methyl-4*H*-chromen-4-one (23)

Yield: 71%; M.p.: 245–247 °C; ^1^H-NMR (400 MHz, DMSO-*d*_6_): *δ* 12.22 (s, 1 H, NH), 8.69 (s, 1 H, -N=CH-), 8.14 (s, 1 H, H-5′), 7.91 (bd.s, 1 H, H-5), 7.86 (d, *J*_2′′,3′′/6′′,5′′_ = 8.8 Hz, 2 H, H-2′′, 6′′), 7.65 (m, 2 H, H-7, 8), 7.46 (d, *J*_3′′,2′′/5′′,6′′_ = 8.4 Hz, 2 H, H-3′′, 5′′), 7.40 (s, 1 H, H-2), 2.44 (s, 3 H, CH_3_); ^13^C-NMR (400 MHz, DMSO-*d*_6_):*δ* 177.8, 172.5, 155.3, 154.1, 150.0, 135.7, 135.6, 134.2, 133.6, 131.7, 128.5, 128.5, 127.1, 127.1, 124.2, 123.1, 118.5, 118.0, 105.1, 20.5; EI-MS *m/z* (% rel. abund.): 395 (M^+^, 60), 397 (M + 2, 23), 261 (100), 210 (27), 186 (14), 168 (18), 134(24); HREI-MS Calcd for C_20_H_14_ClN_3_O_2_S: *m/z* = 395.0495, found 395.0490.

### (*Z*)-3-((2-(4-(3-Chlorophenyl)thiazol-2-yl)hydrazono)methyl)-6-methyl-4*H*-chromen-4-one (24)

Yield: 75%; M.p.: 230–232 °C; ^1^H-NMR (400 MHz, DMSO-*d*_6_): *δ* 12.24 (s, 1 H, NH), 8.69 (s, 1 H, -N = CH−CH-), 8.15 (s, 1 H, H-5′), 7.91 (s, 1 H, H-5), 7.88 (s, 1 H, H-2′′), 7.82 (d, *J*_8,7_ = 7.6, 1 H, H-8), 7.67 (m, 2 H, H-4′′, 6′′), 7.49 (s, 1 H, H-2), 7.45 (t, *J*_5′′,4′′_ = *J*_5′′,6′′_ = 8.0 Hz, 1 H, H-5′′), 7.36 (d, *J*_7,8_ = 8.0 Hz, 1 H, H-7), 2.44 (s, 3 H, CH_3_); ^13^C-NMR (400 MHz, DMSO-*d*_6_):*δ* 177.8, 172.4, 155.2, 154.1, 150.0, 135.7, 135.4, 134.9, 134.5, 134.2, 129.7, 129.4, 128.9, 125.7, 124.4, 123.1, 118.5, 118.2, 105.2, 20.5; EI-MS *m/z* (% rel. abund.): 395 (M^+^, 99), 397 (M + 2, 54), 261 (100), 234 (9), 210 (47), 186 (17), 168 (28), 160 (17), 134 (54); HREI-MS Calcd for C_20_H_14_ClN_3_O_2_S: *m/z* = 395.0495, found 395.0482.

### (*Z*)-6-Methyl-3-((2-(4-(3-nitrophenyl)thiazol-2-yl)hydrazono)methyl)-4*H*-chromen-4-one (25)

Yield: 74%; M.p.: 240–242 °C; ^1^H-NMR (400 MHz, DMSO-*d*_6_): *δ* 12.23 (s, 1 H, NH), 8.69 (s, 1 H, -N=CH-), 8.17 (s, 1 H, H-5′), 8.03 (s, 1 H, H-5), 7.91 (s, 1 H, H-2′′), 7.85 (d, *J*_8,7_ = 8.0, 1 H, H-8), 7.67 (m, 2 H, H-4′′, 6′′), 7.49 (s, 1 H, H-2), 7.49 (d, *J*_7,8_ = 7.2 Hz, 1 H, H-7), 7.38 (t, *J*_5′′,4′′_ = *J*_5′′,6′′_ = 7.6 Hz, 1 H, H-5′′), 2.44 (s, 3 H, CH_3_); ^13^C-NMR (400 MHz, DMSO-*d*_6_):*δ* 177.9, 172.3, 155.3, 154.1, 150.3, 148.5, 135.7, 135.4, 134.2, 133.9, 133.5, 130.5, 124.4, 123.8, 123.2, 122.6, 118.5, 118.0, 105.1, 20.6; EI-MS *m/z* (% rel. abund.): 406 (M^+^, 59), 389 (25), 272 (34), 221 (18), 186 (15), 134 (100); HREI-MS Calcd for C_20_H_14_N_4_O_4_S: *m/z* = 406.0736, found 406.0730.

### (*Z*)-4-(2-(2-((6-Methyl-4-oxo-4*H*-chromen-3-yl)methylene)hydrazinyl)thiazol-4-yl)benzonitrile (26)

Yield: 68%; M.p.: 238–240 °C; ^1^H-NMR (400 MHz, DMSO-*d*_6_): *δ* 12.28 (s, 1 H, NH), 8.70 (s, 1 H, -N=CH-), 8.16 (s, 1 H, H-5′), 8.03 (d, *J*_2′′,3′′/6′′,5′′_ = 8.0 Hz, 2 H, H-2′′, 6′′), 7.91 (bd.s, 1 H, H-5), 7.86 (d, *J*_3′′,2′′/5′′,6′′_ = 8.4 Hz, 2 H, H-3′′, 5′′), 7.67 (m, 3 H, H-2, 7, 8), 2.44 (s, 3 H, CH_3_); ^13^C-NMR (400 MHz, DMSO-*d*_6_):*δ* 177.8, 171.8, 155.2, 154.1, 150.3, 137.4, 135.7, 135.5, 134.2, 132.8, 132.8, 126.0, 126.0, 124.4, 123.1, 118.7, 118.3, 118.0, 112.7, 105.1, 20.4; EI-MS *m/z* (% rel. abund.): 386 (M^+^, 100), 252 (37), 201 (28), 186 (28), 159 (47), 134 (82); HREI-MS Calcd for C_21_H_14_N_4_O_2_S: *m/z* = 386.0837, found 386.0824.

### (*Z*)-3-((2-(4-(Biphenyl-4-yl)thiazol-2-yl)hydrazono)methyl)-6-methyl-4*H*-chromen-4-one (27)

Yield: 76%; M.p.: 235–237 °C; ^1^H-NMR (400 MHz, DMSO-*d*_6_): *δ* 12.24 (s, 1 H, NH), 8.70 (s, 1 H, -N=CH-), 8.15 (s, 1 H, H-5′), 7.95 (d, *J*_2′′,3′′/6′′,5′′_ = 8.4 Hz, 2 H, H-2′′, 6′′), 7.92 (bd.s, 1 H, H-5), 7.72 (bd.d, *J*_3′′,2′′/5′′,6′′/2′′′,3′′′/6′′′,5′′′_ = 8.4 Hz, 4 H, H-3′′, 5′′, 2′′′, 6′′′), 7.68 (m, 2 H, H-7, 8), 7.49 (t, *J*_3′′,2′′/5′′,6′′_ = *J*_3′′,4′′/5′′,4′′_ = 7.6 Hz, 2 H, H-3′′, 5′′), 7.40 (s, 1 H, H-2), 7.38 (t, *J*_4′′,3′′/4′′,5′′_ = 7.6 Hz, 1 H, H-4′′′), 2.44 (s, 3 H, CH_3_); ^13^C-NMR (400 MHz, DMSO-*d*_6_):*δ* 177.9, 172.0, 155.1, 154.2, 150.0, 140.9, 140.6, 135.7, 135.5, 134.2, 131.8, 129.2, 129.2, 128.1, 128.1, 127.8, 127.8, 127.5, 127.0, 127.0, 124.2, 123.1, 118.5, 118.0, 105.1, 20.6; EI-MS *m/z* (% rel. abund.): 437 (M^+^, 100), 303 (80), 276 (4), 252 (25), 210 (15); HREI-MS Calcd for C_26_H_19_N_3_O_2_S: *m/z* = 437.1198, found 437.1205.

### *α*-Amylase inhibition assay

The *α*-amylase inhibitory activity was determined by an assay modified from Kwon, Apostolidis & Shetty^37,38^. A volume of 500 *µ*L of test sample (100 *µ*g/mL, 200 *µ*g/mL, 400 *µ*g/mL, 800 *µ*g/mL, 1000 *µ*g/mL) and 500 *µ*L of *α*-amylase solution (0.5 mg/mL) in 0.2 mM phosphate buffer (pH 6.9) were incubated at 25 °C for 10 min. After pre-incubation, 500 *μ*L of 1% starch solution in 0.02 M sodium phosphate buffer (pH 6.9) was added to each tube and incubated at 25 °C for 10 minutes. The reaction was arrested with 1 mL of dinitrosalicylic acid colour reagent. The tubes were then incubated in boiling water for 5 min and cooled to room temperature. The solutions were diluted after adding 10 mL distilled water and the absorbance was measured at 540 nm^39^.

The percentage of inhibition was calculated as illustrated,

$$ \% {\rm{Inhibition}}=({{\rm{Absorbance}}}_{{\rm{Control}}}-{{\rm{Absorbance}}}_{{\rm{Sample}}})/{{\rm{Absorbance}}}_{{\rm{Control}}}\,\times \,{\rm{100}}$$

The IC_50_ values, concentration required to inhibit the *α*-amylase activity by 50% were calculated by a non-linear regression graph plotted between percentage inhibition (x axis) versus concentrations (y axis), using a Graph Pad Prism Software (Version 5).

### DPPH Free radical scavenging assay

The ability of the sample to scavenge, 2-diphenyl-1-picrylhydrazyl (DPPH) free radicals was evaluated by standard method^40^. The sample solutions were prepared in absolute alcohol, ranging from 0.01 mg/mL to 1 mg/mL. A total of 500 *μ*L of sample was added with 500 *µ*L of 2 *µ*mol DPPH solution. After 20 min of incubation, the samples were placed in the dark at room temperature, the absorbance was taken at 517 nm. 500 *µ*L of prepared DDPH solution and 500 *µ*L of absolute alcohol were used as control. The similar procedure was repeated for ascorbic acid as standard^41,42^.

The percentage inhibition of radical scavenging activity was calculated as illustrated,

$$ \% {\rm{Inhibition}}=({{\rm{Absorbance}}}_{{\rm{Control}}}-{{\rm{Absorbance}}}_{{\rm{Sample}}})/{{\rm{Absorbance}}}_{{\rm{Control}}}\,\times \,100$$

### ABTS Free radical cation scavenging assay

The (ABTS+) 2,2ʹ-azino-*bis*(3-ethylbenzothiazoline-6-sulphonic acid) free radical cation scavenging ability of the compounds was determined by standard method^43^. 7 mM ABTS was dissolved in distilled water and 2.45 mM potassium persulfate was added. The solution was kept in the dark for 12–16 h at room temperature. The sample solutions were prepared in absolute alcohol ranging from 0.01 mg/mL to 1 mg/mL. The samples were added with ABTS solution and incubated for 30 min. The absorbance was taken at 734 nm and the procedure was repeated for ascorbic acid as standard.

The percentage inhibition of radical scavenging activity was calculated as illustrated,

$$ \% {\rm{Inhibition}}=({{\rm{Absorbance}}}_{{\rm{Control}}}-{{\rm{Absorbance}}}_{{\rm{Sample}}})/{{\rm{Absorbance}}}_{{\rm{Control}}}\,\times \,100$$

### MTT Cytotoxicity assay

Cytotoxicity of the newly synthesized compounds on NIH-3T3 fibroblast cells (ATCC, Manassas, USA) was checked by the standard MTT colorimetric assay^44^. Briefly, 100 *μ*L of 5 × 10^4^ cells/mL in Dulbecco’s modified eagle’s medium (DMEM) supplemented with 10% FBS were plated into 96-wells flat bottom plate and incubated overnight at 37 °C in 5% CO_2_. Three different concentrations of test compound (1, 10 and 100 *µ*g/mL) were added to the plate in triplicates and incubated for 48 hrs. 50 *µ*L of 0.5 mg/mL MTT was added to each well and plate was then further incubated for 4 hours. MTT was aspirated and 100 *µ*L of DMSO was then added to each well. The extent of MTT reduction to formazan within cells was calculated by measuring the absorbance at 540 nm, using spectrophotometer (Spectra Max plus, Molecular Devices, CA, USA). The cytotoxic activity was recorded as concentration causing 50% growth inhibition (IC_50_) for 3T3 cells.

### Methodology of *in silico* study

The 3D structure of *α*-amylase (PDB ID: 1HNY) was obtained from Protein Data Bank. Water molecules were removed and the 3D protonation of the protein molecule was carried out. Energy of the protein molecule was minimized with the help of energy minimization algorithm implemented in MOE (Molecular Operating Environment) software and the minimized structure was used for docking. The 3D structures of ligands were built using builder tool in MOE (www.chemcomp.com). All the built structures were 3D protonated and were energy minimized. The 3D structure were saved in mdb file format as input file for docking.

## Electronic supplementary material

Supplementary Information

## References

[1] Sales PM, Souza PM, Simeoni LA, Magalhães PO, Silveira D (2012). *α*-Amylase inhibitors: A review of raw material and isolated compounds from plant source. J. Pharm. Pharmaceut. Sci..

[2] Saltiel AR, Kahn CR (2001). Insulin signalling and the regulation of glucose and lipid metabolism. Nature.

[3] Funke I, Melzing MF (2006). Traditionally used plants in diabetes therapy-phytotherapeutics as inhibitors of *α*-amylase activity. Rev. Bras. Farmacogn..

[4] Inzucchi SE (2002). Oral antihyperglycemic therapy for type 2 diabetes. JAMA.

[5] Goke B, Herrmann-Rinke C (1998). The evolving role of *α*-glucosidase inhibitors. Diabetes/Metab Res..

[6] He L (1998). *α*-Glucosidase inhibitors as agents in the treatment of diabetes. Diabetes Rev..

[7] Whitcomb DC, Lowe ME (2007). Human pancreatic digestive enzymes. Dig. Dis. Sci..

[8] Kandra L (2003). *α*-Amylases of medical and industrial importance. J. Mol. Struct..

[9] Laar, F. A. *et al*. *α*-Glucosidase inhibitors for type 2 diabetes mellitus (Cochrane Review). The Cochrane Library, (2008).

[10] Cheng AYY, Fantus IG (2005). Oral antihyperglycemic therapy for type 2 diabetes Mellitus. Can. Med. Assoc. J..

[11] Tarling CA (2008). The search for novel human pancreatic *α*‐amylase inhibitors: high‐throughput screening of terrestrial and marine natural product extracts. ChemBioChem.

[12] Murao S, Goto A, Matsui Y, Ohyama K (1980). New proteinous inhibitor (Haim) of animal *α*-amylase from *Streptomyces griseosporeus* YM-25. Agric. Biol. Chem..

[13] Vértesy L, Oeding V, Bender R, Zepf K, Nesemann G (1984). Tendamistat (HOE 467), a tight‐binding *α*‐amylase inhibitor from *Streptomyces tendae* 4158. Eur. J. Biochem..

[14] Alexandra G, Maria JM, Jorge G, Eugenio U, Fernanda B (2014). Chromone: a valid scaffold in medicinal chemistry. Chem. Rev..

[15] Khan KM (2009). Schiff bases of 3-formylchromones as antibacterial, antifungal, and phytotoxic agents. Lett. Drug Des. Discov..

[16] Khan KM, Ambreen N, Hussain S, Perveen S, Choudhary MI (2009). Schiff bases of 3-formylchromone as thymidine phosphorylase inhibitors. Bioorg. Med. Chem..

[17] Jones QRD, Warford J, Rupasinghe HPV, Robertson GS (2012). Target-based selection of flavonoids for neurodegenerative disorders. Trends Pharmacol. Sci..

[18] Chen G (2008). A new chromone glycoside from *Rhododendron spinuliferum*. Arch. Pharm. Res..

[19] Conrad JR (2009). Flavonoid glucuronides and a chromone from the aquatic macrophyte Stratiotes aloides. J. Nat. Prod..

[20] Kashyap SJ (2012). Thiazoles: having diverse biological activities. Med. Chem. Res..

[21] Siddiqui N, Arshad MF, Ahsan W, Alam MS (2009). Thiazoles: a valuable insight into the recent advances and biological activities. Int. J. Pharm. Sci. Drug Res..

[22] Terzidis MA, Stephanidou-Stephanatou J, Tsoleridis CA (2009). Engaging a thiazole-DMAD zwitterion in novel one-pot multicomponent reactions involving chromones. Expeditious synthesis of thiazolo-and chromenothiazolopyridines. Tetrahedron Lett..

[23] Terzidis MA (2010). Expeditious one-pot synthesis of highly substituted thiazolo [3, 2-a] pyridines involving chromones. Tetrahedron.

[24] Khilya VP, Kupchevskaya IP, Kazakov AL, Tkachuk TM, Golubushina GM (1982). Chemistry of heteroanalogs of isoflavones. Reaction of thiazole analogs of isoflavones with nucleophilic and electrophilic reagents, Chem. Heterocycl. Compd..

[25] Karale BK, Takate SJ, Salve SP, Zaware BH, Jadhav SS (2015). Synthesis and biological screening of some novel thiazolyl chromones and pyrazoles. Indian J. Chem..

[26] Arshad T (2016). Syntheses, *in vitro* evaluation and molecular docking studies of 5-bromo-2-aryl benzimidazoles as *α*-glucosidase inhibitors. Med. Chem. Res..

[27] Khan KM (2016). Synthesis, *in vitro α*-glucosidase inhibitory activity and molecular docking studies of new thiazole derivatives. Bioorg. Chem..

[28] Javaid K (2015). 2-Arylquinazolin-4(3*H*)-ones: A new class of *α*-glucosidase inhibitors. Bioorg. Chem..

[29] Bano B (2015). Antiglycation activity of quinoline derivatives- A new therapeutic class for the management of type 2 diabetes complications. Med. Chem..

[30] Abbasi S (2014). Benzothiazole derivatives: Novel inhibitors of methylglyoxal mediated glycation of protein *in vitro*. Med. Chem..

[31] Khan KM (2013). Oxindole derivatives: Synthesis and antiglycation activity. Med. Chem..

[32] Salar U (2016). Syntheses of new 3-thiazolyl coumarin derivative, *in vitro α*-glucosidase inhibitory activity, and modeling studies. Eur. J. Med. Chem..

[33] Jadhav RK, Nikumbh AB, Karale BK (2015). Synthesis and screening of fluoro substituted pyrazolyl benzoxazoles. Orient J. Chem..

[34] Liu, C. *et al*. Faming Zhuanli Shenqing, 2015, CN 105017197, A CAN163:666419, CAPLUS.

[35] Nastasa C (2013). Synthesis and antimicrobial activity of some novel 2-aryliden-hydrazonethiazoles. Farmacia (Bucharest. Romania).

[36] Coutinho DLM, Fernandes PS (1992). Synthesis and evaluation of potential pharmacophores derived from 4-oxo-4*H*-1-benzopyran-3-carboxyldehyde. Indian J. Chem. (Section B) Organic Chemistry Including Medicinal Chemistry.

[37] Kwon YI, Apostolidis E, Shetty K (2008). *In vitro* studies of eggplant (*Solanum melongena*) phenolics as inhibitors of key enzymes relevant for type 2 diabetes and hypertension. Bioresour Technol..

[38] Loh SP, Hadira O (2011). *In vitro* inhibitory potential of selected Malaysian plants against key enzymes involved in hyperglycemia and hypertension. Malays. J. Nutr..

[39] Ushasri R, Anusha R (2015). *In vitro* anti-diabetic activity of ethanolic and acetone extracts of endophytic fungi *Syncephalastrum racemosum* isolated from the seaweed Gracilaria corticata by alpha-amylase inhibition assay method. Int. J. Curr. Microbiol. App. Sci..

[40] Suthakaran R (2007). Synthesis, antiinflammatory, antioxidant and antibacterial activities of 7-methoxy benzofuran pyrazoline derivatives. Asian J. Chem..

[41] Sridevi CH, Balaji K, Naidu A, Sudhakaran R (2009). Antioxidant, anti-inflammatory and histaminic activities of some phenyl pyrazolo benzothiazolo quinoxaline derivatives, Int. J. Chem. Sci..

[42] Sridevi CH, Balaji K, Naidu A (2011). Synthesis and pharmacological evaluation of some phenylpyrazolo indoquinoxaline derivatives. J. Chem..

[43] Chigurupati S (2016). Identification of novel acetylcholinesterase inhibitors: Indolopyrazoline derivatives and molecular docking studies. Bioorg. Chem..

[44] Jabeen A (2016). Anti-TNF-*α* and antiarthritic effect of patuletin: A rare flavonoid from *Tagetes patula*. Int. immunopharm..

[45] Arshad T (2017). 5-Bromo-2-aryl benzimidazole derivatives as non-cytotoxic potential dual inhibitors of *α*-glucosidase and urease enzymes. Bioorg. Chem..

