# Supplementary material for: New Hybrid Hydrazinyl Thiazole Substituted Chromones: As Potential α-Amylase Inhibitors and Radical (DPPH & ABTS) Scavengers
Source: Sci Rep. 2017 Dec 5;7:16980. doi: 10.1038/s41598-017-17261-w (PMC5717224; doi:10.1038/s41598-017-17261-w)
Supplement: Supplementary file 1 — Supplementary Information [file 41598_2017_17261_MOESM1_ESM.pdf]

New Hybrid Hydrazinyl Thiazole Substituted Chromones: As Potential α-Amylase Inhibitors and Radical (DPPH & ABTS) Scavengers

Uzma Salar,[a] Khalid Mohammed Khan,[*a] Sridevi Chigurupati,[b] Muhammad Taha,[c] Abdul Wadood,[d] Shantini Vijayabalan,[b] Mehreen Ghufran,[d] Shahnaz Perveen,[e]

[a]*H. E. J. Research Institute of Chemistry, International Center for Chemical and Biological Sciences, University of Karachi, Karachi-75270, Pakistan*
[b]*Department of Pharmaceutical chemistry, Faculty of Pharmacy, AIMST University, Semeling, 08100 Bedong, Kedah, Malaysia*
[c]*Department of Clinical Pharmacy, Institute for Research and Medical Consultations (IRMC), University of Dammam, Dammam 31441, Saudi Arabia*
[d]*Department of Biochemistry, Computational Medicinal Chemistry Laboratory, UCSS, Abdul Wali Khan University, Mardan, Pakistan*
[e]*PCSIR Laboratories Complex, Karachi, Shahrah-e-Dr. SalimuzzamanSiddiqui, Karachi-75280, Pakistan*

[*]*Corresponding Author*: khalid.khan@iccs.edu; drkhalidhej@gmail.com; Tel. 00922134824910; Fax. 00922134819018

UZMA/DR,KHALID/US-IV-74/
ICCBS,U.O.K/

Compound 1

AVANCE 400
LAB NO 117

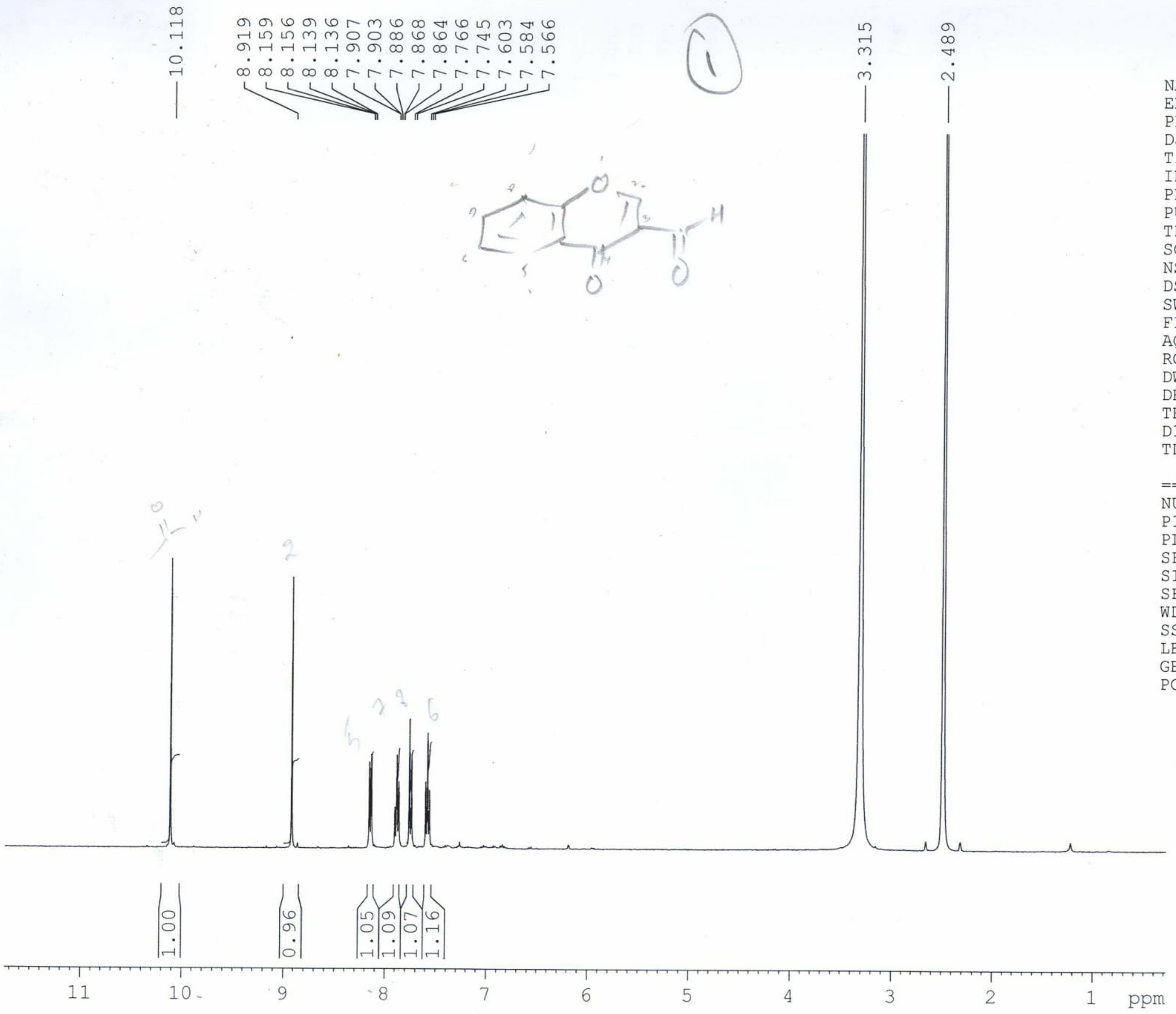

NAME	aug26-14
EXPNO	6
PROCNO	1
Date_	20140826
Time	11.43
INSTRUM	spect
PROBHD	5 mm DUL 13C-1
PULPROG	zg30
TD	32768
SOLVENT	DMSO
NS	32
DS	0
SWH	8012.820 Hz
FIDRES	0.244532 Hz
AQ	2.0447731 sec
RG	2048
DW	62.400 usec
DE	6.50 usec
TE	300.0 K
D1	2.00000000 sec
TD0	1

======== CHANNEL f1 ========

NUC1	1H
P1	10.20 usec
PL1	0.00 dB
SFO1	400.1332010 MHz
SI	16384
SF	400.1300071 MHz
WDW	EM
SSB	0
LB	0.30 Hz
GB	0
PC	1.00

[illegible]

File: US-IV-75
Sample: UZMA SALAR / DR. KHALID
Instrument: JEOL JMS 600-H

Date Run: 08-22-2014 (Time Run: 11:44:18)

Ionization mode: EI+

Compound 2

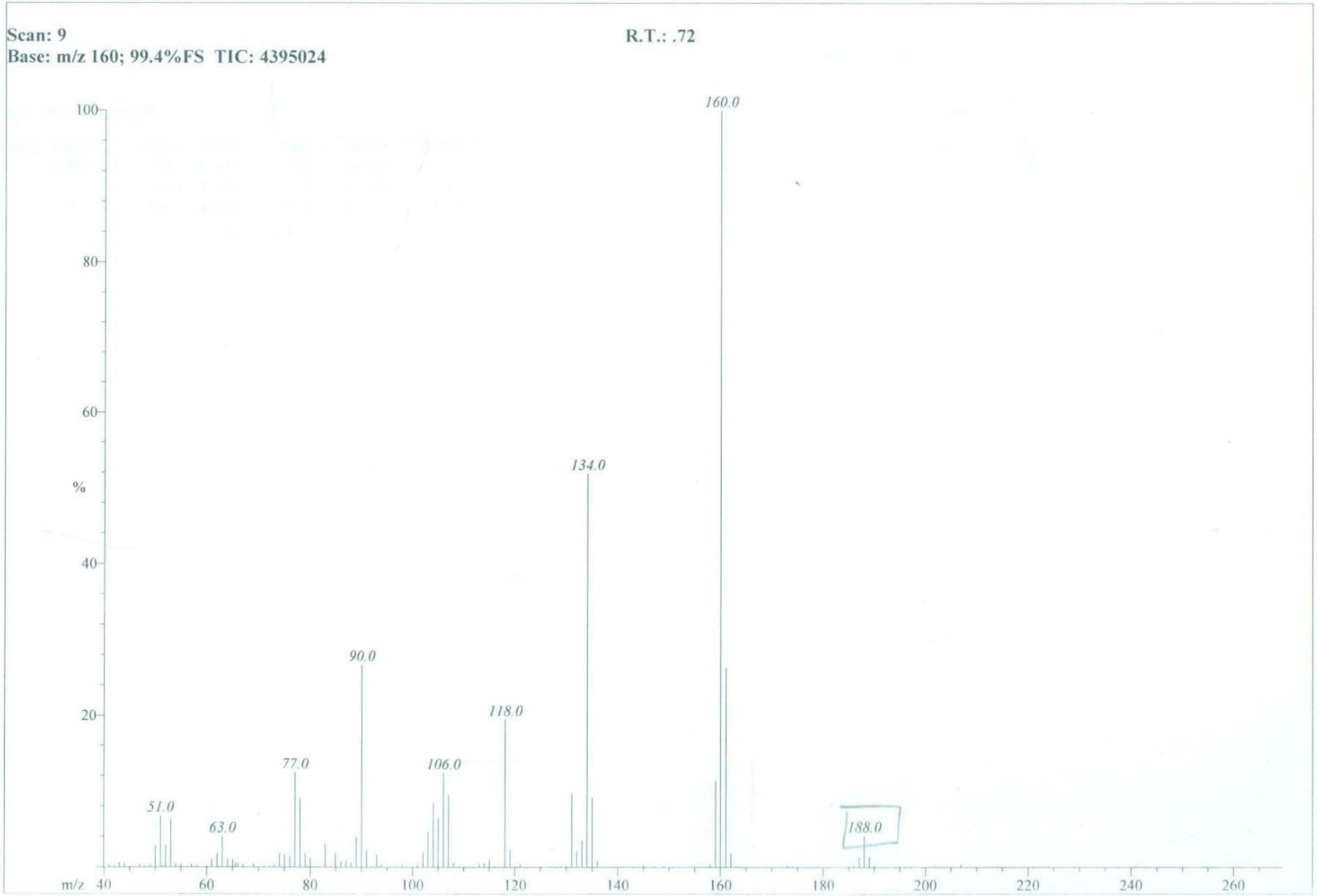

UZMA/DR,KHALID/US-IV-75/
ICCBS,U.O.K/

Compound 2

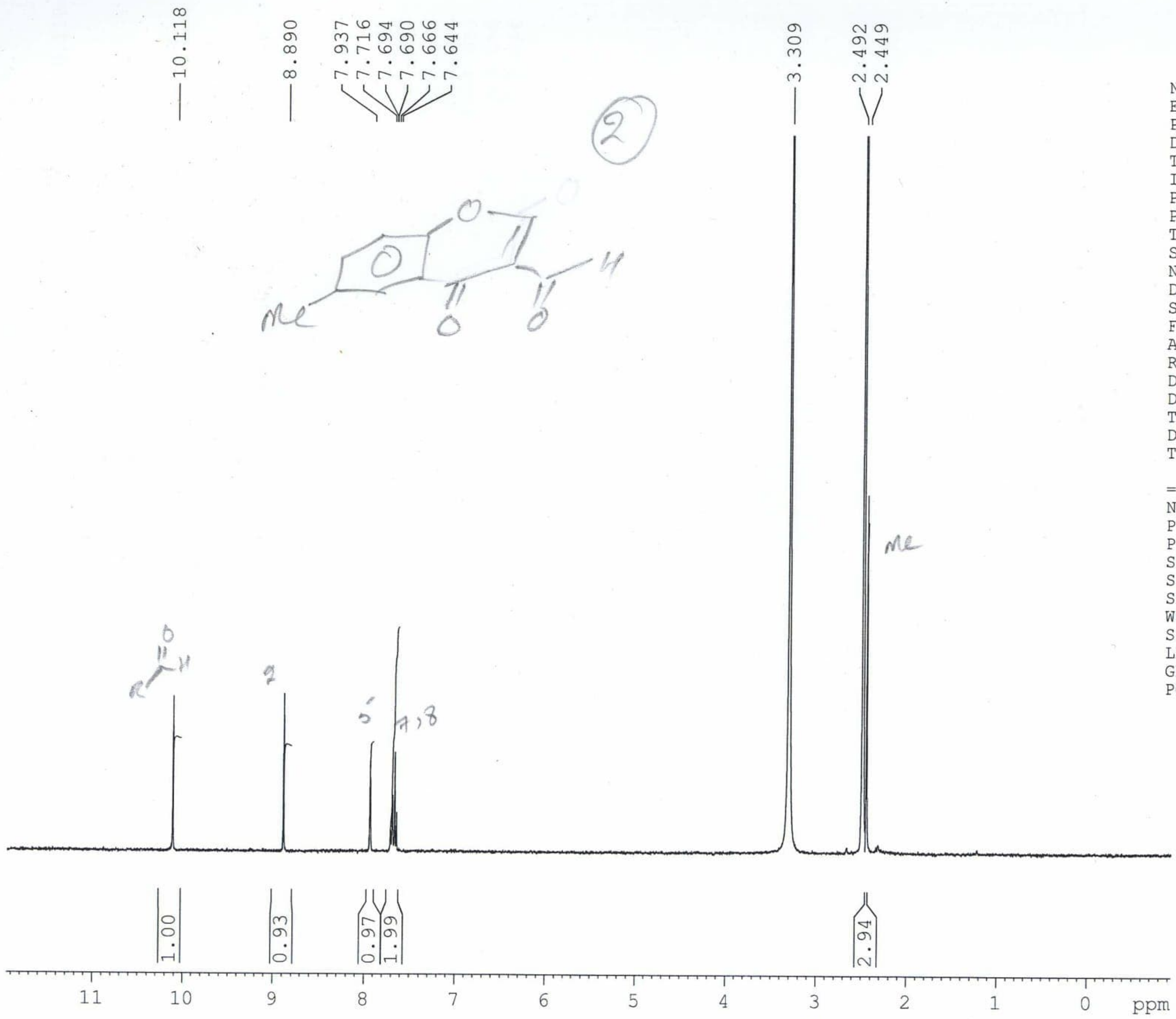

AVANCE 400
LAB NO 117

NAME	sep05-14
EXPNO	2
PROCNO	1
Date_	20140905
Time	9.57
INSTRUM	spect
PROBHD	5 mm DUL 13C-1
PULPROG	zg30
TD	32768
SOLVENT	DMSO
NS	64
DS	0
SWH	8012.820 Hz
FIDRES	0.244532 Hz
AQ	2.0447731 sec
RG	2048
DW	62.400 usec
DE	6.50 usec
TE	300.0 K
D1	2.00000000 sec
TD0	1

======== CHANNEL f1 ========

NUC1	1H
P1	10.20 usec
PL1	0.00 dB
SFO1	400.1332010 MHz
SI	16384
SF	400.1300064 MHz
WDW	EM
SSB	0
LB	0.30 Hz
GB	0
PC	1.00

File: US-V-7 **Date Run: 09-11-2014 (Time Run: 09:19:18)**
Sample: UZMA SALAR /DR. KHALID
Instrument: JEOL JMS 600-H
Inlet: My Inlet **Ionization mode: EI+**

Compound 3

Scan: 30 R.T.: 2.58
Base: m/z 172; 4.6%FS TIC: 323351 #Ions: 59

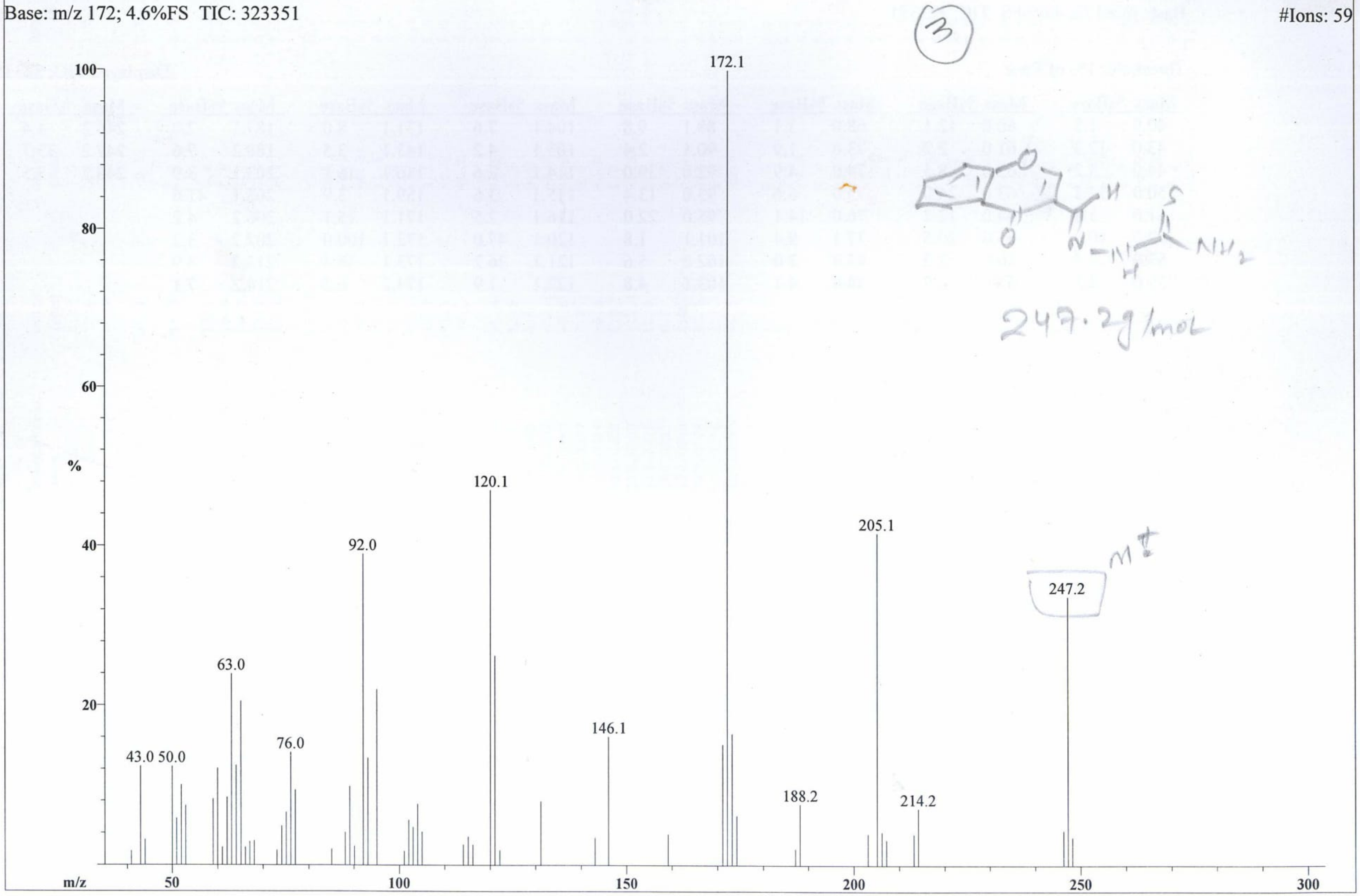

UZMA/DR,KHALID/US-V-7/
ICCBS,U.O.K/

Compound 3

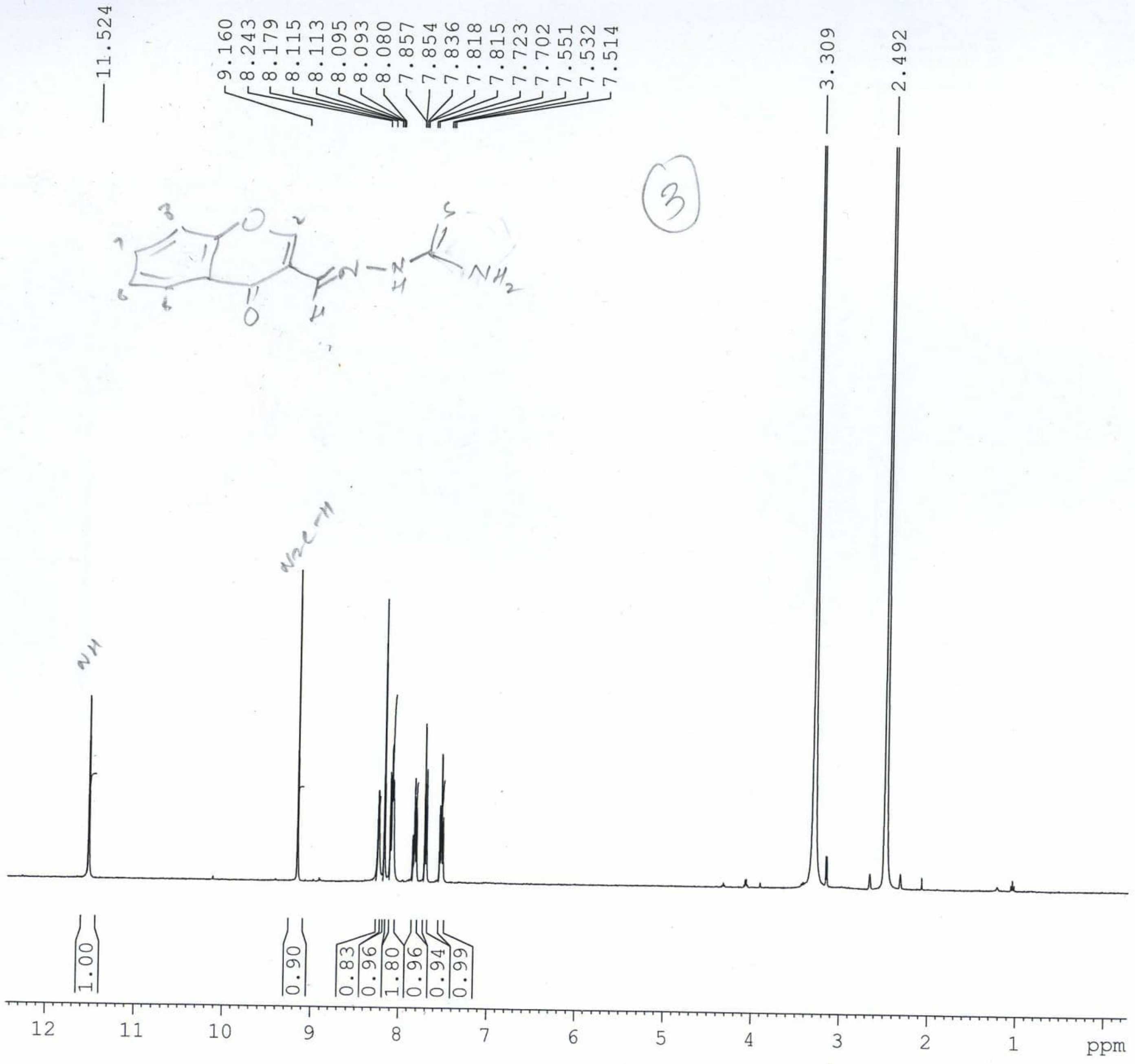

AVANCE 400
LAB NO 117

NAME	nov07-14
EXPNO	6
PROCNO	1
Date_	20141107
Time	9.15
INSTRUM	spect
PROBHD	5 mm DUL 13C-1
PULPROG	zg30
TD	32768
SOLVENT	DMSO
NS	64
DS	0
SWH	8012.820 Hz
FIDRES	0.244532 Hz
AQ	2.0447731 sec
RG	362
DW	62.400 usec
DE	6.50 usec
TE	300.0 K
D1	2.00000000 sec
TD0	1

======== CHANNEL f1 ========

NUC1	1H
P1	10.20 usec
PL1	0.00 dB
SFO1	400.1332010 MHz
SI	16384
SF	400.1300064 MHz
WDW	EM
SSB	0
LB	0.30 Hz
GB	0
PC	1.00

Compound 4

File: US-V-8
Sample: UZMA SALAR /DR. KHALID
Instrument: JEOL JMS 600-H
Inlet: My Inlet

Date Run: 09-11-2014 (Time Run: 09:27:21)

Ionization mode: EI+

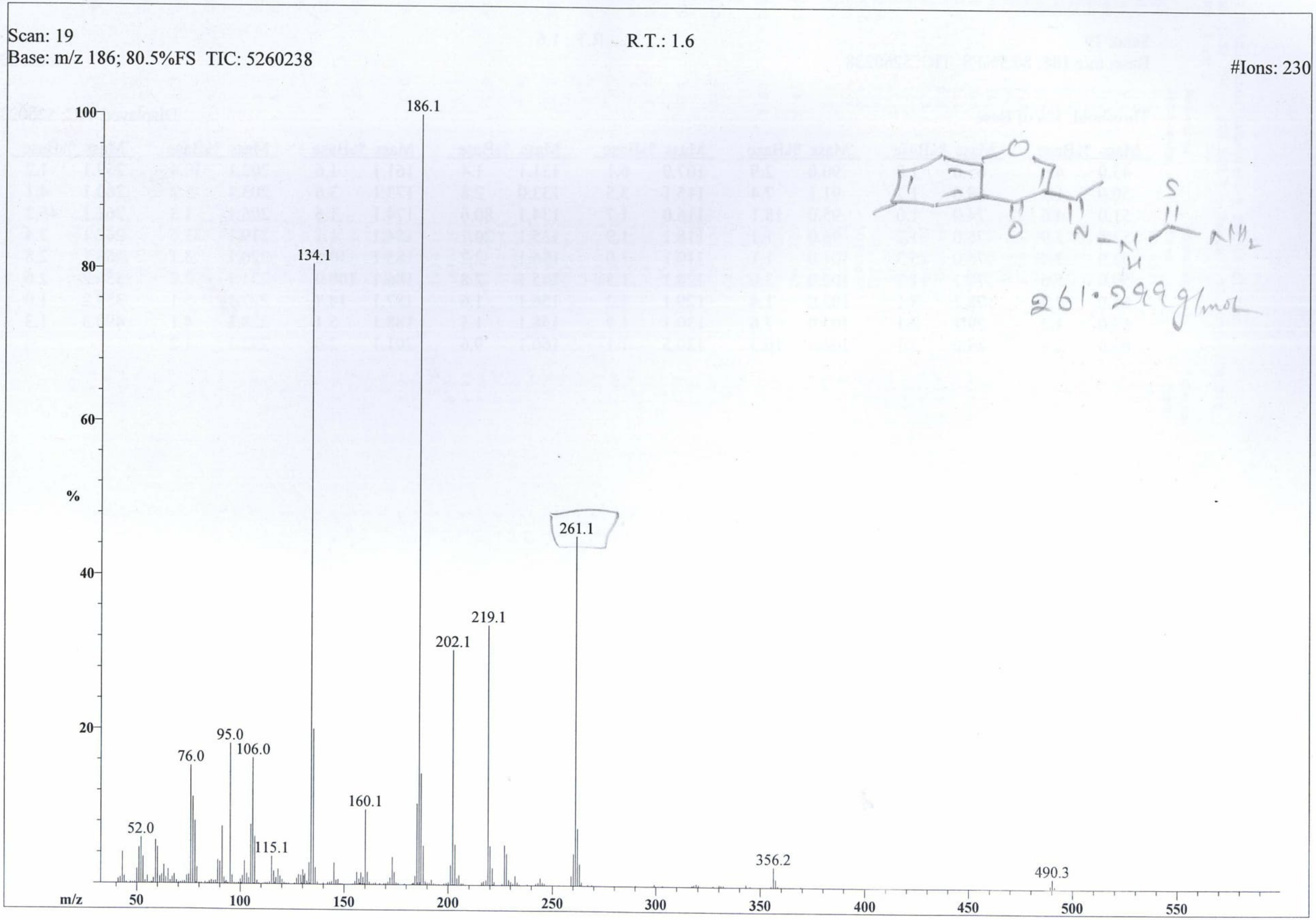

Compound 4

UZMA/DR,KHALID/US-V-8/
ICCBS,U.O.K/

AVANCE 400
LAB NO 117

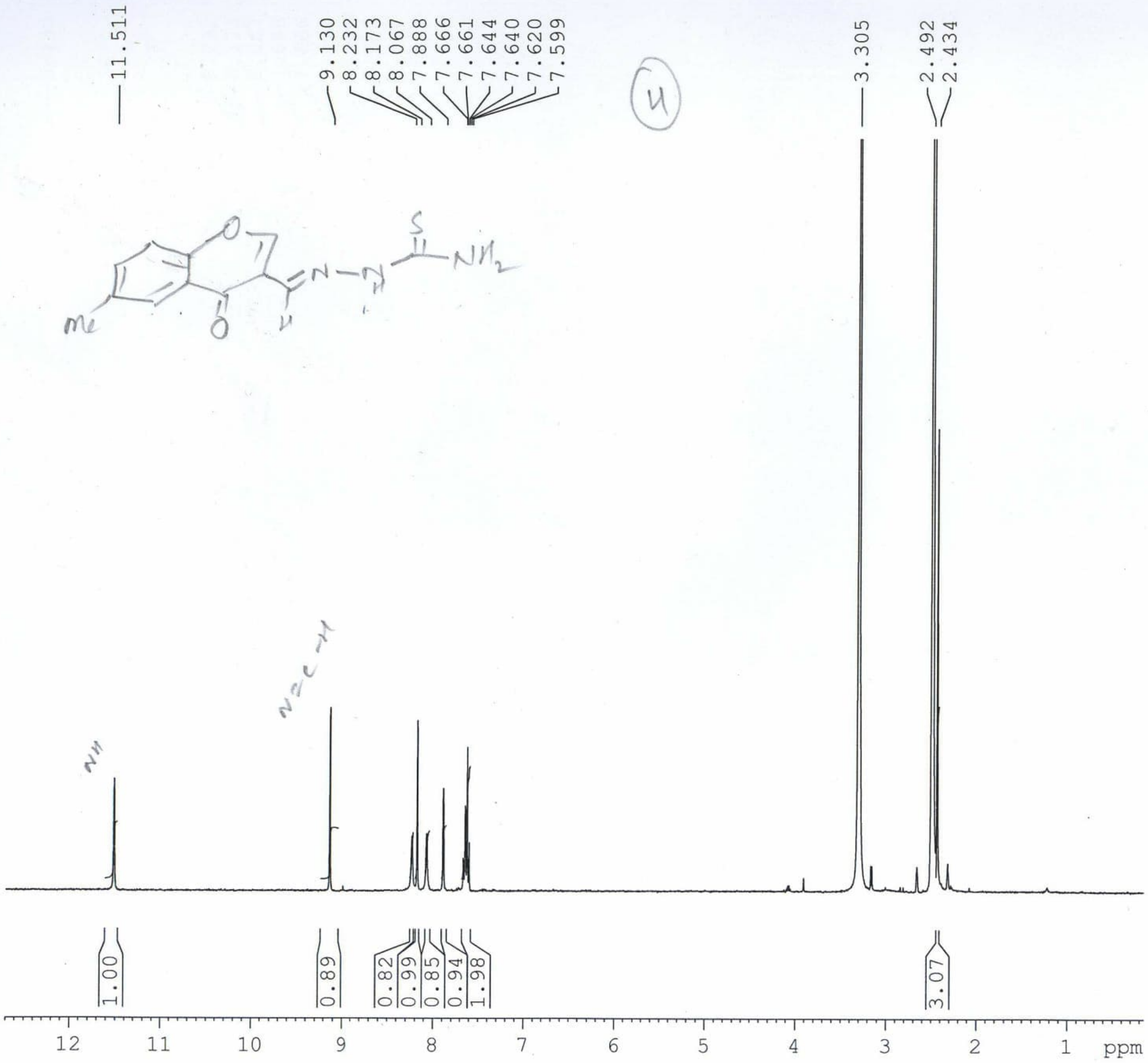

NAME	nov07-14
EXPNO	7
PROCNO	1
Date_	20141107
Time	9.24
INSTRUM	spect
PROBHD	5 mm DUL 13C-1
PULPROG	zg30
TD	32768
SOLVENT	DMSO
NS	64
DS	0
SWH	8012.820 Hz
FIDRES	0.244532 Hz
AQ	2.0447731 sec
RG	362
DW	62.400 usec
DE	6.50 usec
TE	300.0 K
D1	2.00000000 sec
TD0	1

======== CHANNEL f1 ========

NUC1	1H
P1	10.20 usec
PL1	0.00 dB
SFO1	400.1332010 MHz
SI	16384
SF	400.1300064 MHz
WDW	EM
SSB	0
LB	0.30 Hz
GB	0
PC	1.00

File: US-IV-80
Sample: UZMA SALAR /DR. KHALID
Instrument: JEOL JMS 600-H
Inlet: My Inlet

Date Run: 09-10-2014 (Time Run: 15:30:30)

Ionization mode: EI+

Compound 5

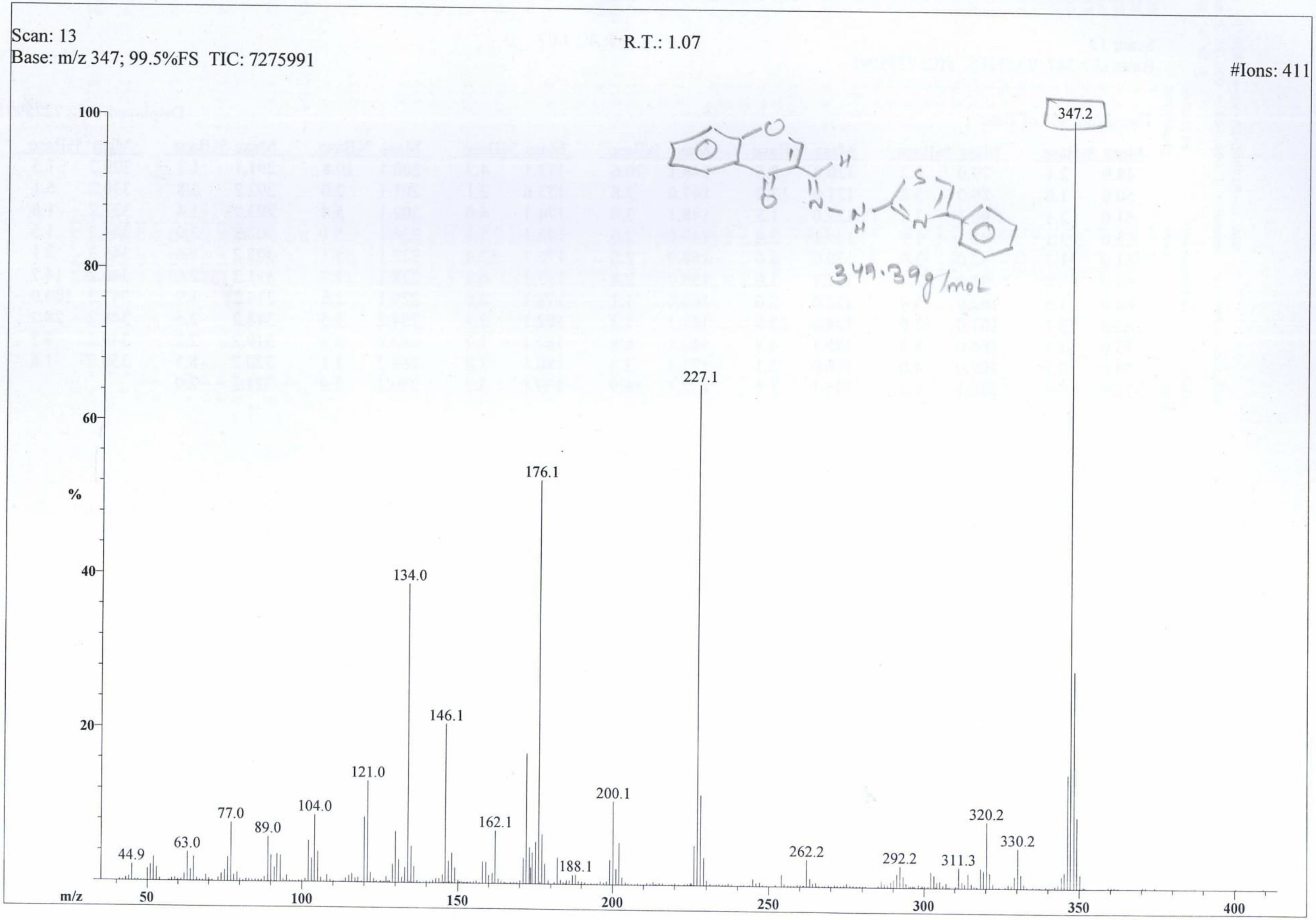

UZMA/DR,KHALID/US-IV-80/
ICCBS,U.O.K/

Compound 5

AVANCE 400
LAB NO 117

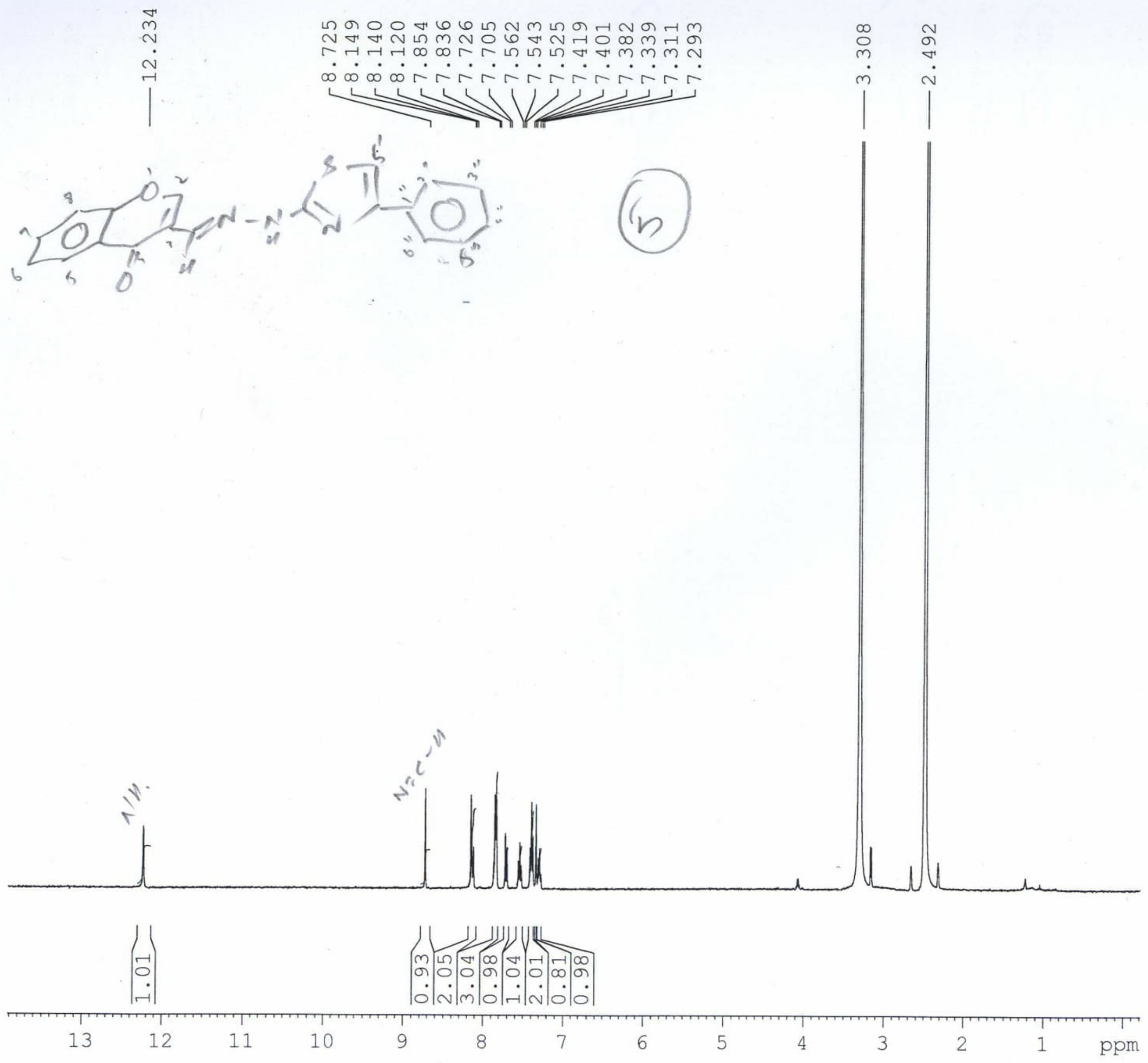

NAME	mar02-15	
EXPNO	10	
PROCNO	1	
Date_	20150302	
Time	10.37	
INSTRUM	spect	
PROBHD	5 mm DUL 13C-1	
PULPROG	zg30	
TD	32768	
SOLVENT	DMSO	
NS	64	
DS	0	
SWH	8012.820	Hz
FIDRES	0.244532	Hz
AQ	2.0447731	sec
RG	362	
DW	62.400	usec
DE	6.50	usec
TE	300.0	K
D1	2.00000000	sec
TD0	1	

======== CHANNEL f1 ========

NUC1	1H	
P1	10.20	usec
PL1	0.00	dB
SFO1	400.1332010	MHz
SI	16384	
SF	400.1300064	MHz
WDW	EM	
SSB	0	
LB	0.30	Hz
GB	0	
PC	1.00	

File: US-IV-81 **Date Run: 09-10-2014 (Time Run: 12:30:27)** Compound 6
Sample: UZMA SALAR /DR. KHALID
Instrument: JEOL JMS 600-H
Inlet: My Inlet **Ionization mode: EI+**

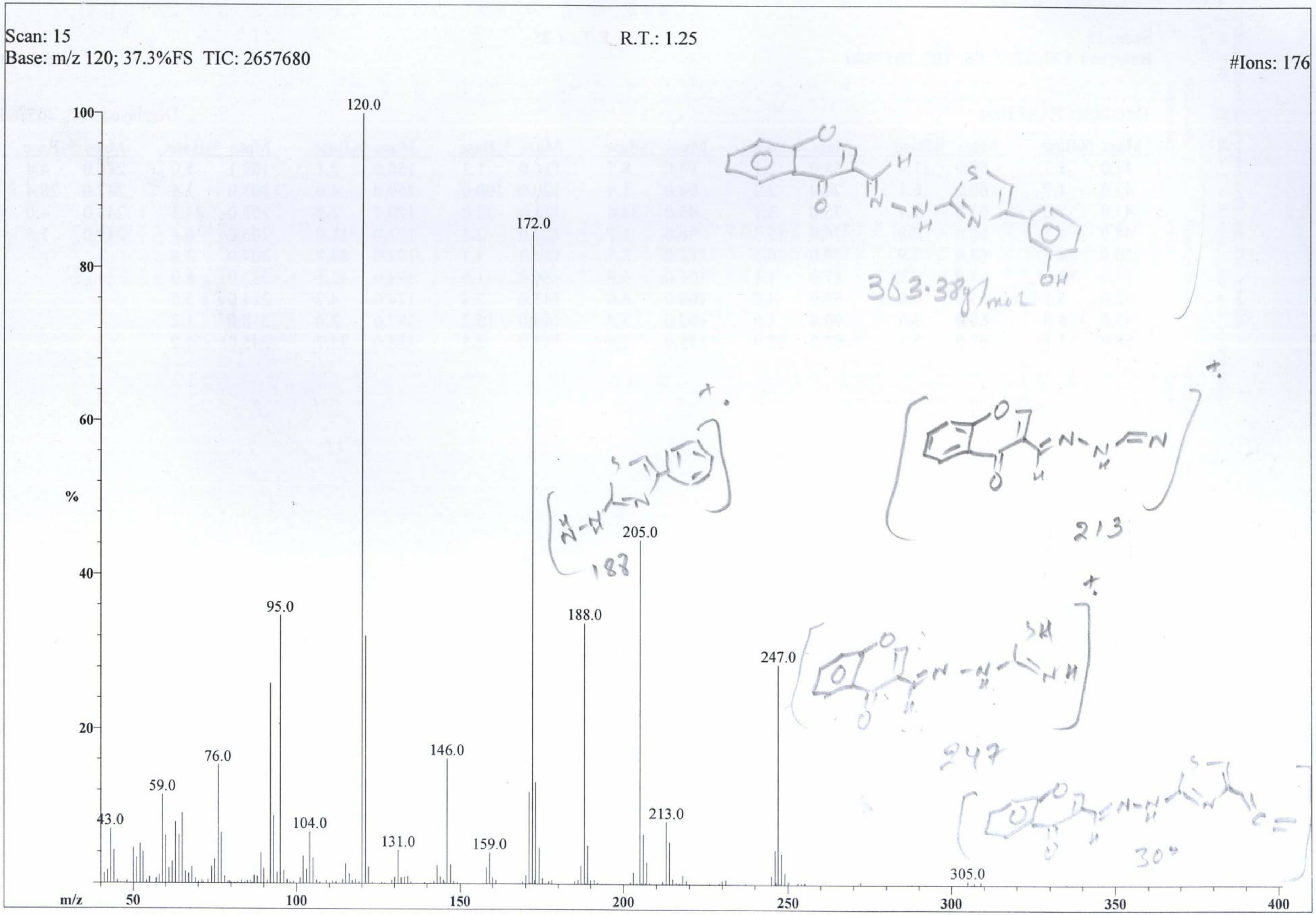

Compound 6

UZMA/DR,KHALID/US-IV-81/
ICCBS,U.O.K/

AVANCE 400
LAB NO 117

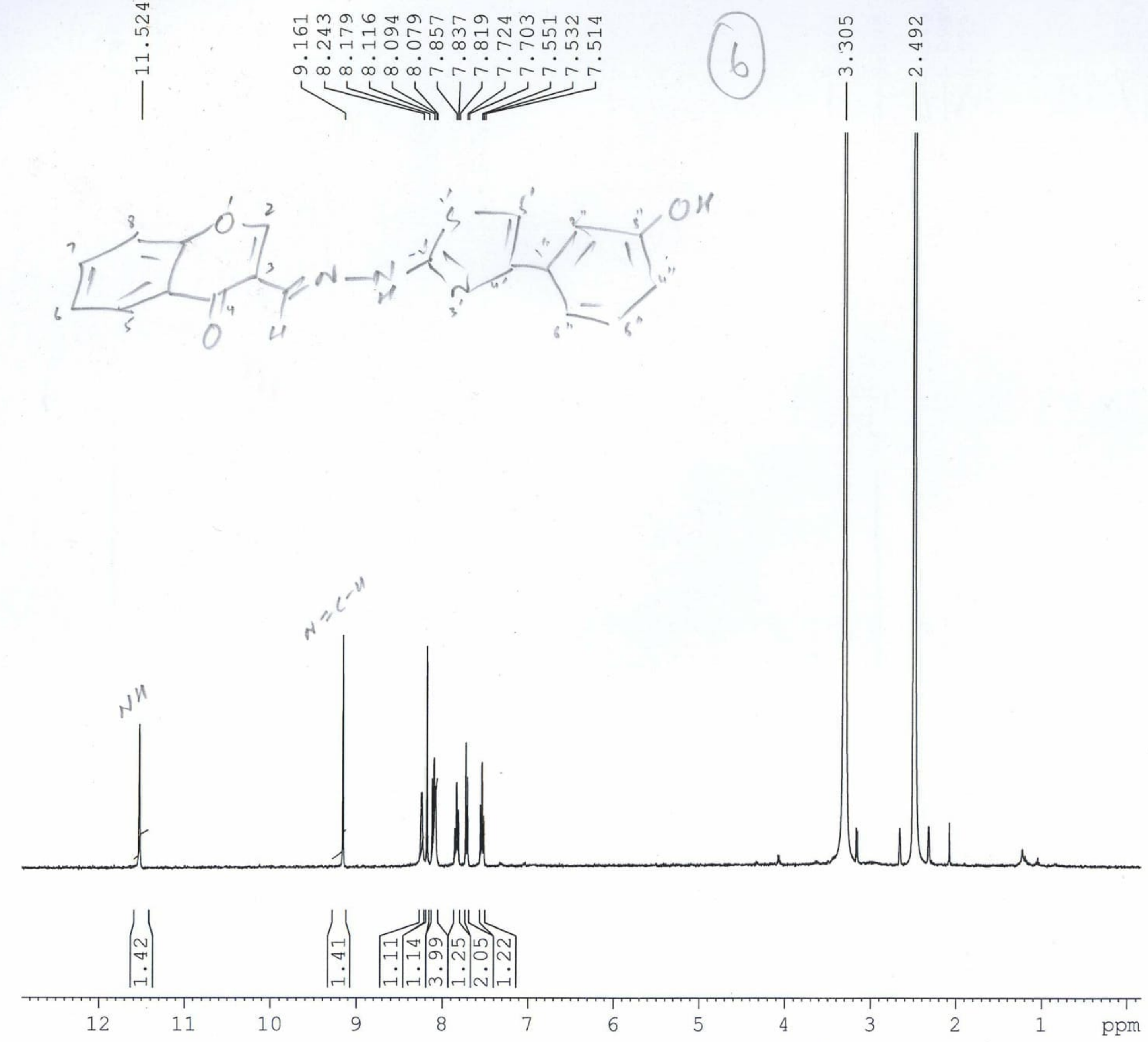

NAME	nov11-14	
EXPNO	3	
PROCNO	1	
Date_	20141111	
Time	8.51	
INSTRUM	spect	
PROBHD	5 mm DUL 13C-1	
PULPROG	zg30	
TD	32768	
SOLVENT	DMSO	
NS	64	
DS	0	
SWH	8012.820	Hz
FIDRES	0.244532	Hz
AQ	2.0447731	sec
RG	362	
DW	62.400	usec
DE	6.50	usec
TE	300.0	K
D1	2.00000000	sec
TD0	1	

======== CHANNEL f1 ========

NUC1	1H	
P1	10.20	usec
PL1	0.00	dB
SFO1	400.1332010	MHz
SI	16384	
SF	400.1300064	MHz
WDW	EM	
SSB	0	
LB	0.30	Hz
GB	0	
PC	1.00	

File: US-IV-82 **Date Run: 09-10-2014 (Time Run: 15:38:59)** Compound 7
Sample: UZMA SALAR /DR. KHALID
Instrument: JEOL JMS 600-H
Inlet: My Inlet **Ionization mode: EI+**

Scan: 15 R.T.: 1.25
Base: m/z 427; 41%FS TIC: 4735090 #Ions: 378

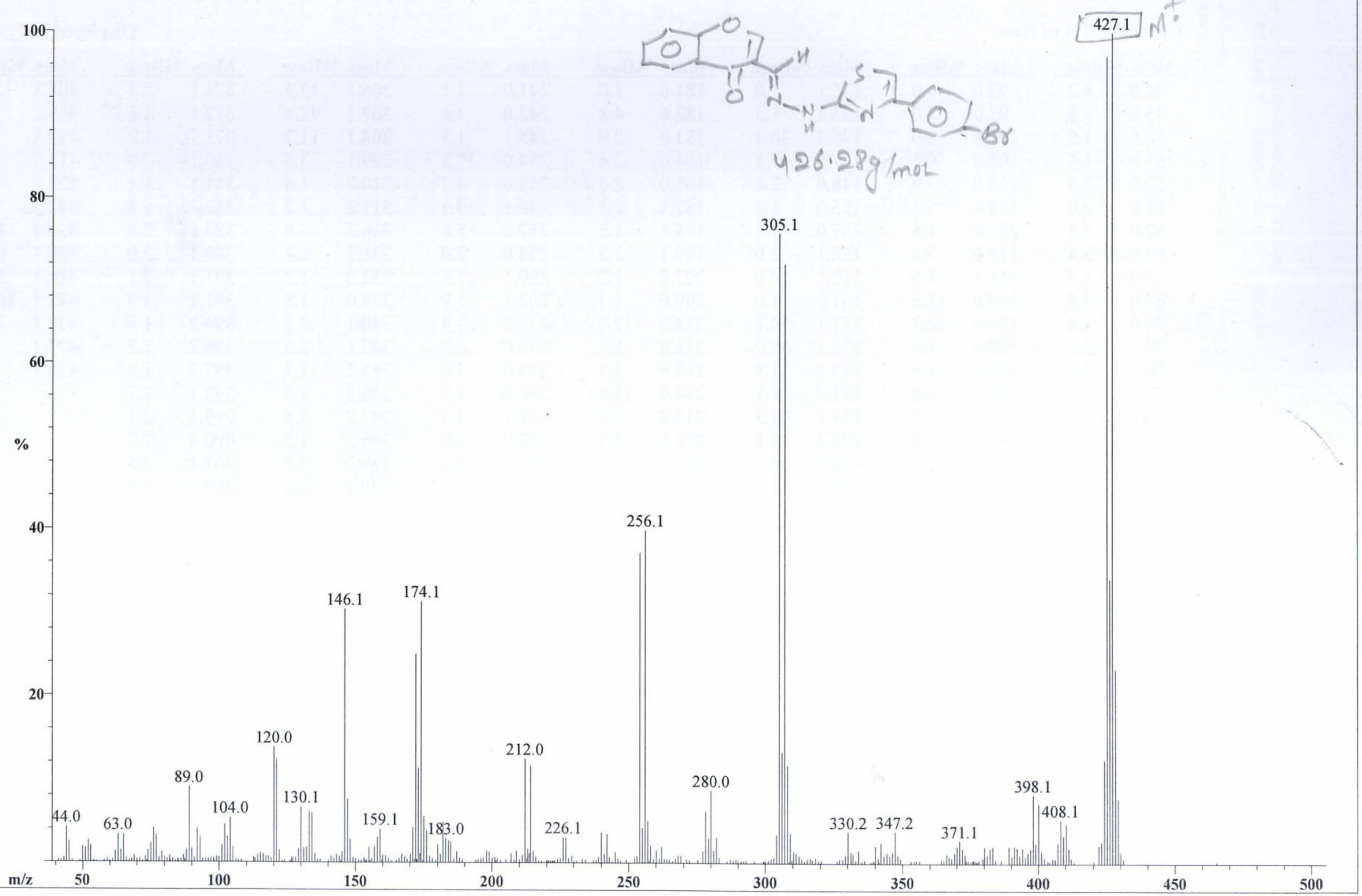

UZMA/DR,KHALID/US-IV-82/
ICCBS,U.O.K/

Compound 7

AVANCE 400
LAB NO 117

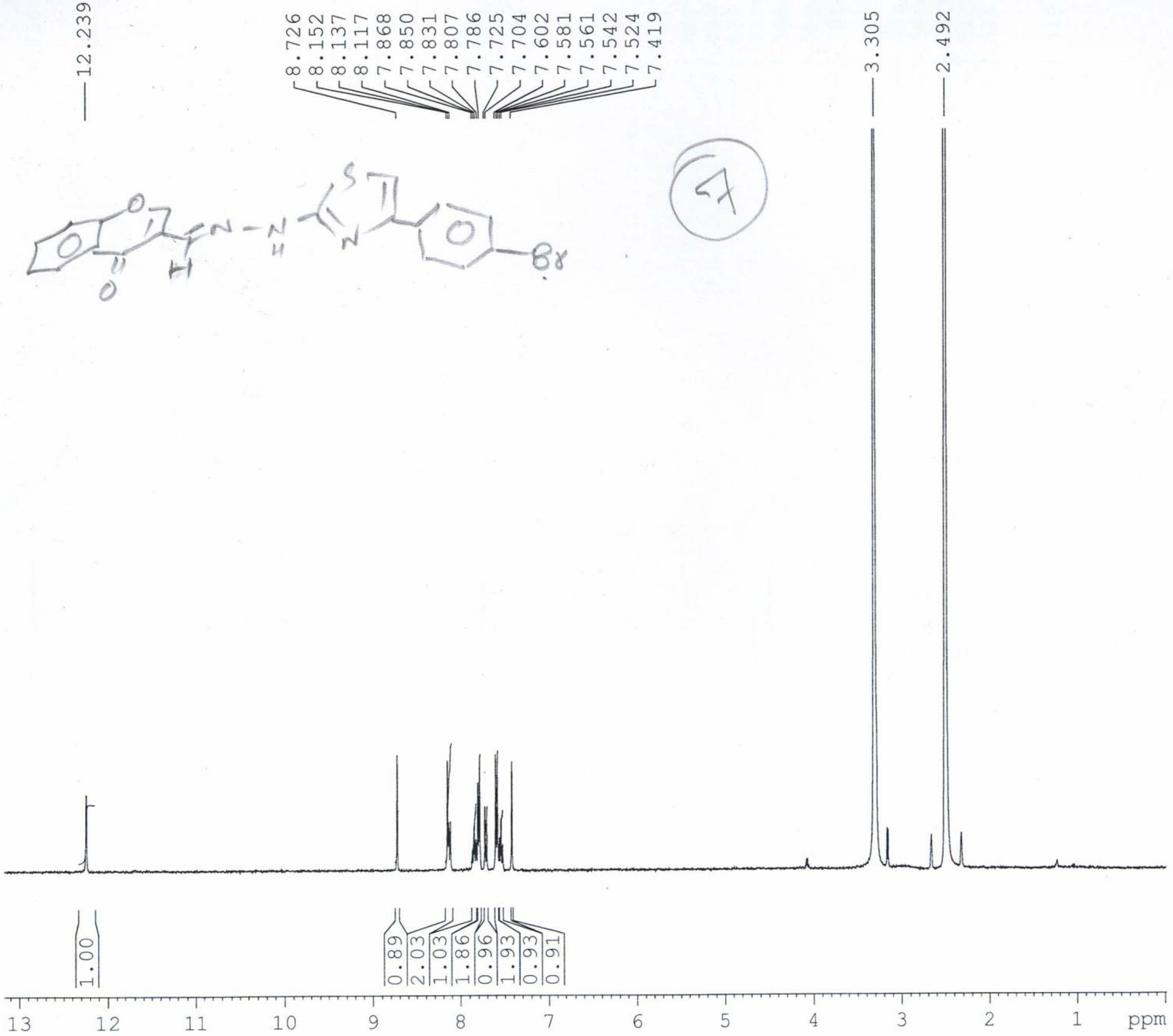

NAME	mar03-15
EXPNO	8
PROCNO	1
Date_	20150303
Time	9.40
INSTRUM	spect
PROBHD	5 mm DUL 13C-1
PULPROG	zg30
TD	32768
SOLVENT	DMSO
NS	64
DS	0
SWH	8012.820 Hz
FIDRES	0.244532 Hz
AQ	2.0447731 sec
RG	362
DW	62.400 usec
DE	6.50 usec
TE	300.0 K
D1	2.00000000 sec
TD0	1

======== CHANNEL f1 ========

NUC1	1H
P1	10.20 usec
PL1	0.00 dB
SFO1	400.1332010 MHz
SI	16384
SF	400.1300064 MHz
WDW	EM
SSB	0
LB	0.30 Hz
GB	0
PC	1.00

File: US-IV-85
Sample: UZMA SALAR /DR. KHALID
Instrument: JEOL JMS 600-H
Inlet: My Inlet

Date Run: 09-10-2014 (Time Run: 12:36:27)

Compound 8

Ionization mode: EI+

Scan: 16
Base: m/z 427; 84.7%FS TIC: 9570496

R.T.: 1.33

#Ions: 527

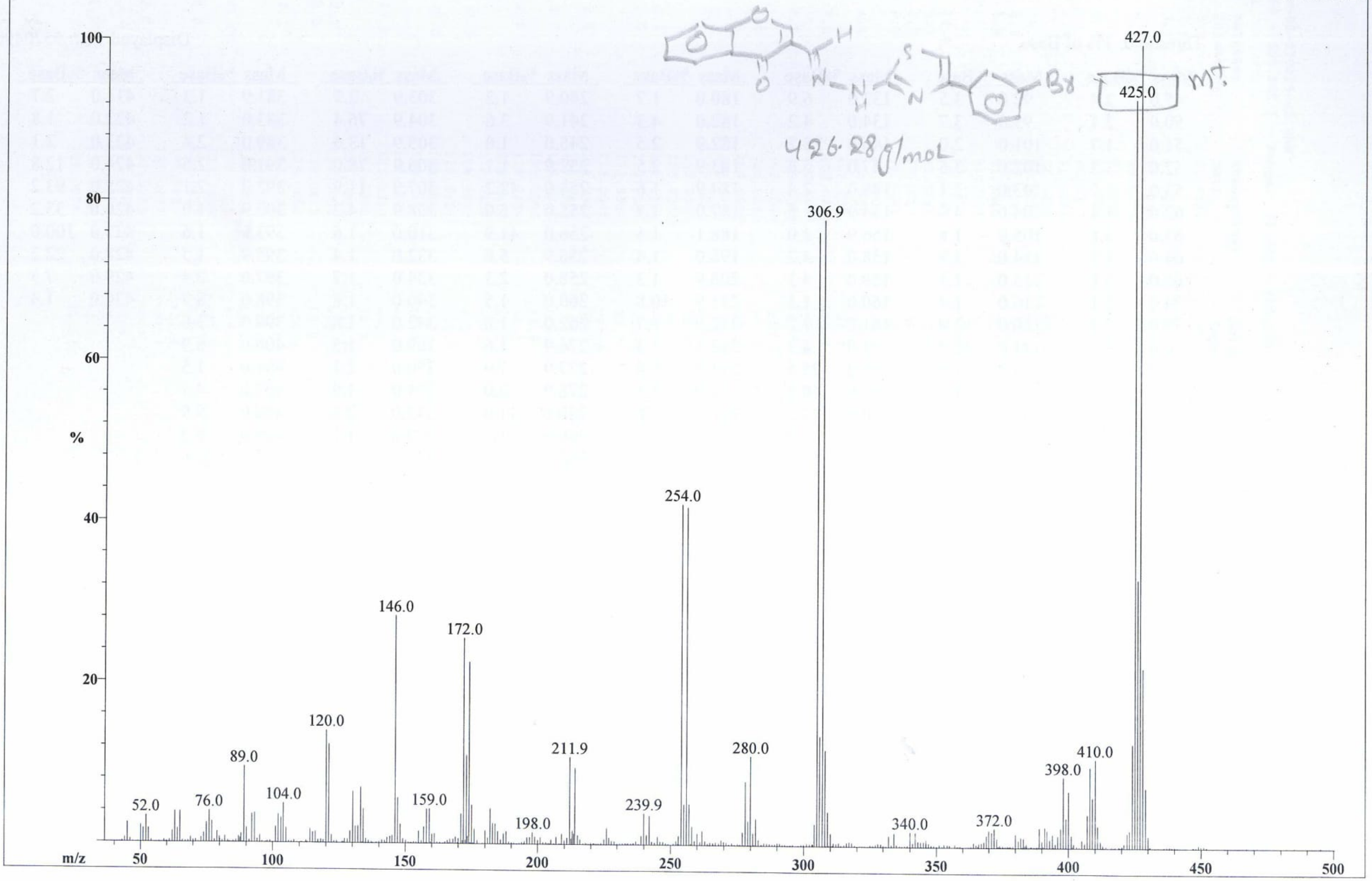

UZMA/DR,KHALID/US-IV-85/
ICCBS,U.O.K/

Compound 8

AVANCE 400
LAB NO 117

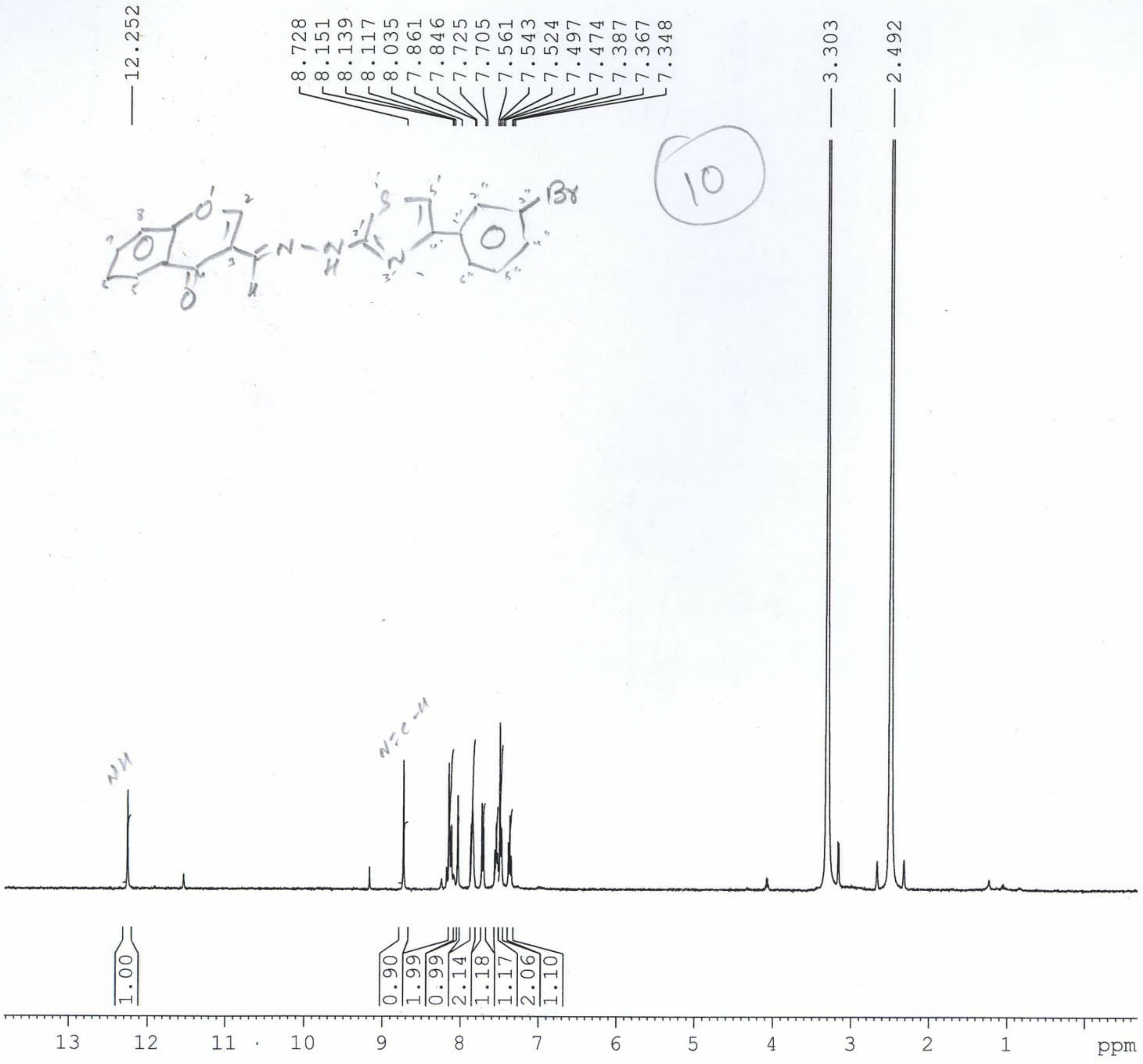

NAME	jan30-15	
EXPNO	6	
PROCNO	1	
Date_	20150130	
Time	9.01	
INSTRUM	spect	
PROBHD	5 mm DUL 13C-1	
PULPROG	zg30	
TD	32768	
SOLVENT	DMSO	
NS	64	
DS	0	
SWH	8012.820	Hz
FIDRES	0.244532	Hz
AQ	2.0447731	sec
RG	362	
DW	62.400	usec
DE	6.50	usec
TE	300.0	K
D1	2.00000000	sec
TD0	1	

======== CHANNEL f1 ========

NUC1	1H	
P1	10.20	usec
PL1	0.00	dB
SFO1	400.1332010	MHz
SI	16384	
SF	400.1300064	MHz
WDW	EM	
SSB	0	
LB	0.30	Hz
GB	0	
PC	1.00	

ICCBS-HEJ[LAB-104]
9/10/2014

File: US-IV-83
Sample: UZMA SALAR /DR. KHALID
Instrument: JEOL JMS 600-H
Inlet: My Inlet

Date Run: 09-10-2014 (Time Run: 12:11:36)

Compound 9

Ionization mode: EI+

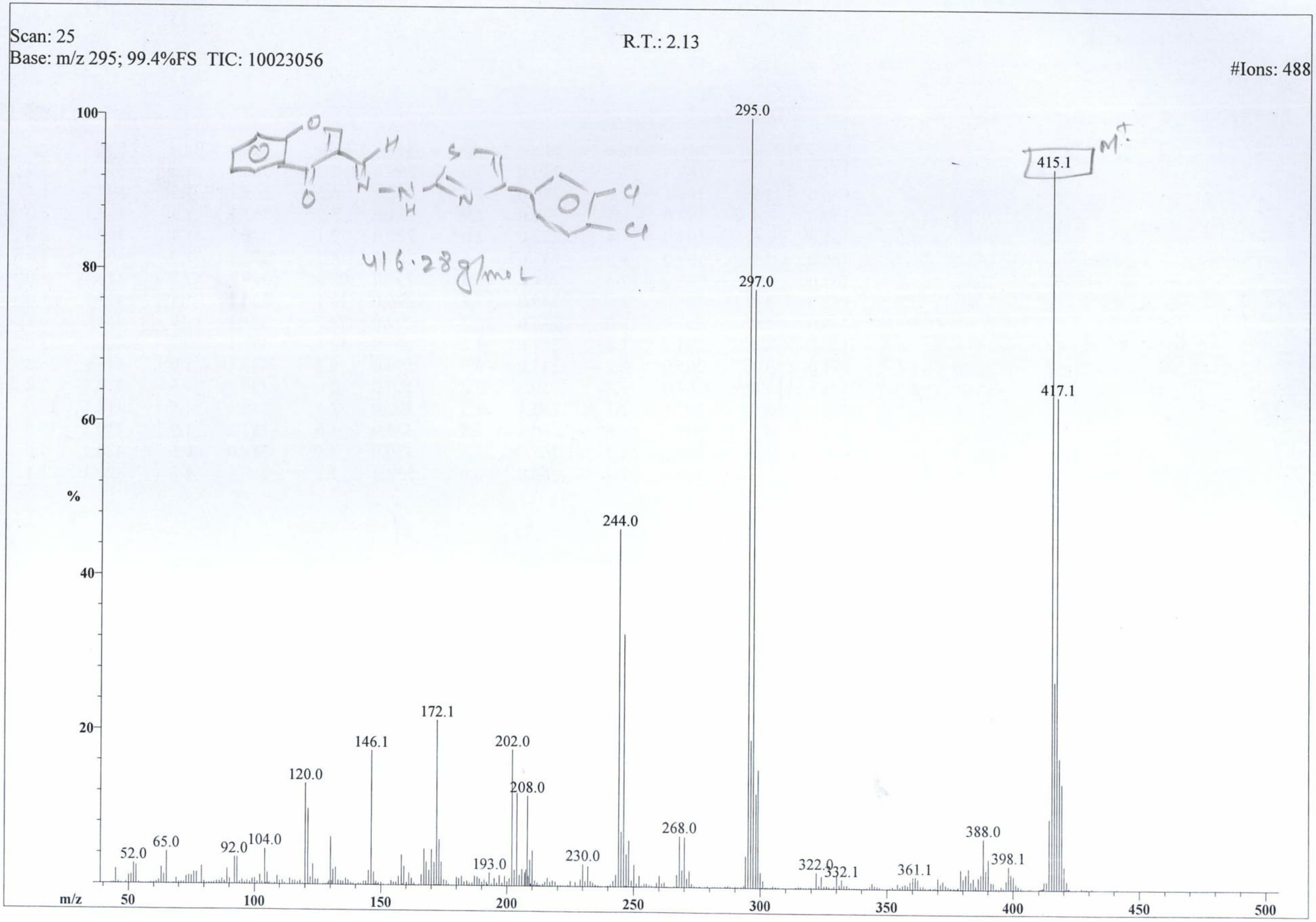

UZMA/DR,KHALID/US-IV-83/
ICCBS,U.O.K/

Compound 9

AVANCE 400
LAB NO 117

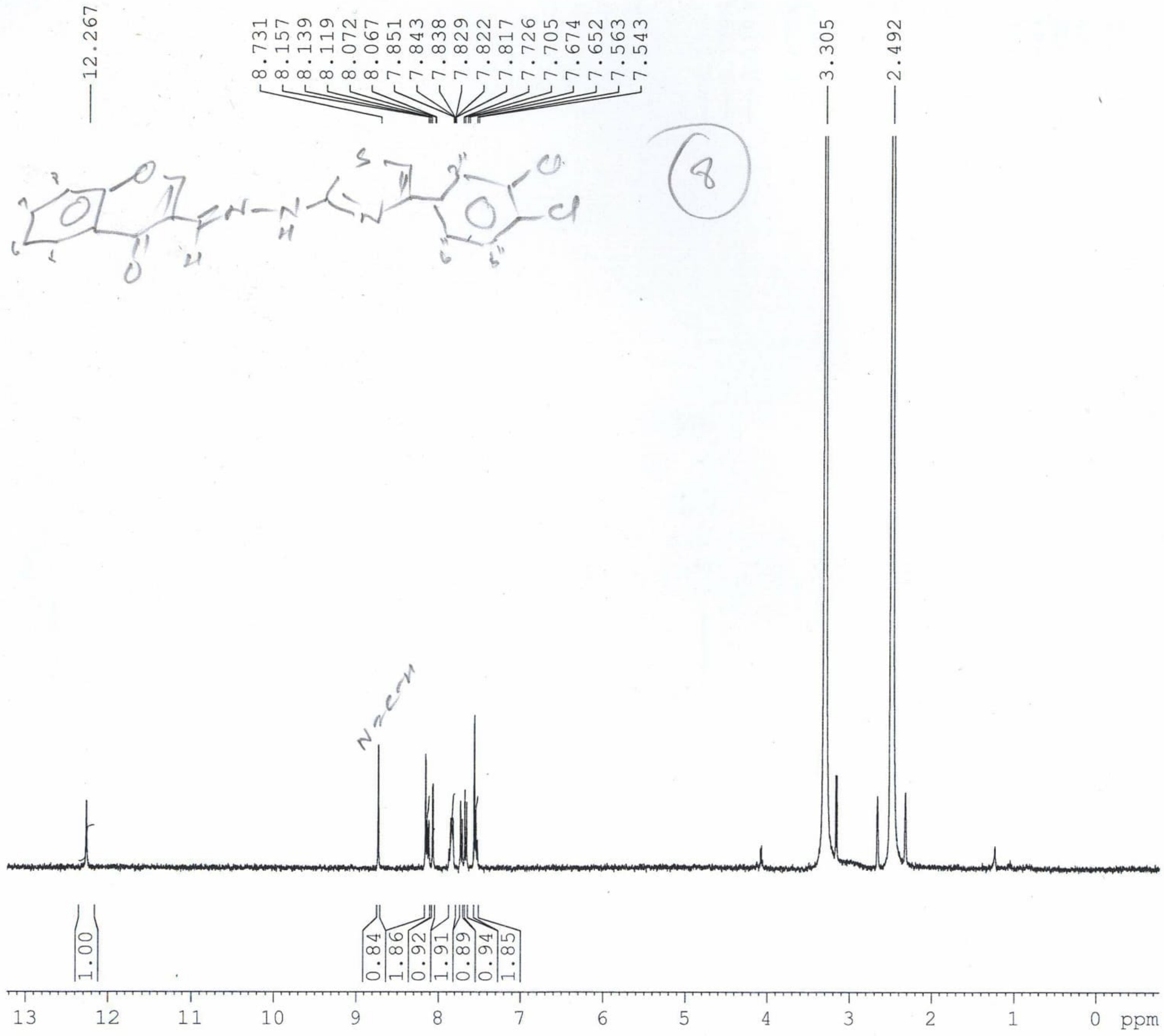

NAME	mar03-15
EXPNO	9
PROCNO	1
Date_	20150303
Time	9.48
INSTRUM	spect
PROBHD	5 mm DUL 13C-1
PULPROG	zg30
TD	32768
SOLVENT	DMSO
NS	64
DS	0
SWH	8012.820 Hz
FIDRES	0.244532 Hz
AQ	2.0447731 sec
RG	362
DW	62.400 usec
DE	6.50 usec
TE	300.0 K
D1	2.00000000 sec
TD0	1

======== CHANNEL f1 ========

NUC1	1H
P1	10.20 usec
PL1	0.00 dB
SFO1	400.1332010 MHz
SI	16384
SF	400.1300064 MHz
WDW	EM
SSB	0
LB	0.30 Hz
GB	0
PC	1.00

File: US-IV-90
Sample: UZMA SALAR /DR. KHALID
Instrument: JEOL JMS 600-H
Inlet: My Inlet

Date Run: 09-10-2014 (Time Run: 12:56:08)

Ionization mode: EI+

Compound 10

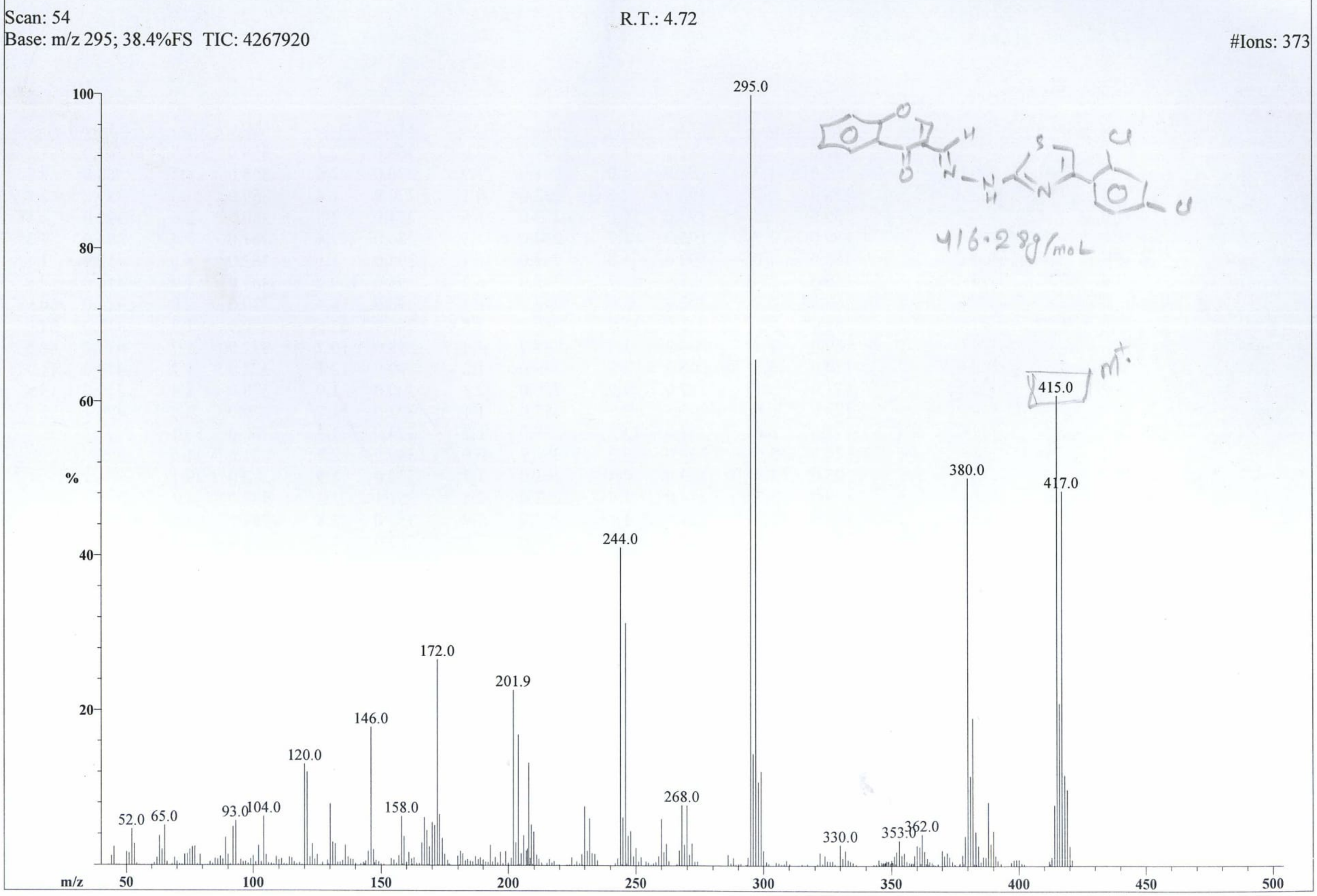

UZMA/DR,KHALID/US-IV.40/
ICCBS,U.O.K/

Compound 10

AVANCE 400
LAB NO 117

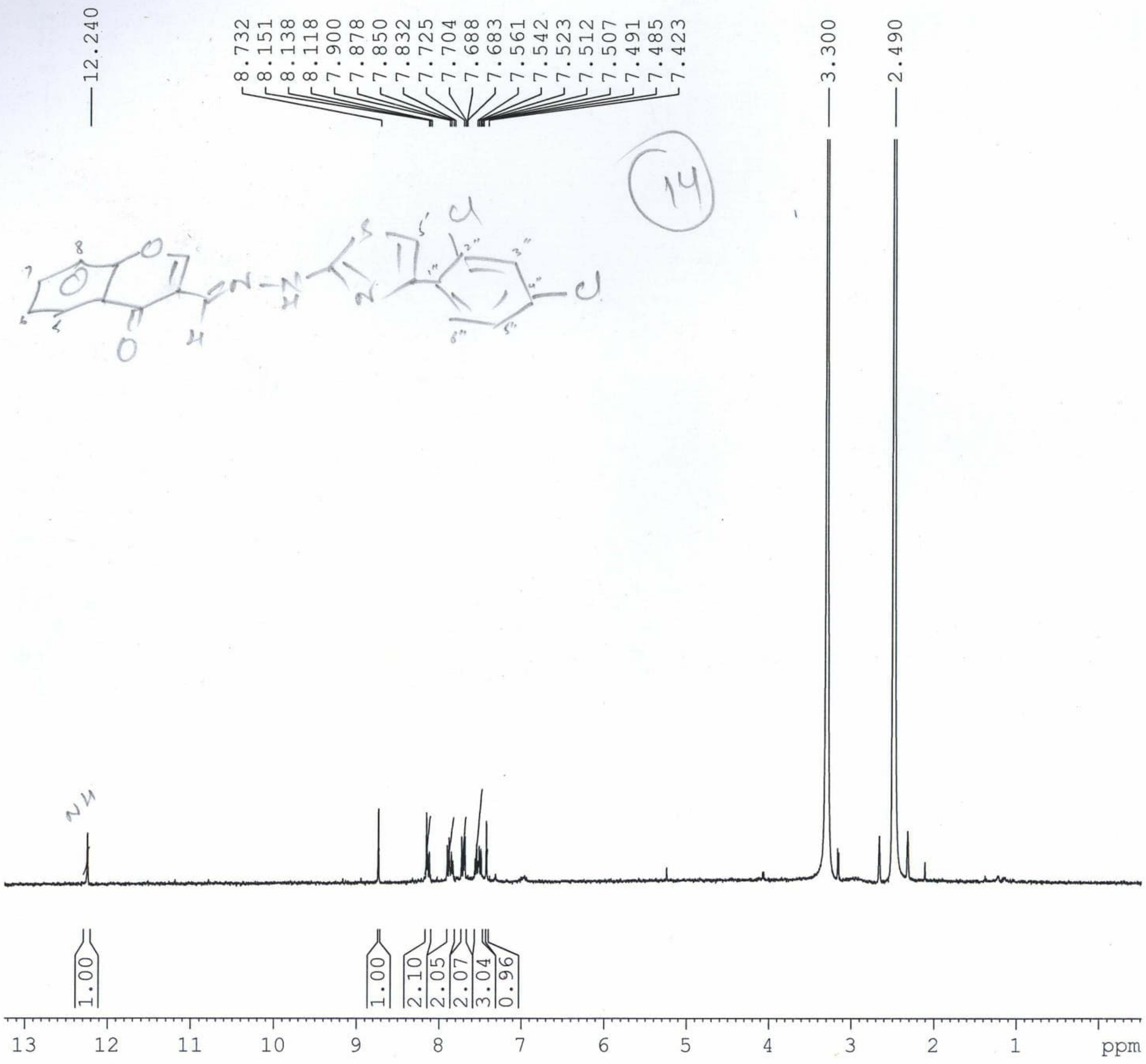

NAME	nov17-14
EXPNO	7
PROCNO	1
Date_	20141117
Time	8.19
INSTRUM	spect
PROBHD	5 mm DUL 13C-1
PULPROG	zg30
TD	32768
SOLVENT	DMSO
NS	64
DS	0
SWH	8012.820 Hz
FIDRES	0.244532 Hz
AQ	2.0447731 sec
RG	362
DW	62.400 usec
DE	6.50 usec
TE	300.0 K
D1	1.50000000 sec
TD0	1

======== CHANNEL f1 ========

NUC1	1H
P1	10.20 usec
PL1	0.00 dB
SFO1	400.1332010 MHz
SI	16384
SF	400.1300064 MHz
WDW	EM
SSB	0
LB	0.30 Hz
GB	0
PC	1.00

File: US-IV-91 **Date Run: 09-10-2014 (Time Run: 14:25:44)**
Sample: UZMA SALAR /DR. KHALID
Instrument: JEOL JMS 600-H
Inlet: My Inlet **Ionization mode: EI+**

Compound 11

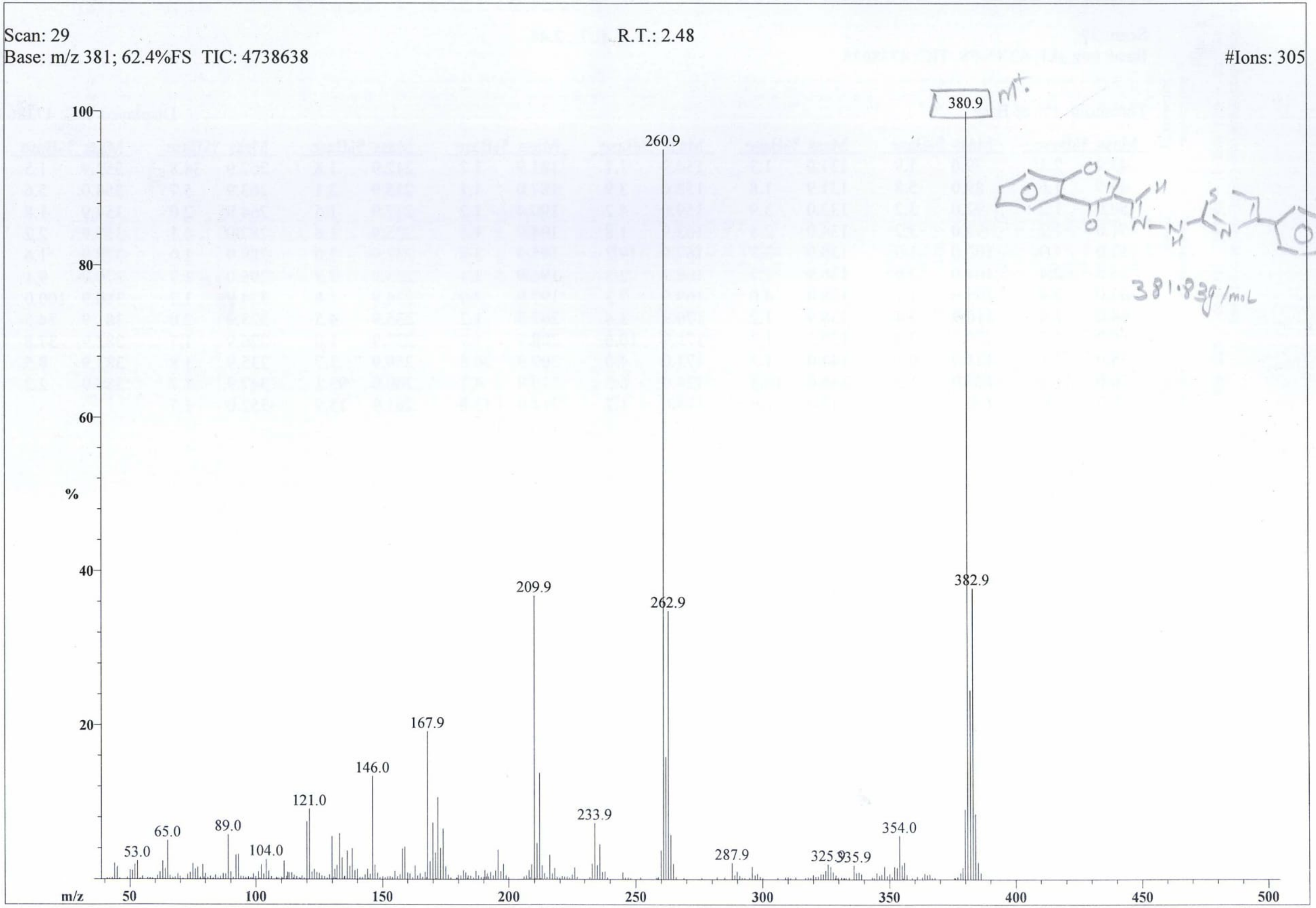

UZMA/DR,KHALID/US-IV-91/
ICCBS,U.O.K/

Compound 11

AVANCE 400
LAB NO 117

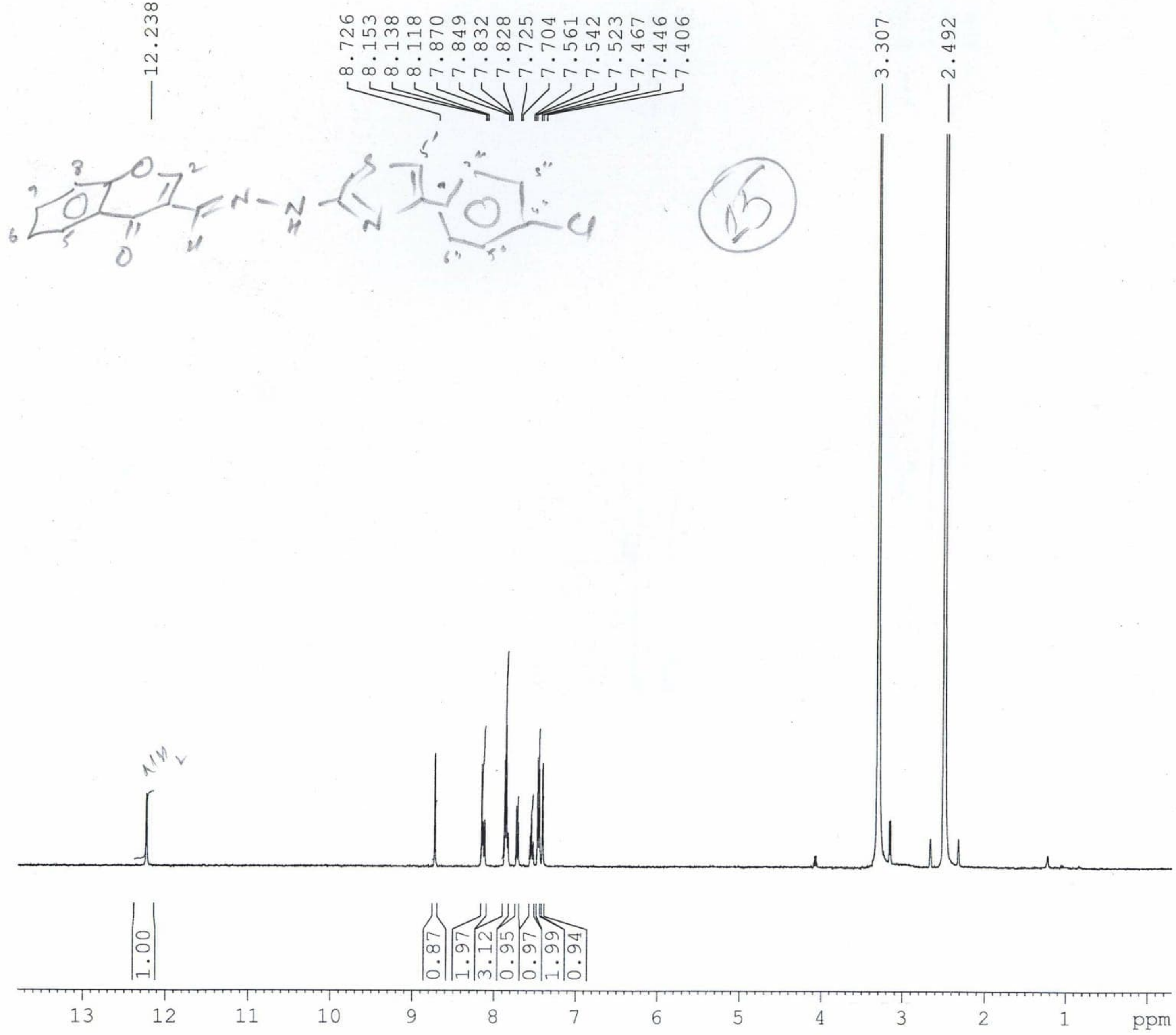

NAME	mar03-15
EXPNO	12
PROCNO	1
Date_	20150303
Time	10.14
INSTRUM	spect
PROBHD	5 mm DUL 13C-1
PULPROG	zg30
TD	32768
SOLVENT	DMSO
NS	64
DS	0
SWH	8012.820 Hz
FIDRES	0.244532 Hz
AQ	2.0447731 sec
RG	362
DW	62.400 usec
DE	6.50 usec
TE	300.0 K
D1	2.00000000 sec
TD0	1

======== CHANNEL f1 ========

NUC1	1H
P1	10.20 usec
PL1	0.00 dB
SFO1	400.1332010 MHz
SI	16384
SF	400.1300064 MHz
WDW	EM
SSB	0
LB	0.30 Hz
GB	0
PC	1.00

File: US-IV-84
Sample: UZMA SALAR /DR. KHALID
Instrument: JEOL JMS 600-H
Inlet: My Inlet

Date Run: 09-10-2014 (Time Run: 15:47:41)

Compound 12

Ionization mode: EI+

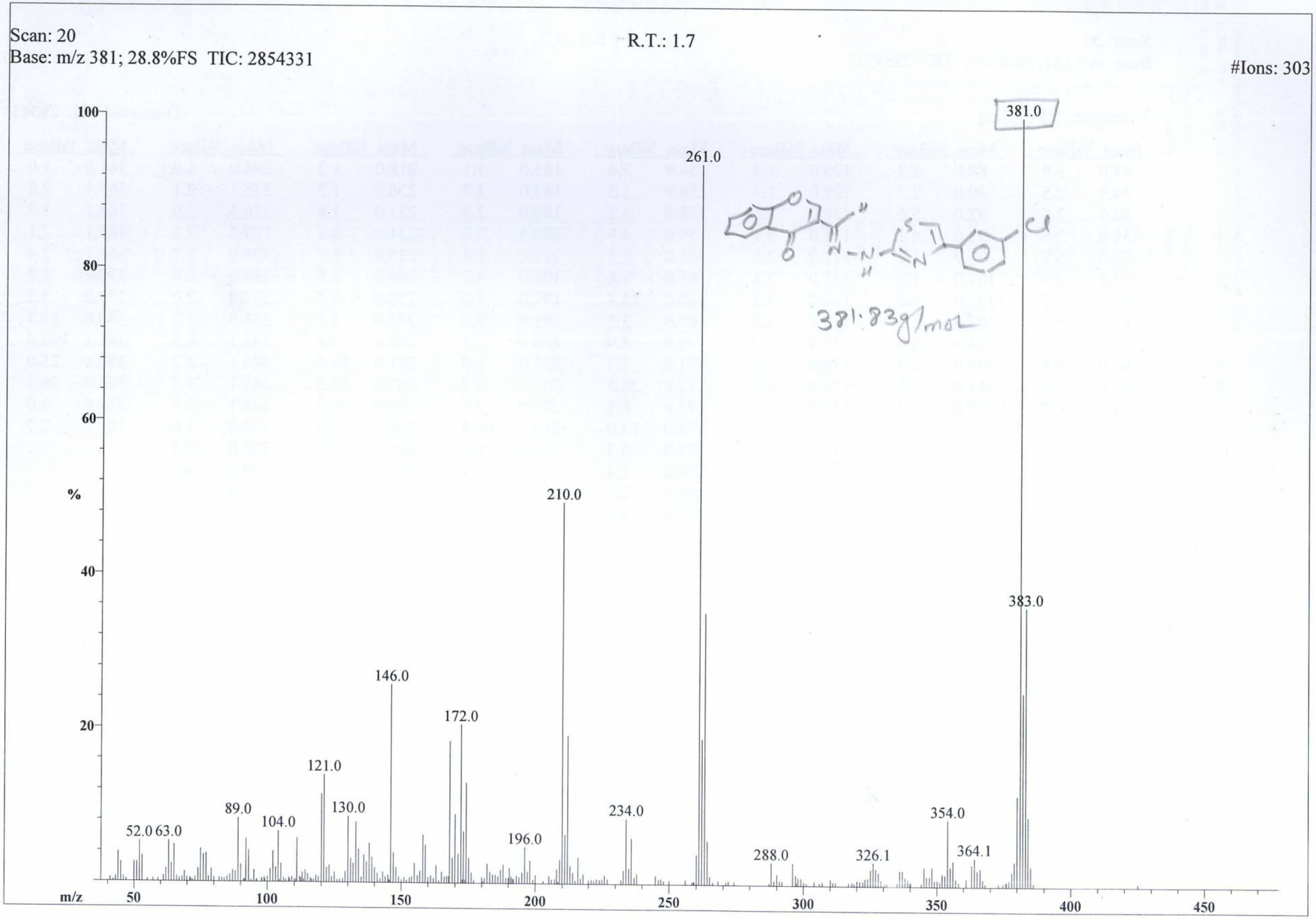

UZMA/DR,KHALID/US-IV-84/
ICCBS,U.O.K/

Compound 12

AVANCE 400
LAB NO 117

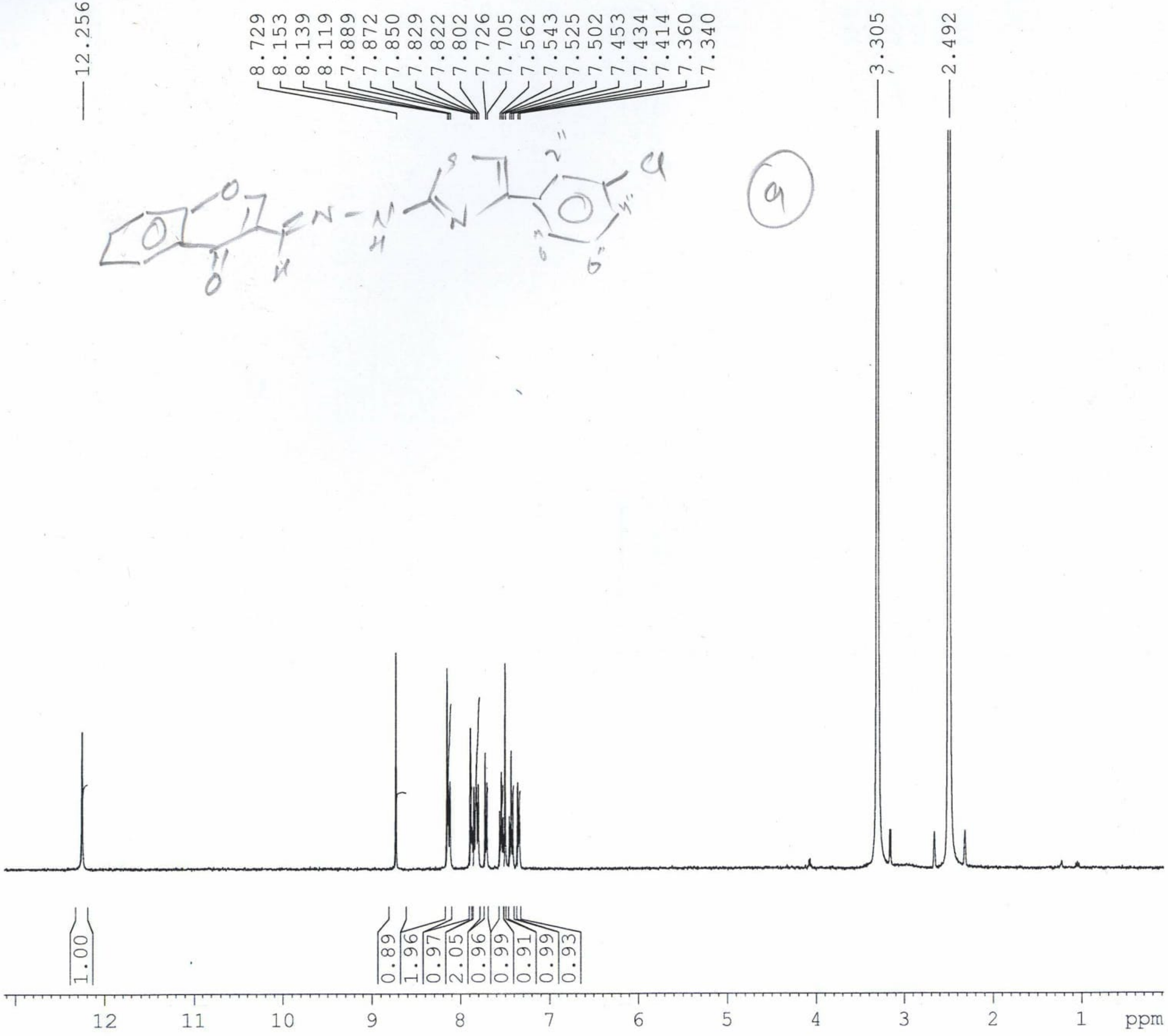

NAME	mar03-15
EXPNO	10
PROCNO	1
Date_	20150303
Time	9.57
INSTRUM	spect
PROBHD	5 mm DUL 13C-1
PULPROG	zg30
TD	32768
SOLVENT	DMSO
NS	64
DS	0
SWH	8012.820 Hz
FIDRES	0.244532 Hz
AQ	2.0447731 sec
RG	362
DW	62.400 usec
DE	6.50 usec
TE	300.0 K
D1	2.00000000 sec
TD0	1

======== CHANNEL f1 ========

NUC1	1H
P1	10.20 usec
PL1	0.00 dB
SFO1	400.1332010 MHz
SI	16384
SF	400.1300064 MHz
WDW	EM
SSB	0
LB	0.30 Hz
GB	0
PC	1.00

File: US-IV-86
Sample: UZMA SALAR /DR. KHALID
Instrument: JEOL JMS 600-H
Inlet: My Inlet

Date Run: 09-10-2014 (Time Run: 15:55:31)

Ionization mode: EI+

Compound 13

Scan: 17
Base: m/z 272; 33%FS TIC: 2774247
R.T.: 1.43
#Ions: 315

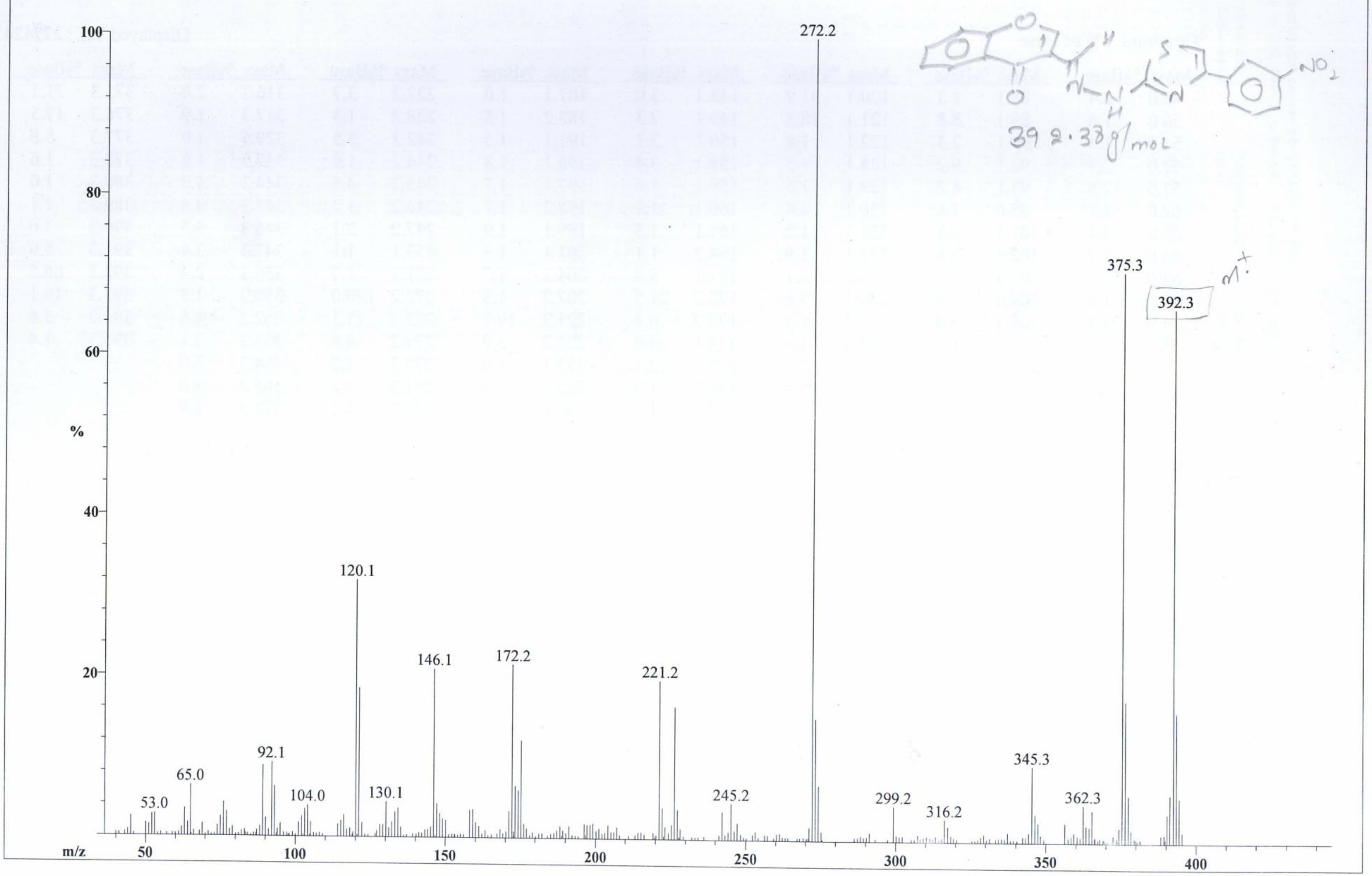

UZMA/DR,KHALID/US-IV-86/
ICCBS,U.O.K/

Compound 13

AVANCE 400
LAB NO 117

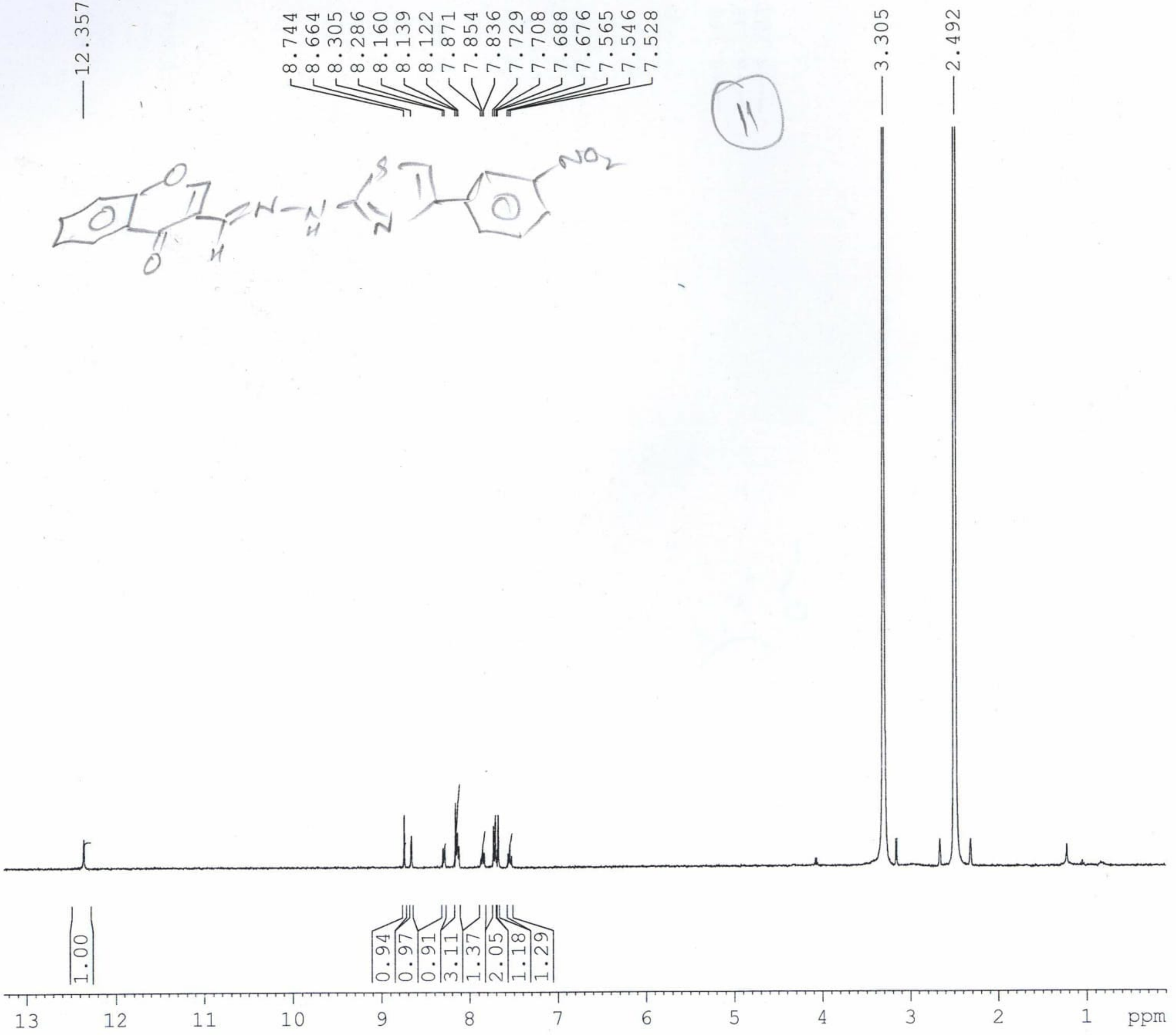

NAME	mar03-15
EXPNO	11
PROCNO	1
Date_	20150303
Time	10.05
INSTRUM	spect
PROBHD	5 mm DUL 13C-1
PULPROG	zg30
TD	32768
SOLVENT	DMSO
NS	64
DS	0
SWH	8012.820 Hz
FIDRES	0.244532 Hz
AQ	2.0447731 sec
RG	362
DW	62.400 usec
DE	6.50 usec
TE	300.0 K
D1	2.00000000 sec
TD0	1

======== CHANNEL f1 ========

NUC1	1H
P1	10.20 usec
PL1	0.00 dB
SFO1	400.1332010 MHz
SI	16384
SF	400.1300064 MHz
WDW	EM
SSB	0
LB	0.30 Hz
GB	0
PC	1.00

File: US-IV-88 **Date Run: 09-10-2014 (Time Run: 16:00:56)** Compound 14
Sample: UZMA SALAR /DR. KHALID
Instrument: JEOL JMS 600-H
Inlet: My Inlet **Ionization mode: EI+**

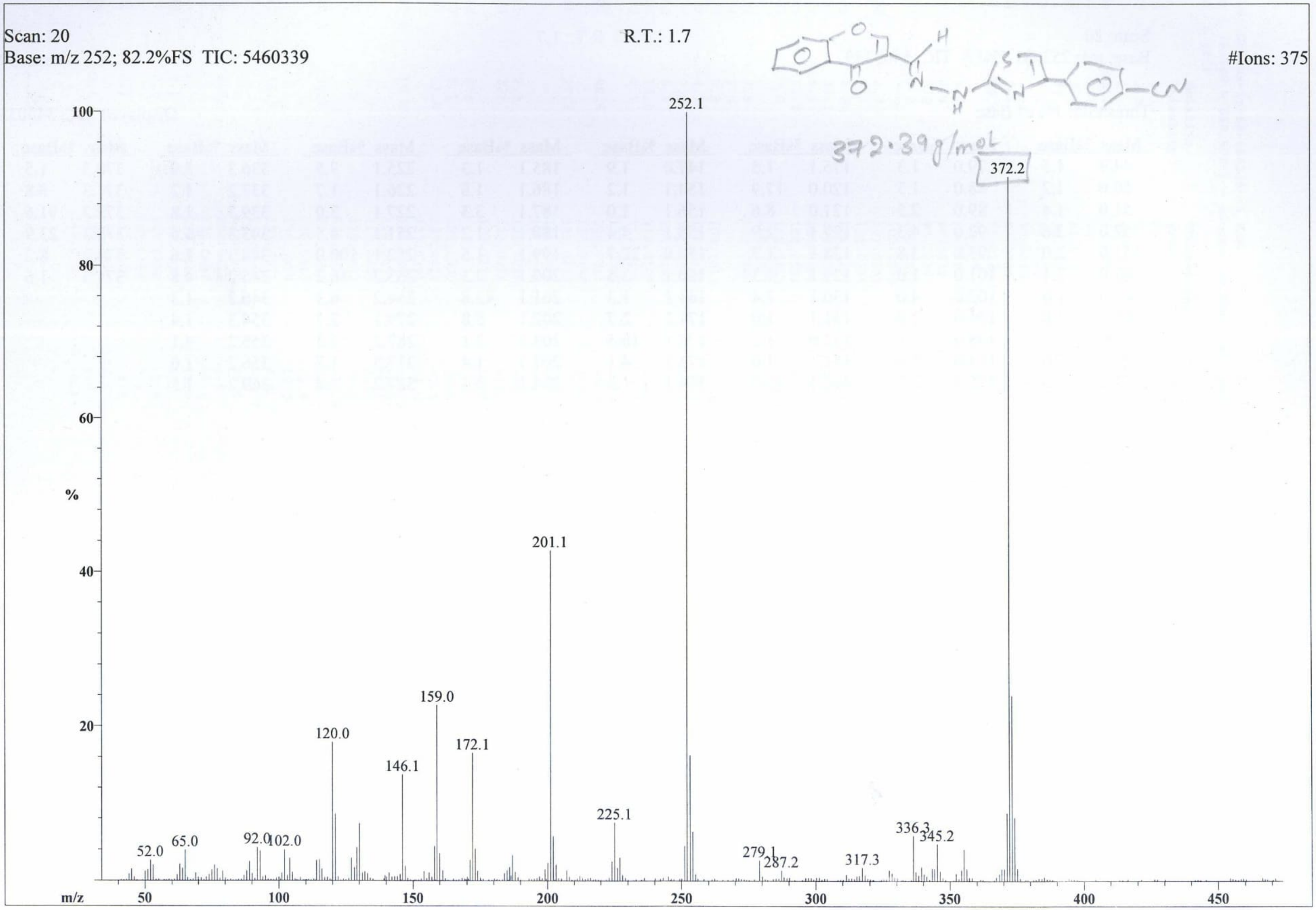

UZMA/DR,KHALID/US-IV-88/
ICCBS,U.O.K/

Compound 14

AVANCE 400
LAB NO 117

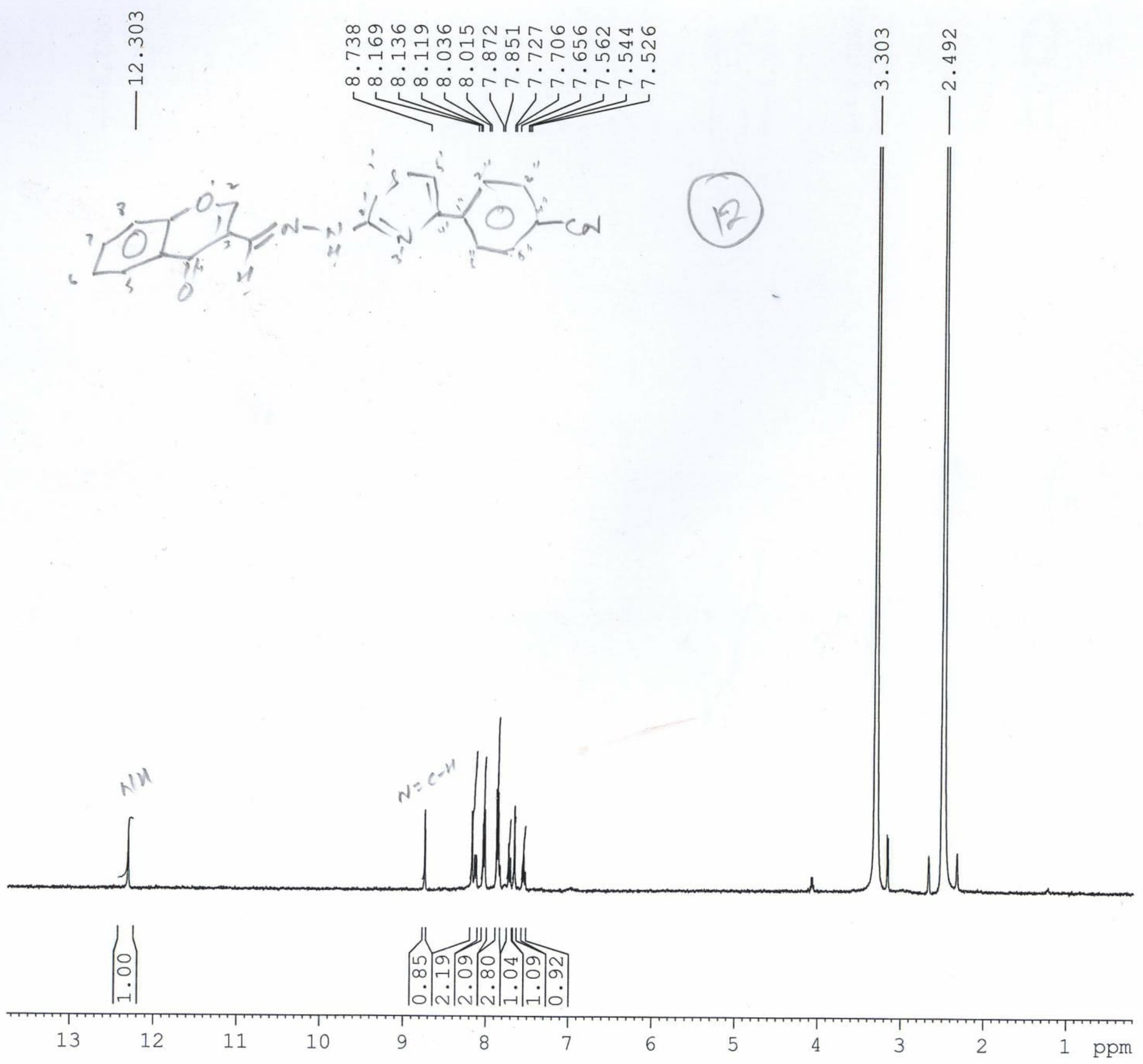

NAME	jan27-15	
EXPNO	10	
PROCNO	1	
Date_	20150127	
Time	10.00	
INSTRUM	spect	
PROBHD	5 mm DUL 13C-1	
PULPROG	zg30	
TD	32768	
SOLVENT	DMSO	
NS	64	
DS	0	
SWH	8012.820	Hz
FIDRES	0.244532	Hz
AQ	2.0447731	sec
RG	362	
DW	62.400	usec
DE	6.50	usec
TE	300.0	K
D1	1.50000000	sec
TD0	1	

======== CHANNEL f1 ========

NUC1	1H	
P1	10.20	usec
PL1	0.00	dB
SFO1	400.1332010	MHz
SI	16384	
SF	400.1300064	MHz
WDW	EM	
SSB	0	
LB	0.30	Hz
GB	0	
PC	1.00	

File: US-IV-89
Sample: UZMA SALAR /DR. KHALID
Instrument: JEOL JMS 600-H
Inlet: My Inlet

Date Run: 09-10-2014 (Time Run: 12:49:45)
Ionization mode: EI+

Compound 15

Scan: 21
Base: m/z 423; 63.2%FS TIC: 3370272
R.T.: 1.78
#Ions: 327

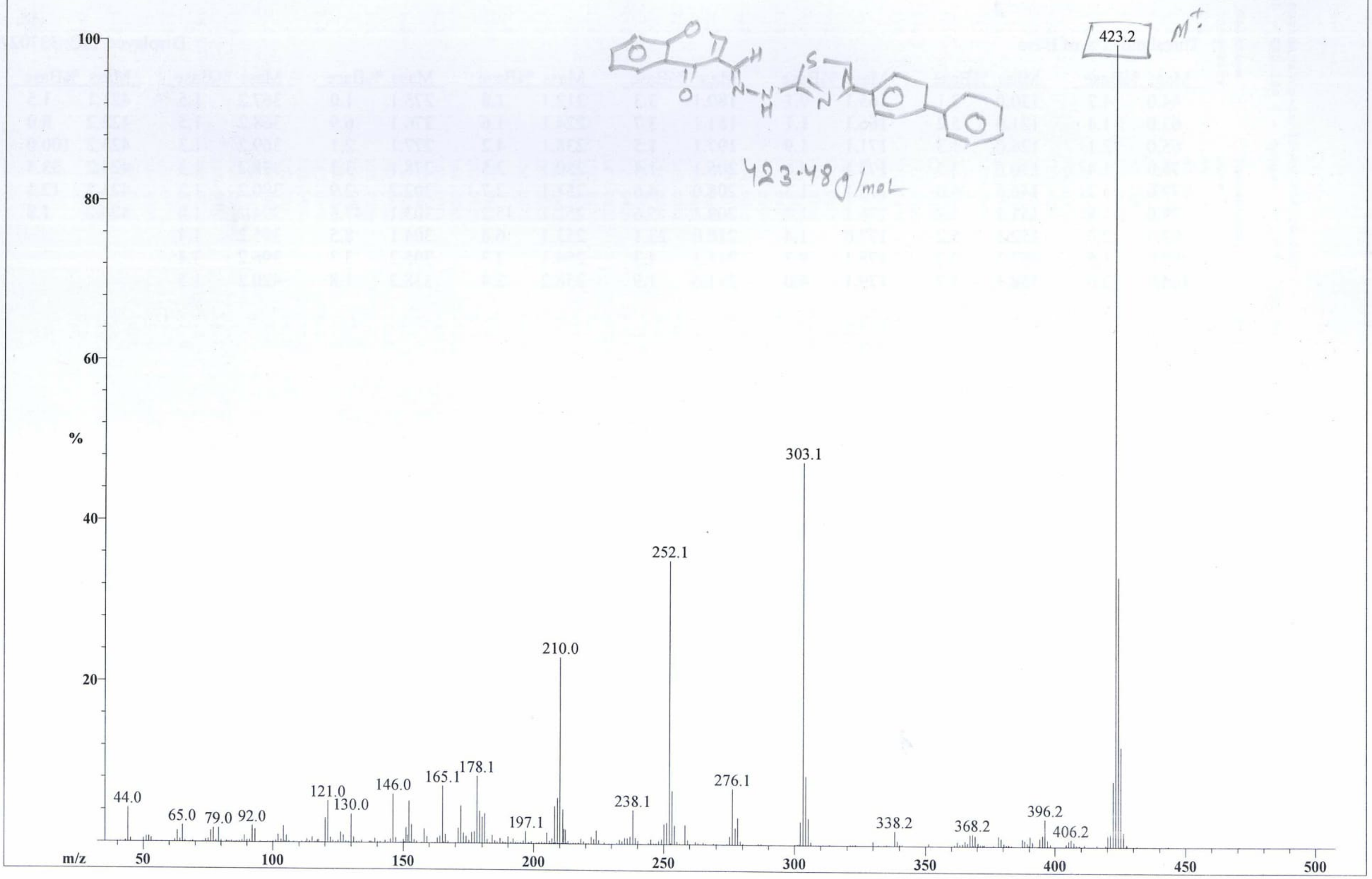

UZMA/DR,KHALID/US-IV-89/
ICCBS,U.O.K/

Compound 15

AVANCE 400
LAB NO 117

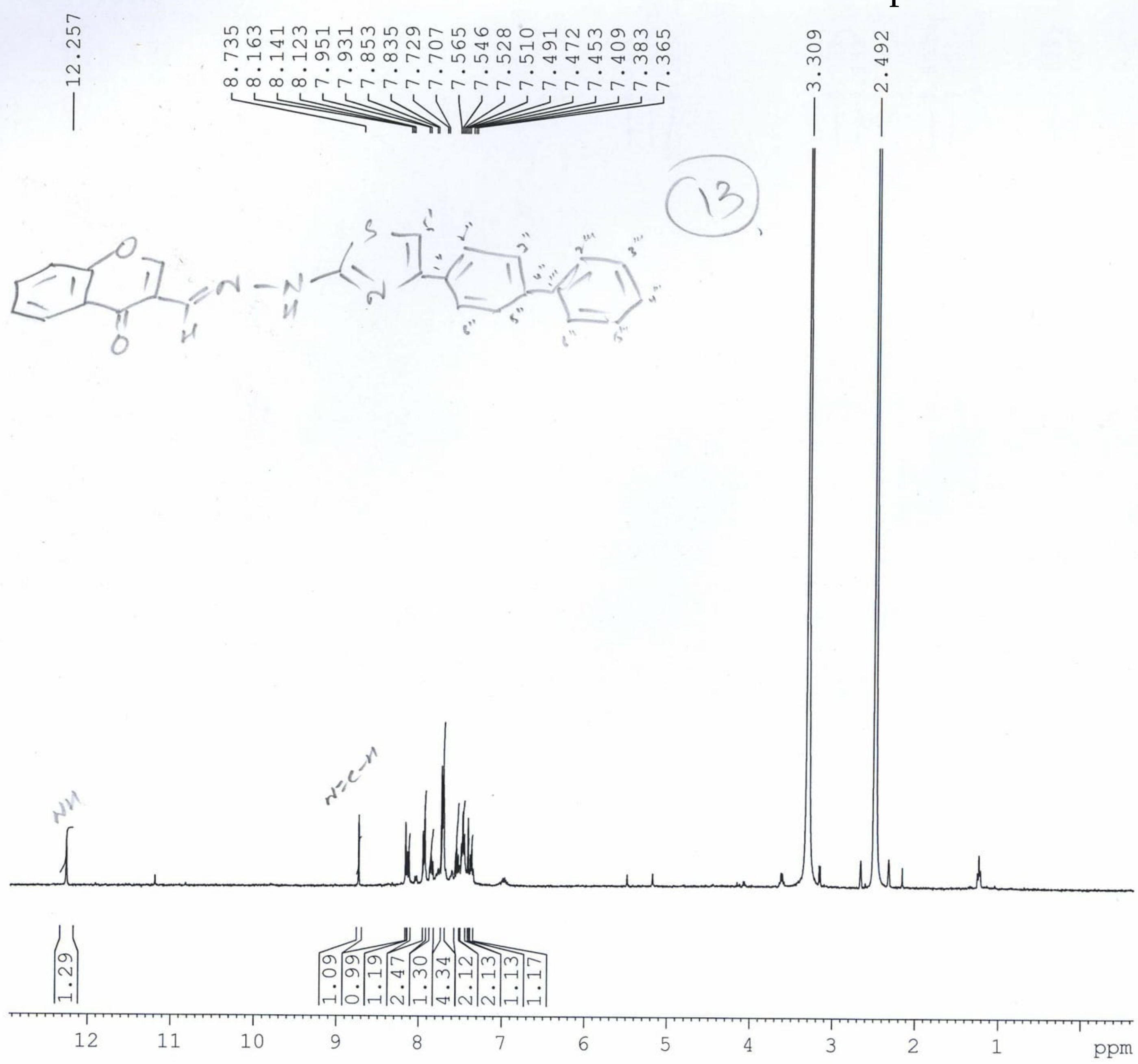

NAME	sep18-14	
EXPNO	8	
PROCNO	1	
Date_	20140918	
Time	10.44	
INSTRUM	spect	
PROBHD	5 mm DUL 13C-1	
PULPROG	zg30	
TD	32768	
SOLVENT	DMSO	
NS	64	
DS	0	
SWH	8012.820	Hz
FIDRES	0.244532	Hz
AQ	2.0447731	sec
RG	2048	
DW	62.400	usec
DE	6.50	usec
TE	300.0	K
D1	2.00000000	sec
TD0	1	

======== CHANNEL f1 ========

NUC1	1H	
P1	10.20	usec
PL1	0.00	dB
SFO1	400.1332010	MHz
SI	16384	
SF	400.1300064	MHz
WDW	EM	
SSB	0	
LB	0.30	Hz
GB	0	
PC	1.00	

File: US-IV-97 **Date Run: 09-10-2014 (Time Run: 14:52:34)** Compound 16
Sample: UZMA SALAR /DR. KHALID
Instrument: JEOL JMS 600-H
Inlet: My Inlet **Ionization mode: EI+**

Scan: 25 R.T.: 2.13
Base: m/z 361; 99.5%FS TIC: 7215591 #Ions: 363

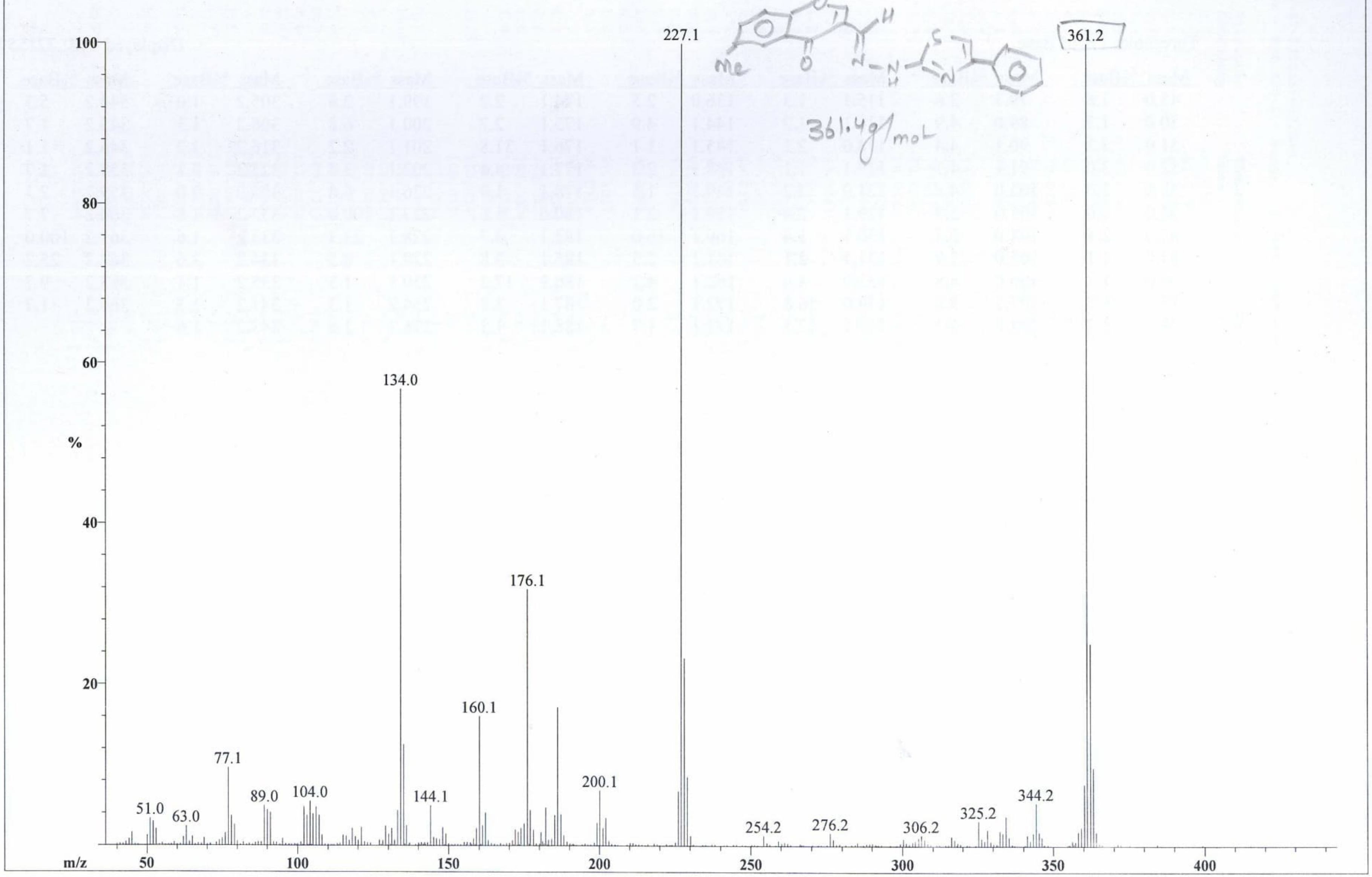

Compound 16

UZMA/DR,KHALID/US-IV-97/
ICCBS,U.O.K/

AVANCE 400
LAB NO 117

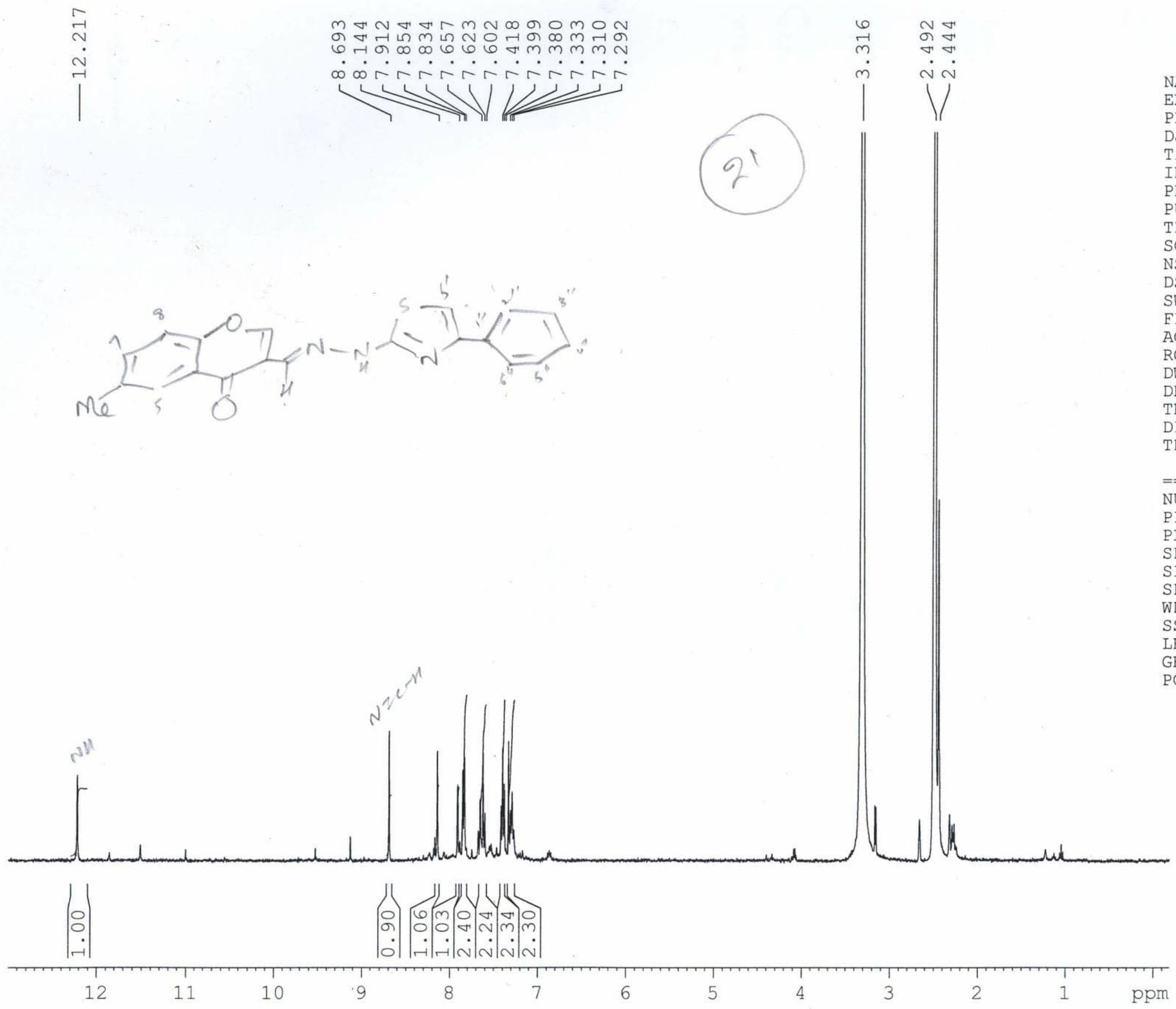

NAME	sep18-14
EXPNO	6
PROCNO	1
Date_	20140918
Time	10.25
INSTRUM	spect
PROBHD	5 mm DUL 13C-1
PULPROG	zg30
TD	32768
SOLVENT	DMSO
NS	64
DS	0
SWH	8012.820 Hz
FIDRES	0.244532 Hz
AQ	2.0447731 sec
RG	2048
DW	62.400 usec
DE	6.50 usec
TE	300.0 K
D1	2.00000000 sec
TD0	1

======== CHANNEL f1 ========

NUC1	1H
P1	10.20 usec
PL1	0.00 dB
SFO1	400.1332010 MHz
SI	16384
SF	400.1300064 MHz
WDW	EM
SSB	0
LB	0.30 Hz
GB	0
PC	1.00

File: US-IV-98
Sample: UZMA SALAR /DR. KHALID
Instrument: JEOL JMS 600-H
Inlet: My Inlet

Date Run: 09-10-2014 (Time Run: 16:14:21)

Ionization mode: EI+

Compound 17

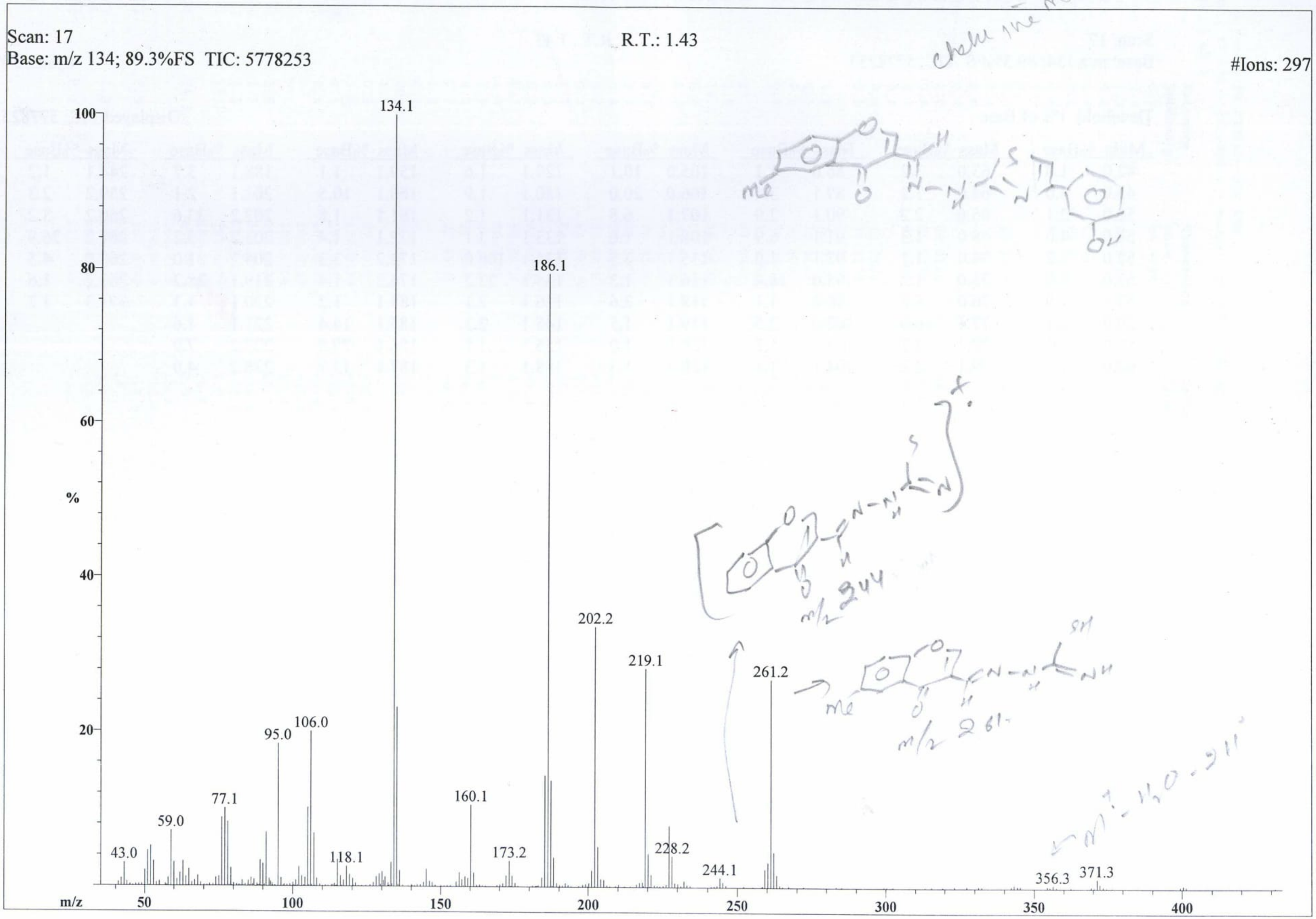

UZMA/DR,KHALID/US-IV-98/
ICCBS,U.O.K/

Compound 17

AVANCE 400
LAB NO 117

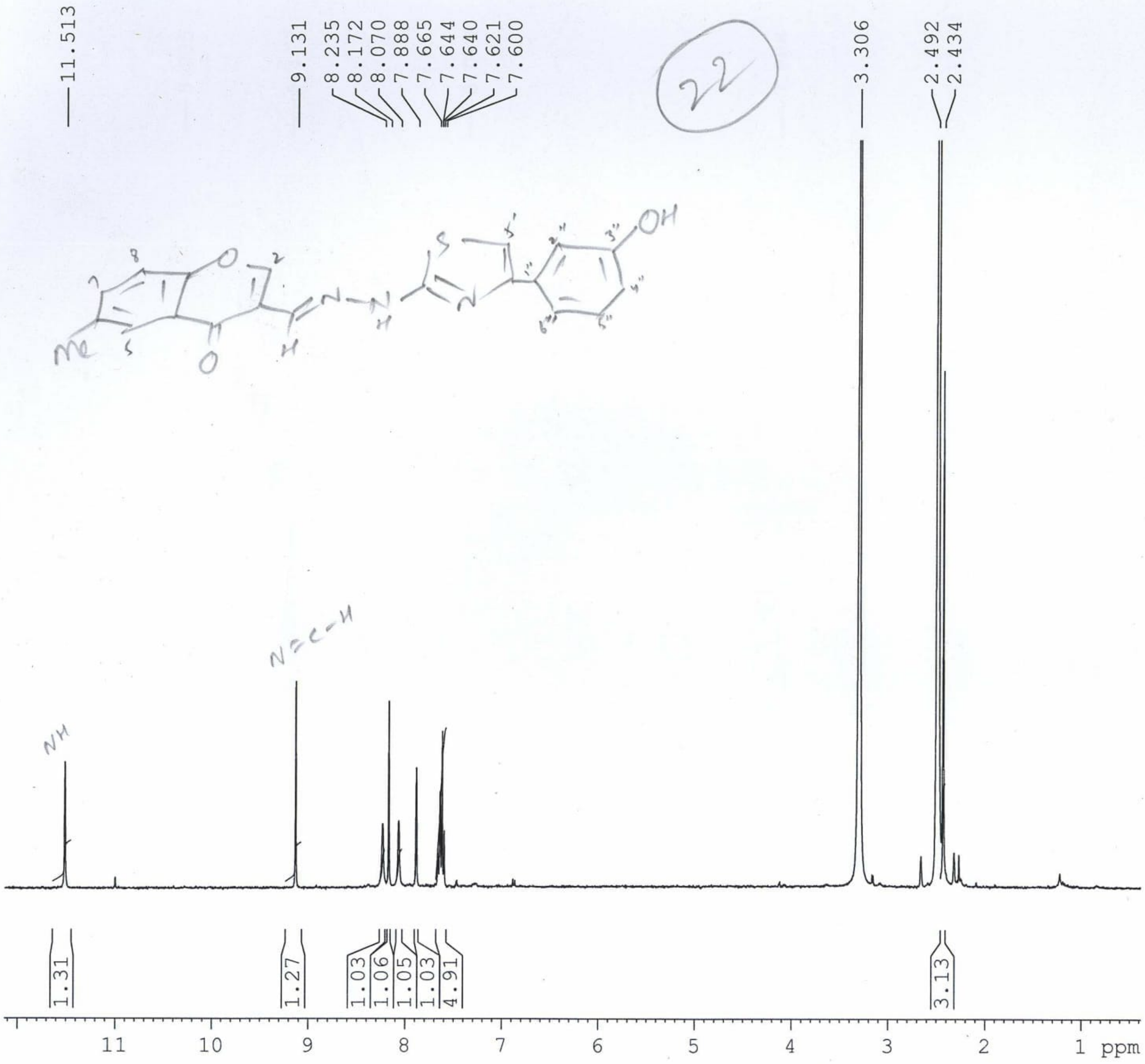

NAME	sep18-14
EXPNO	7
PROCNO	1
Date_	20140918
Time	10.36
INSTRUM	spect
PROBHD	5 mm DUL 13C-1
PULPROG	zg30
TD	32768
SOLVENT	DMSO
NS	64
DS	0
SWH	8012.820 Hz
FIDRES	0.244532 Hz
AQ	2.0447731 sec
RG	2048
DW	62.400 usec
DE	6.50 usec
TE	300.0 K
D1	2.00000000 sec
TD0	1

======== CHANNEL f1 ========

NUC1	1H
P1	10.20 usec
PL1	0.00 dB
SFO1	400.1332010 MHz
SI	16384
SF	400.1300064 MHz
WDW	EM
SSB	0
LB	0.30 Hz
GB	0
PC	1.00

File: US-V-6
Sample: UZMA SALAR /DR. KHALID
Instrument: JEOL JMS 600-H
Inlet: My Inlet

Date Run: 09-11-2014 (Time Run: 09:06:03)
Ionization mode: EI+

Compound 18

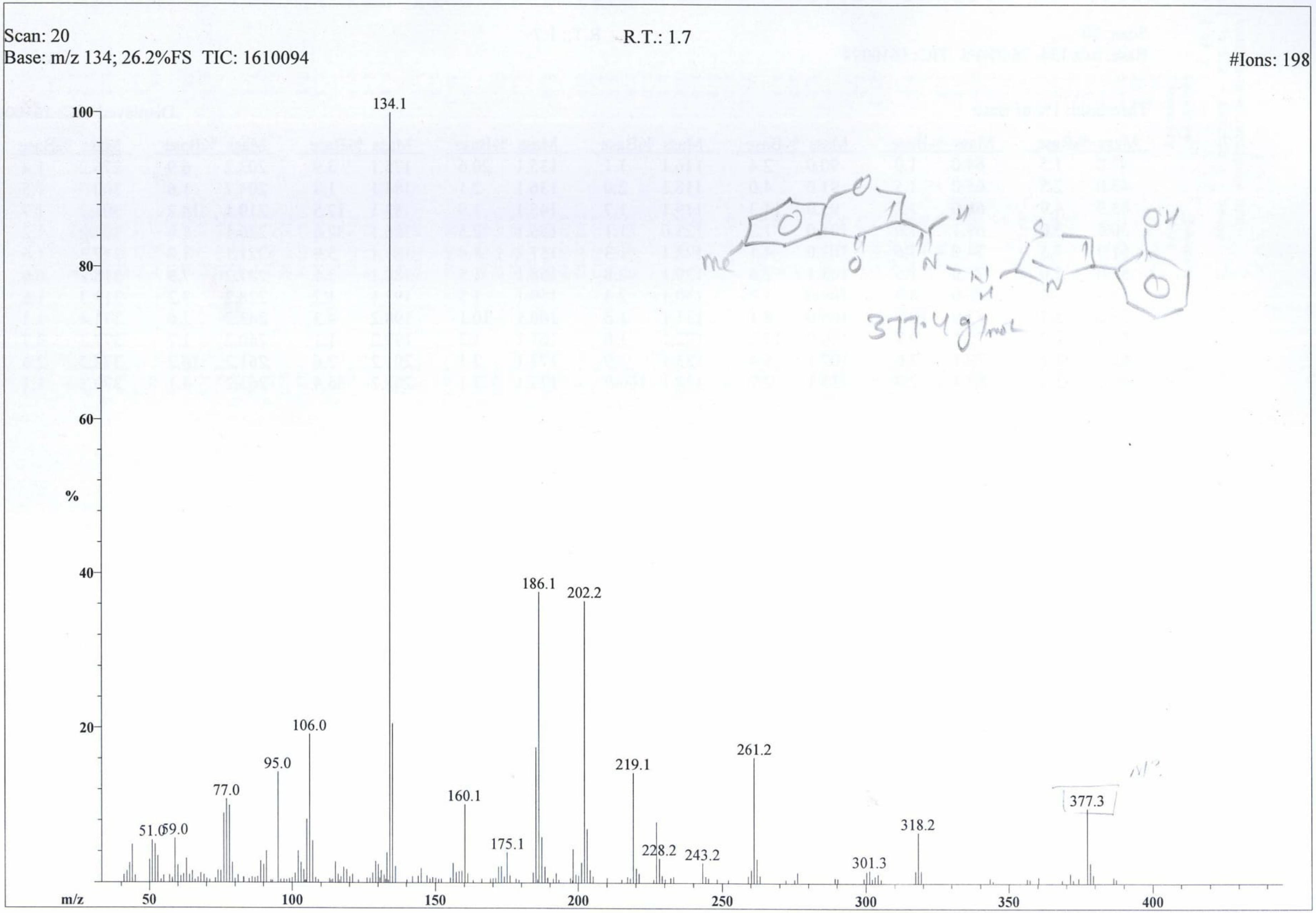

UZMA/DR,KHALID/US-IV-6/
ICCBS,U.O.K/

Compound 18

AVANCE 400
LAB NO 117

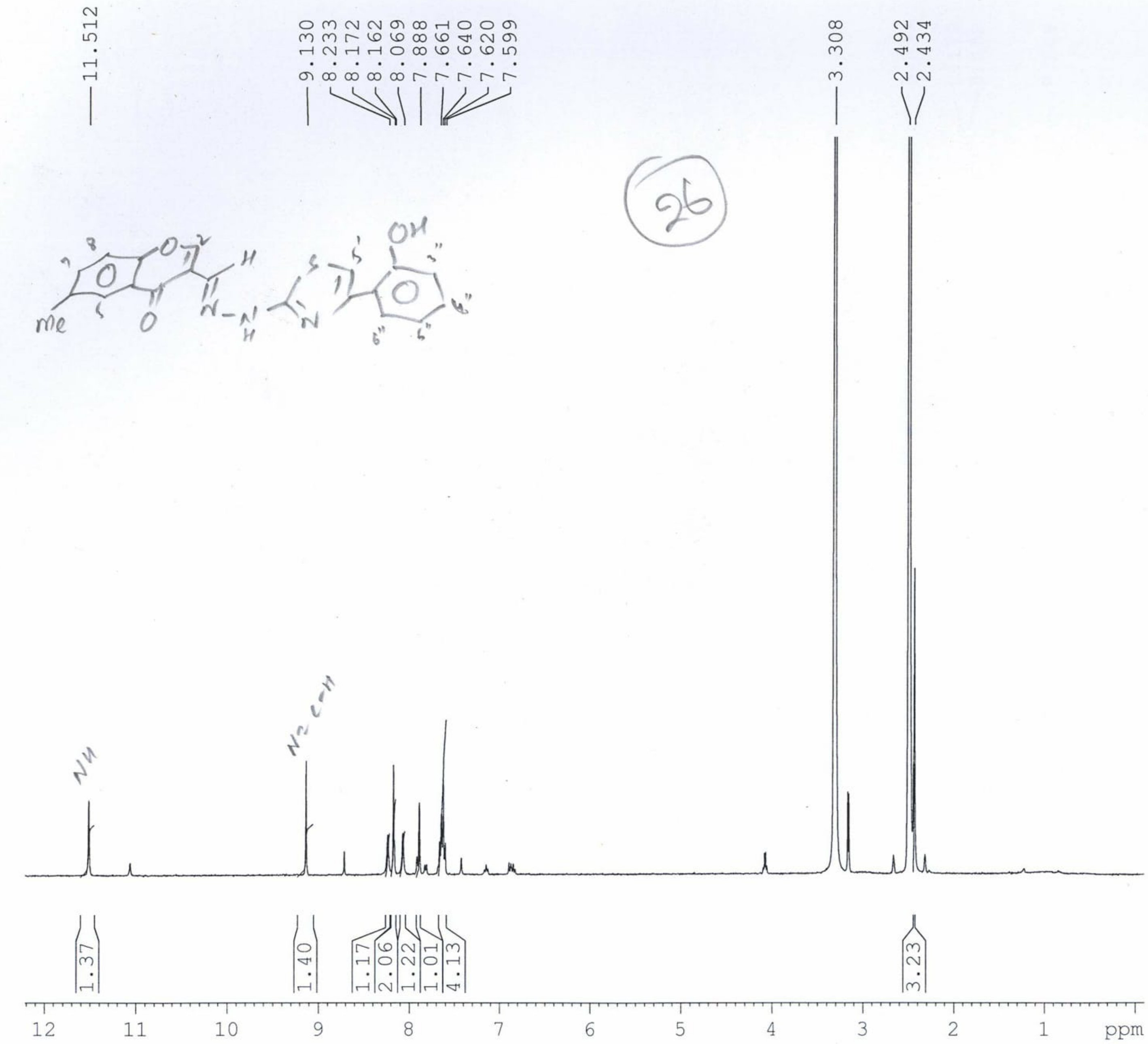

NAME	jan30-15	
EXPNO	9	
PROCNO	1	
Date_	20150130	
Time	9.28	
INSTRUM	spect	
PROBHD	5 mm DUL 13C-1	
PULPROG	zg30	
TD	32768	
SOLVENT	DMSO	
NS	64	
DS	0	
SWH	8012.820	Hz
FIDRES	0.244532	Hz
AQ	2.0447731	sec
RG	362	
DW	62.400	usec
DE	6.50	usec
TE	300.0	K
D1	2.00000000	sec
TD0	1	

======== CHANNEL f1 ========

NUC1	1H	
P1	10.20	usec
PL1	0.00	dB
SFO1	400.1332010	MHz
SI	16384	
SF	400.1300064	MHz
WDW	EM	
SSB	0	
LB	0.30	Hz
GB	0	
PC	1.00	

File: US-IV-95
Sample: UZMA SALAR /DR. KHALID
Instrument: JEOL JMS 600-H
Inlet: My Inlet

Date Run: 09-10-2014 (Time Run: 15:19:20)

Ionization mode: EI+

Compound 19

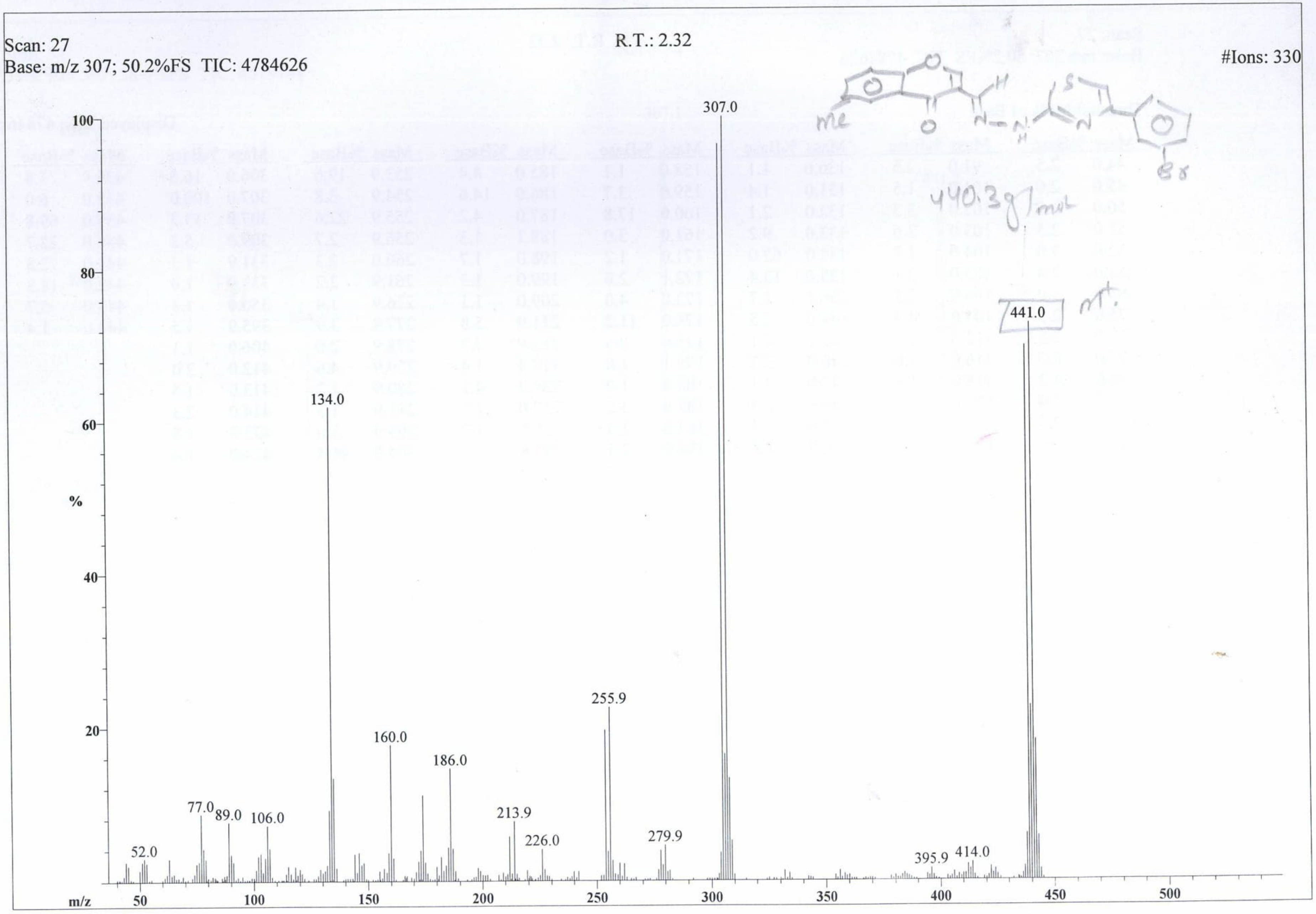

UZMA/DR,KHALID/US-IV-96/
ICCBS,U.O.K/

Compound 19

AVANCE 400
LAB NO 117

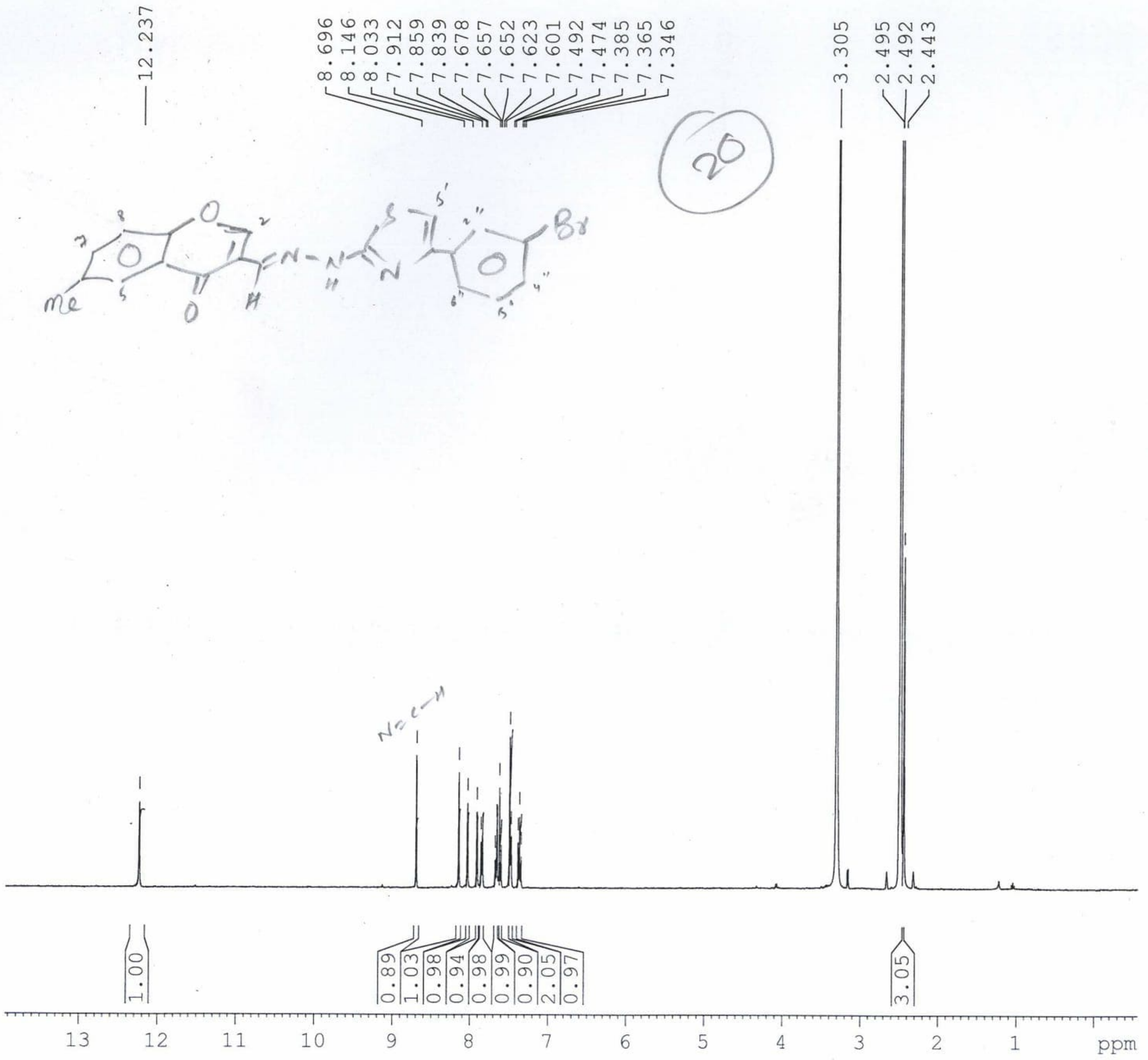

NAME	feb26-15
EXPNO	11
PROCNO	1
Date_	20150226
Time	10.04
INSTRUM	spect
PROBHD	5 mm DUL 13C-1
PULPROG	zg30
TD	32768
SOLVENT	DMSO
NS	64
DS	0
SWH	8012.820 Hz
FIDRES	0.244532 Hz
AQ	2.0447731 sec
RG	362
DW	62.400 usec
DE	6.50 usec
TE	300.0 K
D1	2.00000000 sec
TD0	1

======== CHANNEL f1 ========

NUC1	1H
P1	10.20 usec
PL1	0.00 dB
SFO1	400.1332010 MHz
SI	16384
SF	400.1300064 MHz
WDW	EM
SSB	0
LB	0.30 Hz
GB	0
PC	1.00

Compound 20

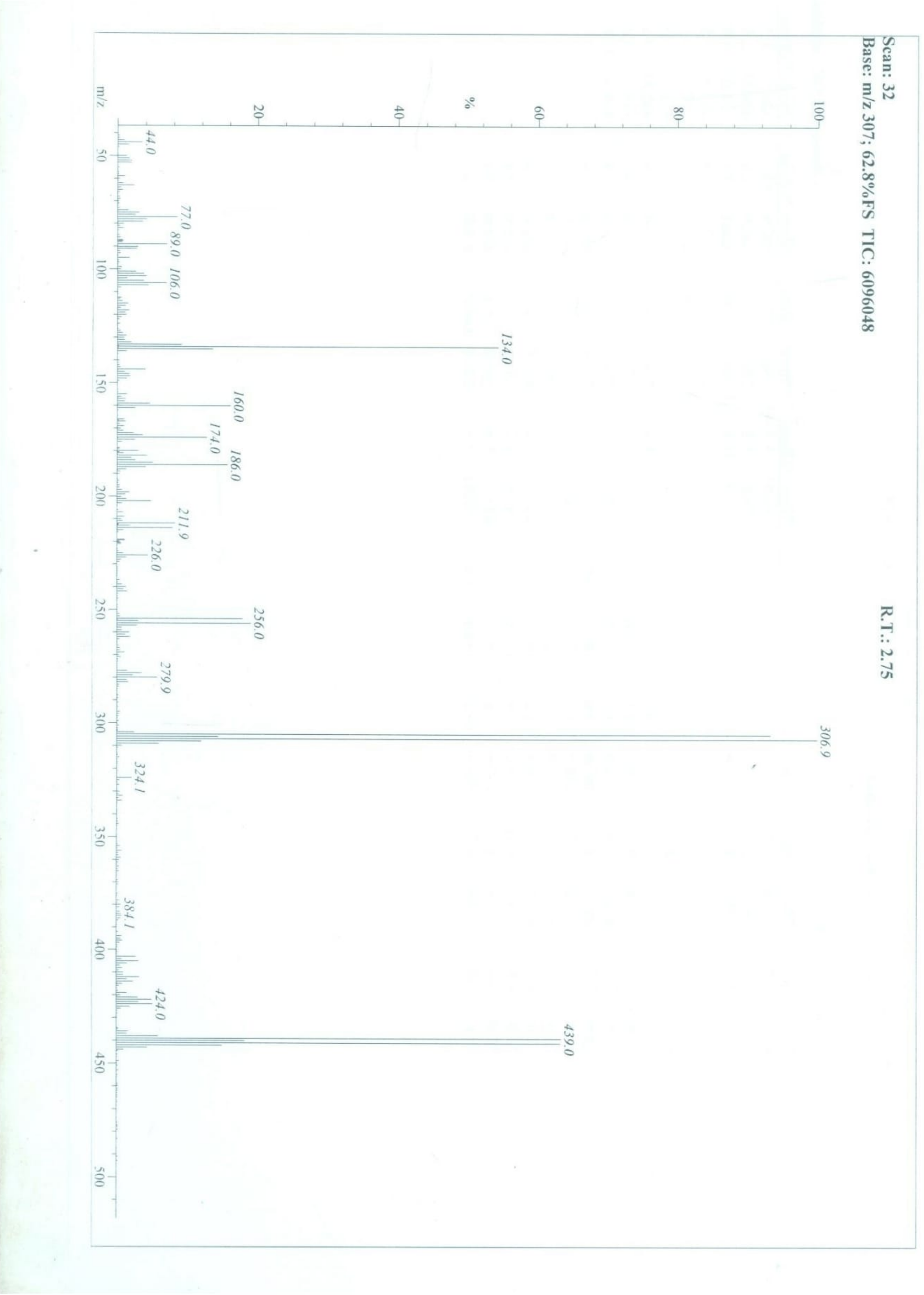
File: US-IV-76
Sample: UZMA SALAR /DR. KHALID
Instrument: JEOL JMS 600-H
Date Run: 09-10-2014 (Time Run: 12:22:18)
Ionization mode: EI+
Scan: 32
Base: m/z 307; 62.8%FS TIC: 6096048
R.T.: 2.75
%
m/z
44.0
77.0
89.0
106.0
134.0
160.0
174.0
186.0
211.9
226.0
256.0
279.9
306.9
324.1
384.1
424.0
439.0

UZMA/DR,KHALID/US-IV-76/
ICCBS,U.O.K/

Compound 20

AVANCE 400
LAB NO 117

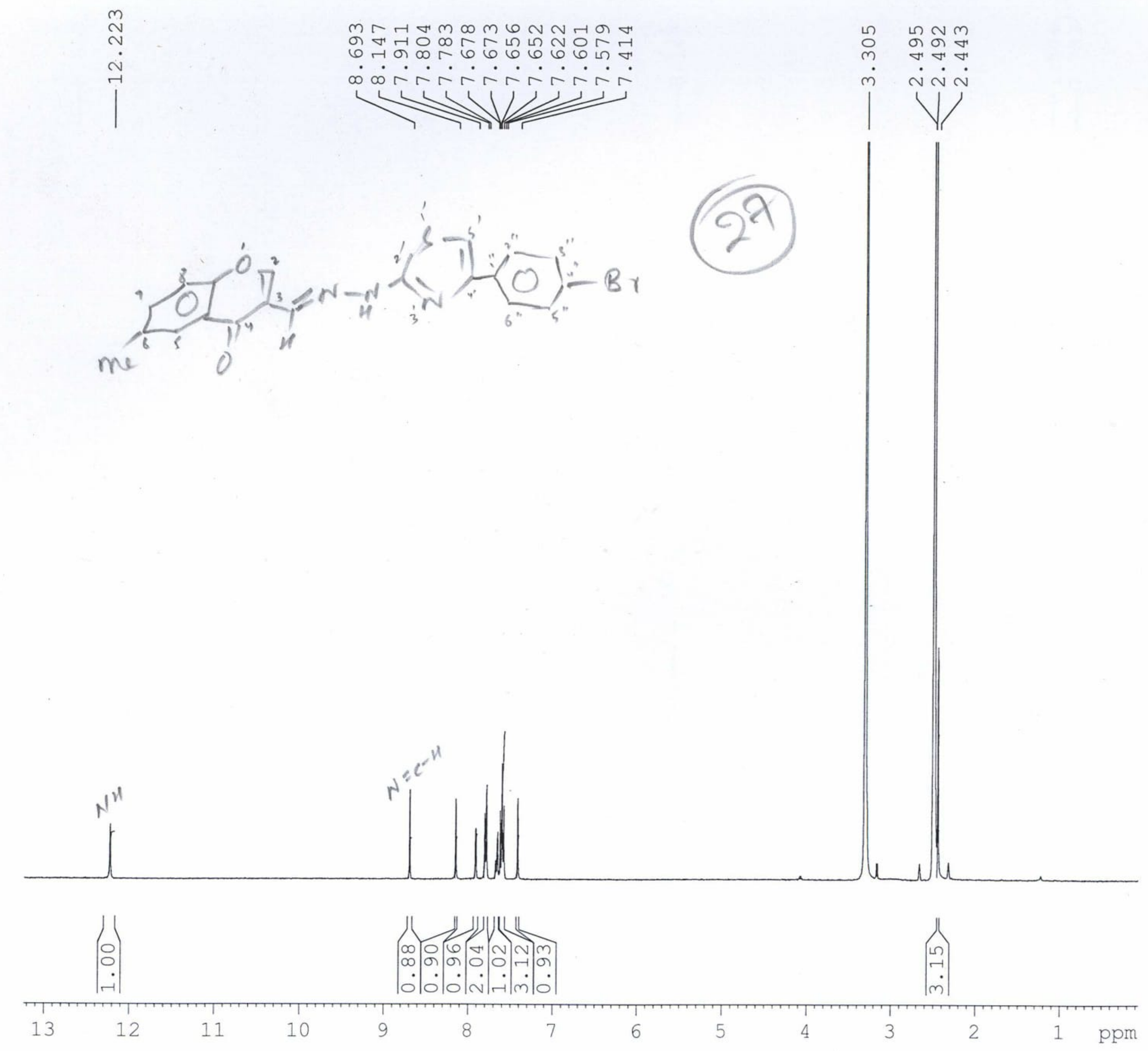

NAME	feb26-15	
EXPNO	8	
PROCNO	1	
Date_	20150226	
Time	9.22	
INSTRUM	spect	
PROBHD	5 mm DUL 13C-1	
PULPROG	zg30	
TD	32768	
SOLVENT	DMSO	
NS	64	
DS	0	
SWH	8012.820	Hz
FIDRES	0.244532	Hz
AQ	2.0447731	sec
RG	362	
DW	62.400	usec
DE	6.50	usec
TE	300.0	K
D1	2.00000000	sec
TD0	1	

======== CHANNEL f1 ========

NUC1	1H	
P1	10.20	usec
PL1	0.00	dB
SFO1	400.1332010	MHz
SI	16384	
SF	400.1300064	MHz
WDW	EM	
SSB	0	
LB	0.30	Hz
GB	0	
PC	1.00	

File: US-V-5 **Date Run: 09-11-2014 (Time Run: 09:13:00)** Compound 21
Sample: UZMA SALAR /DR. KHALID
Instrument: JEOL JMS 600-H
Inlet: My Inlet **Ionization mode: EI+**

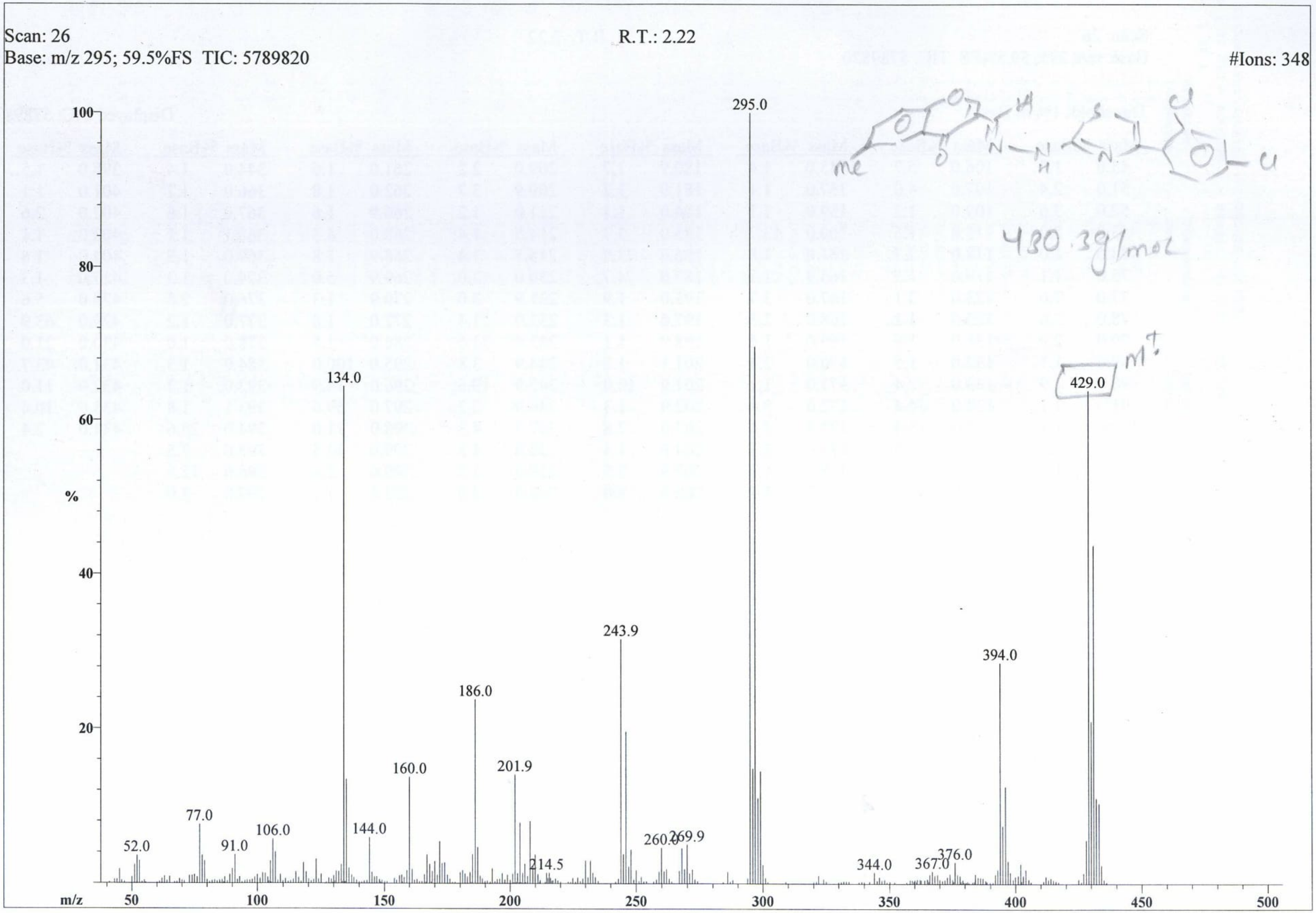

UZMA/DR,KHALID/US-V-5/
ICCBS,U.O.K/

Compound 21

AVANCE 400
LAB NO 117

NAME	nov28-14
EXPNO	6
PROCNO	1
Date_	20141128
Time	10.00
INSTRUM	spect
PROBHD	5 mm DUL 13C-1
PULPROG	zg30
TD	32768
SOLVENT	DMSO
NS	64
DS	0
SWH	8012.820 Hz
FIDRES	0.244532 Hz
AQ	2.0447731 sec
RG	456.1
DW	62.400 usec
DE	6.50 usec
TE	300.0 K
D1	2.00000000 sec
TD0	1

======== CHANNEL f1 ========

NUC1	1H
P1	10.20 usec
PL1	0.00 dB
SFO1	400.1332010 MHz
SI	16384
SF	400.1300064 MHz
WDW	EM
SSB	0
LB	0.30 Hz
GB	0
PC	1.00

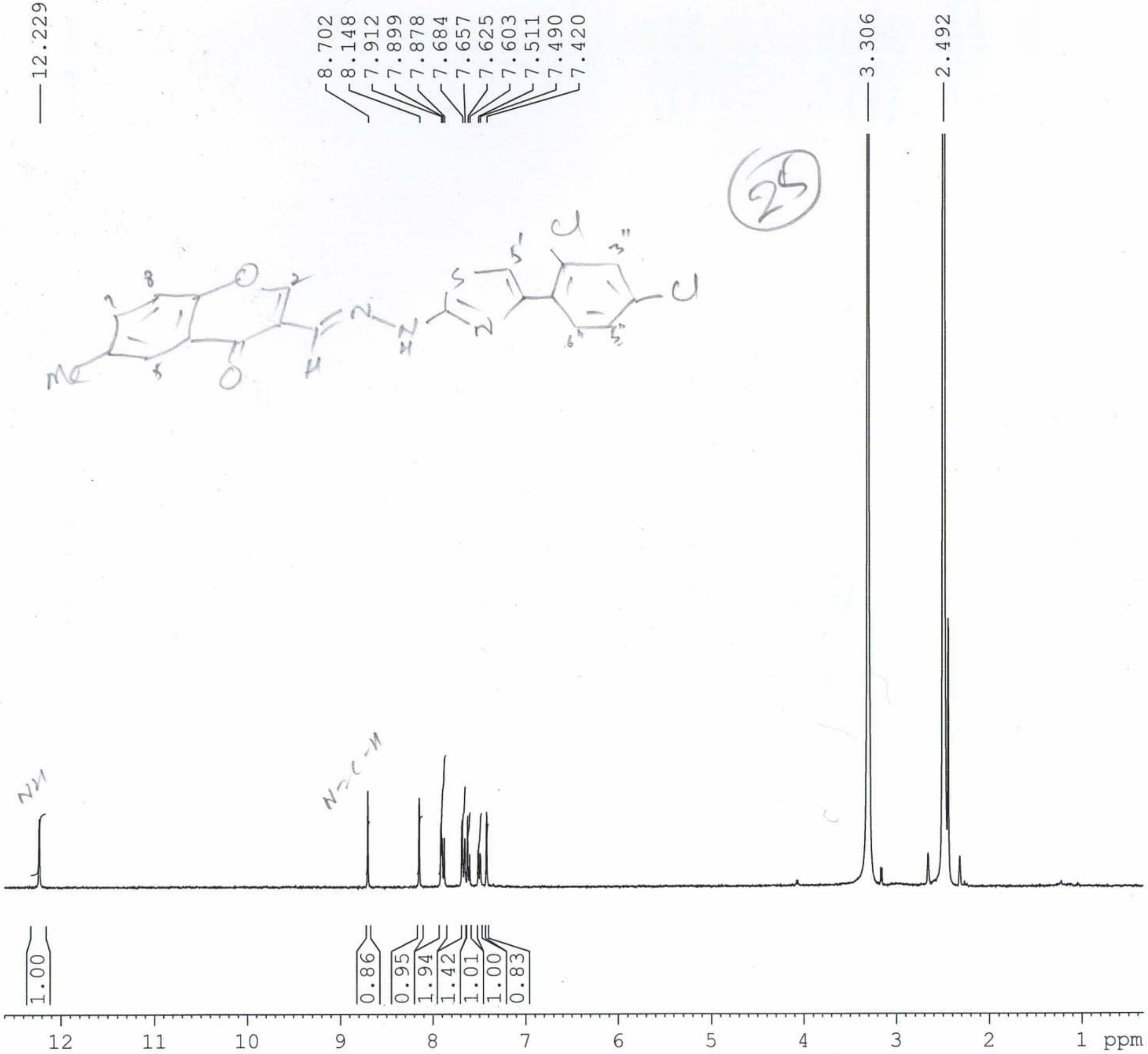

Compound 22

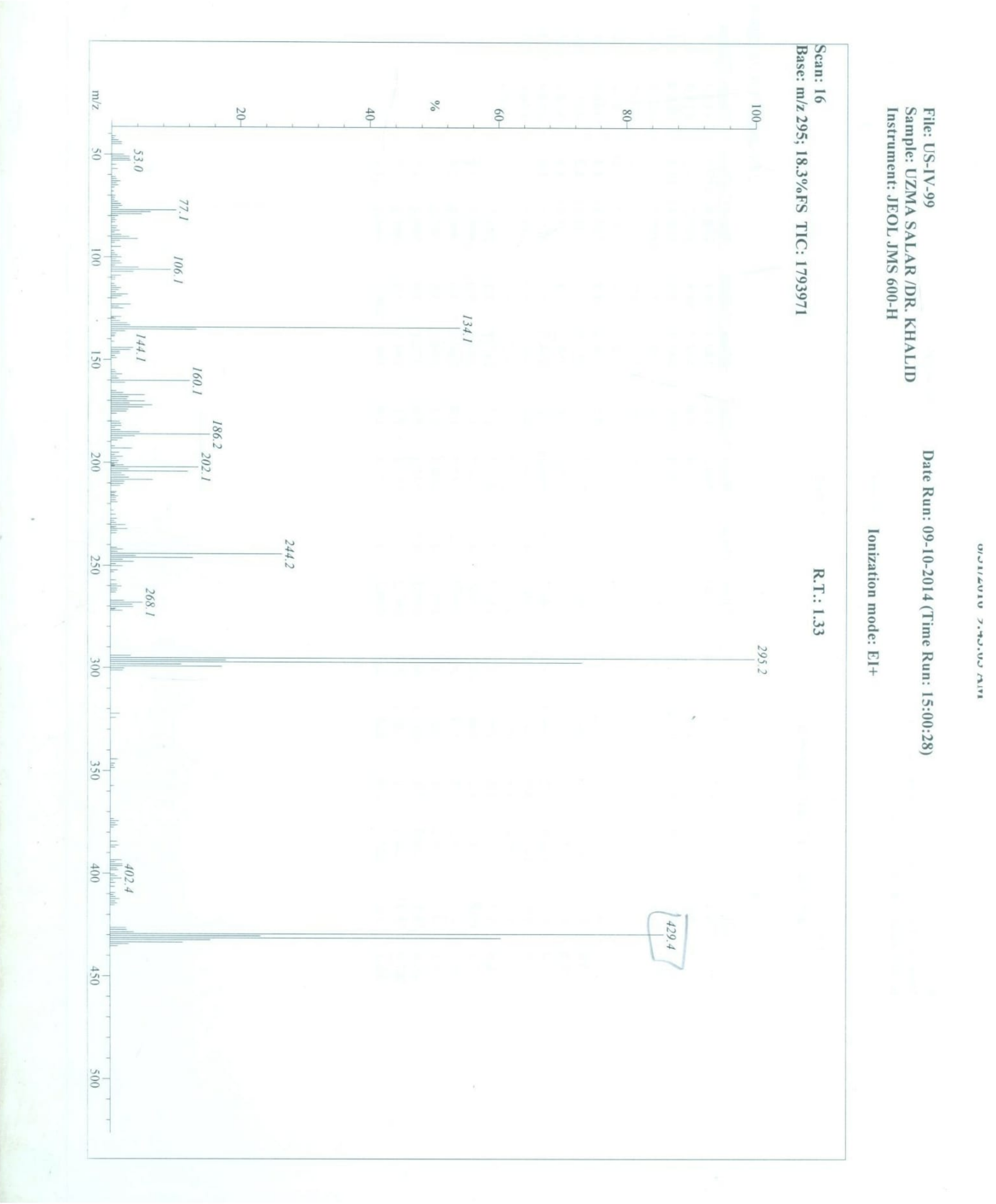
File: US-IV-99
Sample: UZMA SALAR /DR. KHALID
Instrument: JEOL JMS 600-H
Date Run: 09-10-2014 (Time Run: 15:00:28)
Ionization mode: EI+
R.T.: 1.33
Scan: 16
Base: m/z 295; 18.3%FS TIC: 1793971
%
m/z
53.0
77.1
106.1
134.1
144.1
160.1
186.2
202.1
244.2
268.1
295.2
402.4
429.4

UZMA/DR,KHALID/US-IV-99/
ICCBS,U.O.K/

Compound 22

AVANCE 400
LAB NO 117

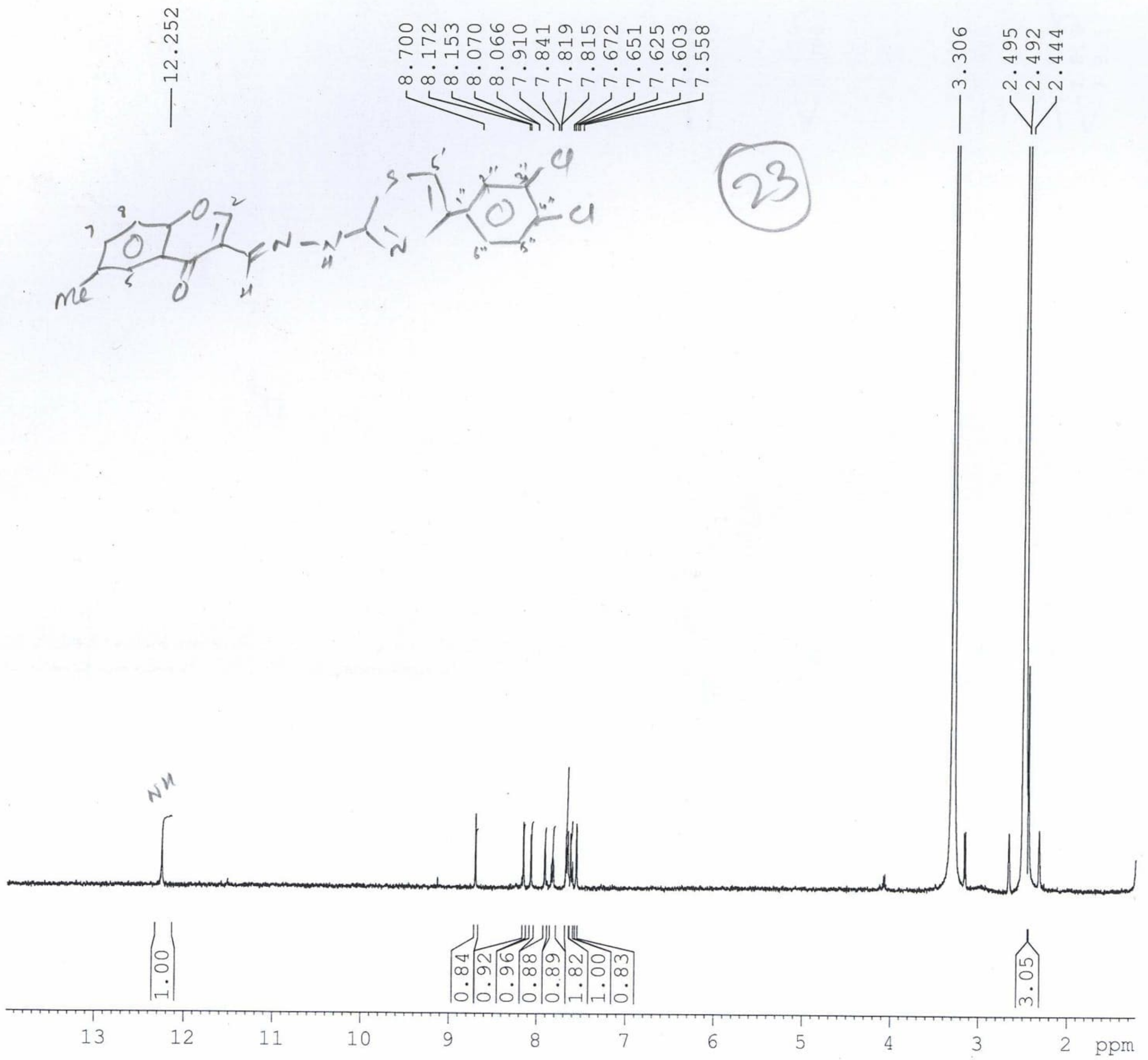

NAME	feb26-15
EXPNO	7
PROCNO	1
Date_	20150226
Time	9.11
INSTRUM	spect
PROBHD	5 mm DUL 13C-1
PULPROG	zg30
TD	32768
SOLVENT	DMSO
NS	64
DS	0
SWH	8012.820 Hz
FIDRES	0.244532 Hz
AQ	2.0447731 sec
RG	362
DW	62.400 usec
DE	6.50 usec
TE	300.0 K
D1	2.00000000 sec
TD0	1

======== CHANNEL f1 ========

NUC1	1H
P1	10.20 usec
PL1	0.00 dB
SFO1	400.1332010 MHz
SI	16384
SF	400.1300064 MHz
WDW	EM
SSB	0
LB	0.30 Hz
GB	0
PC	1.00

ICCBS-HEJ[LAB-104]
9/10/2014

File: US-IV-92
Sample: UZMA SALAR /DR. KHALID
Instrument: JEOL JMS 600-H
Inlet: My Inlet

Date Run: 09-10-2014 (Time Run: 16:32:18)

Ionization mode: EI+

Compound 23

Scan: 13
Base: m/z 261; 78.4%FS TIC: 5092039

R.T.: 1.07

#Ions: 331

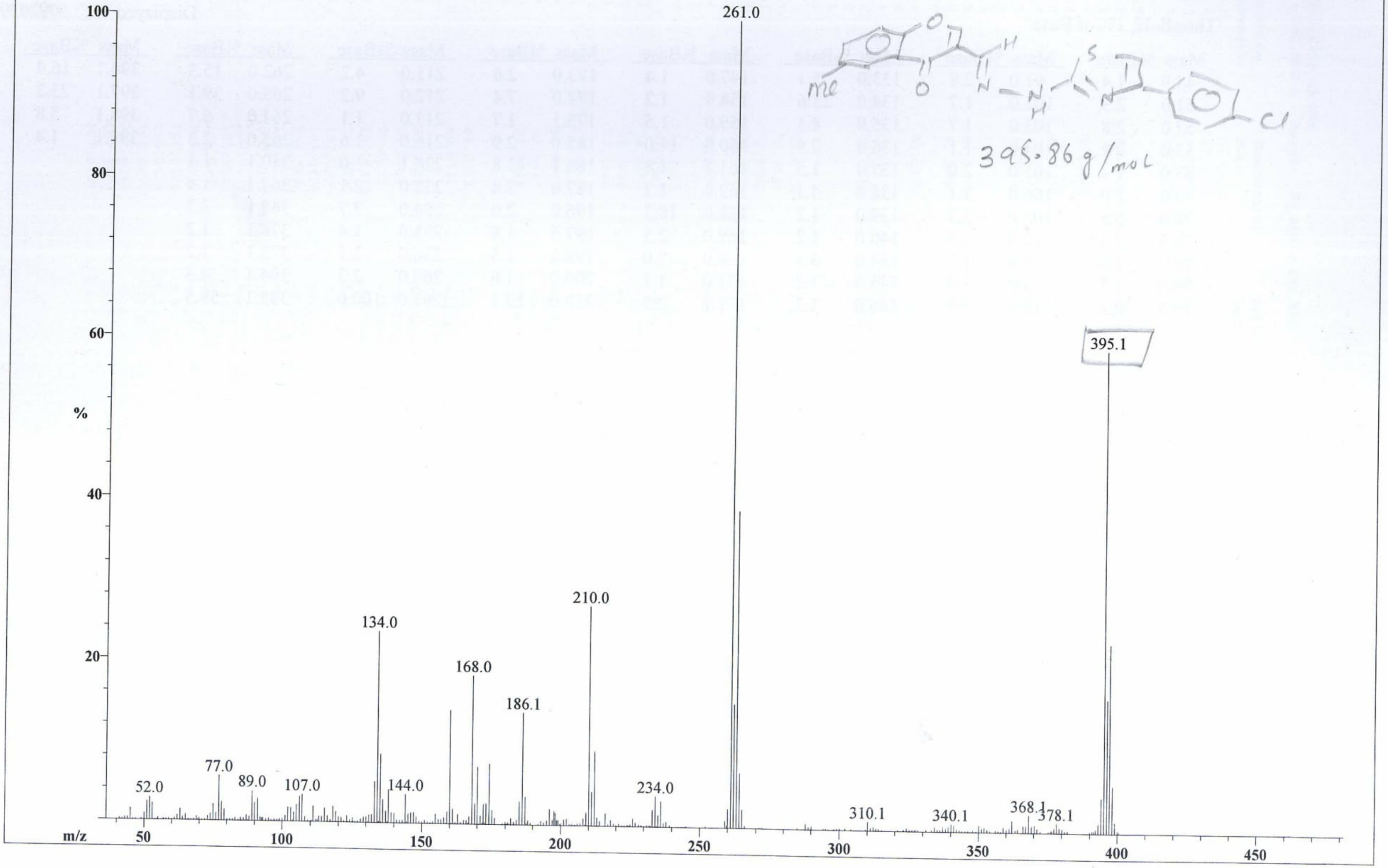

UZMA/DR,KHALID/US-IV-92/
ICCBS,U.O.K/

Compound 23

AVANCE 400
LAB NO 117

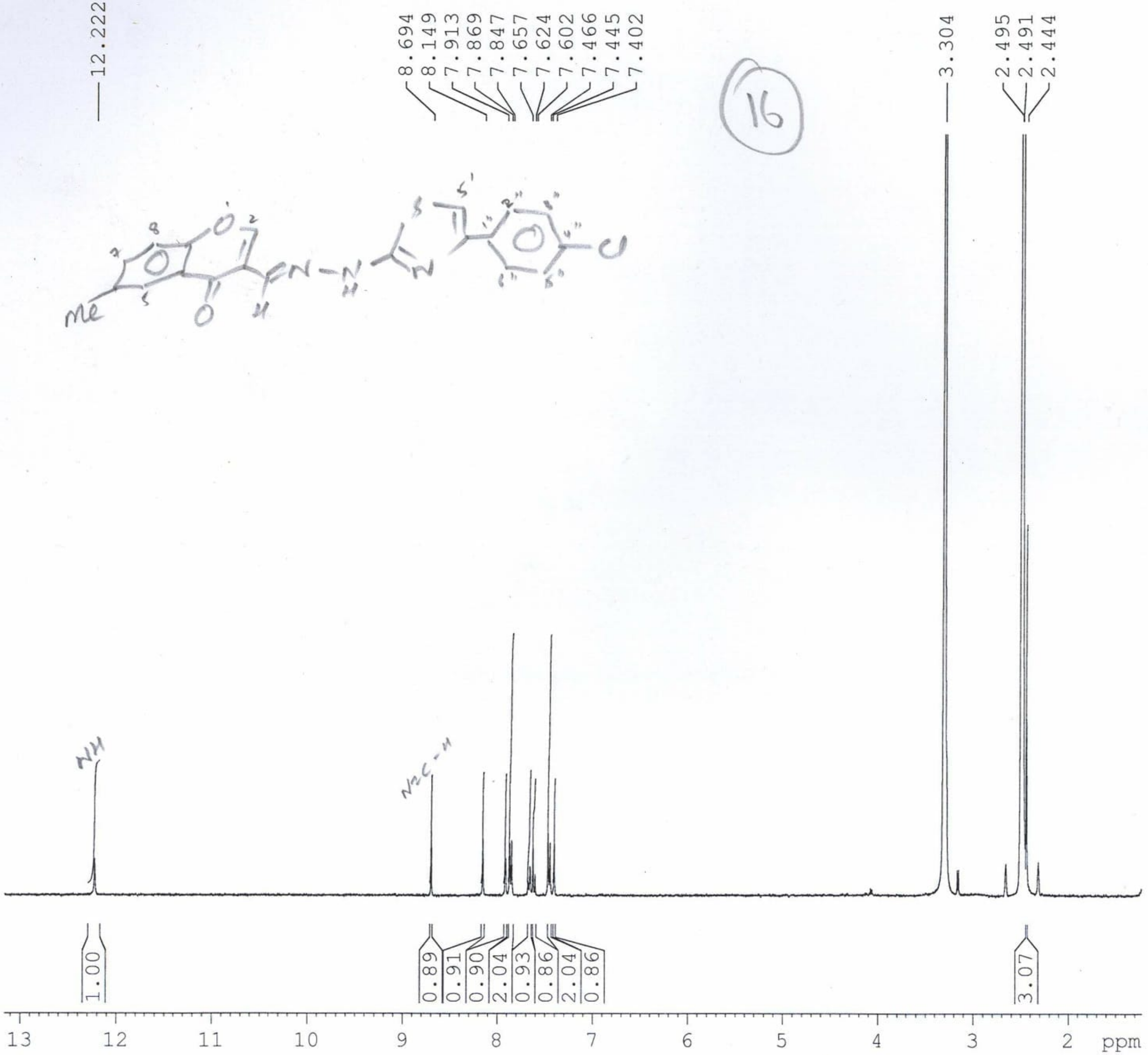

NAME	feb26-15
EXPNO	6
PROCNO	1
Date_	20150226
Time	8.56
INSTRUM	spect
PROBHD	5 mm DUL 13C-1
PULPROG	zg30
TD	32768
SOLVENT	DMSO
NS	64
DS	0
SWH	8012.820 Hz
FIDRES	0.244532 Hz
AQ	2.0447731 sec
RG	362
DW	62.400 usec
DE	6.50 usec
TE	300.0 K
D1	2.00000000 sec
TD0	1

======== CHANNEL f1 ========

NUC1	1H
P1	10.20 usec
PL1	0.00 dB
SFO1	400.1332010 MHz
SI	16384
SF	400.1300064 MHz
WDW	EM
SSB	0
LB	0.30 Hz
GB	0
PC	1.00

File: US-IV-100
Sample: UZMA SALAR /DR. KHALID
Instrument: JEOL JMS 600-H
Inlet: My Inlet

Date Run: 09-10-2014 (Time Run: 16:19:25)

Compound 24

Ionization mode: EI+

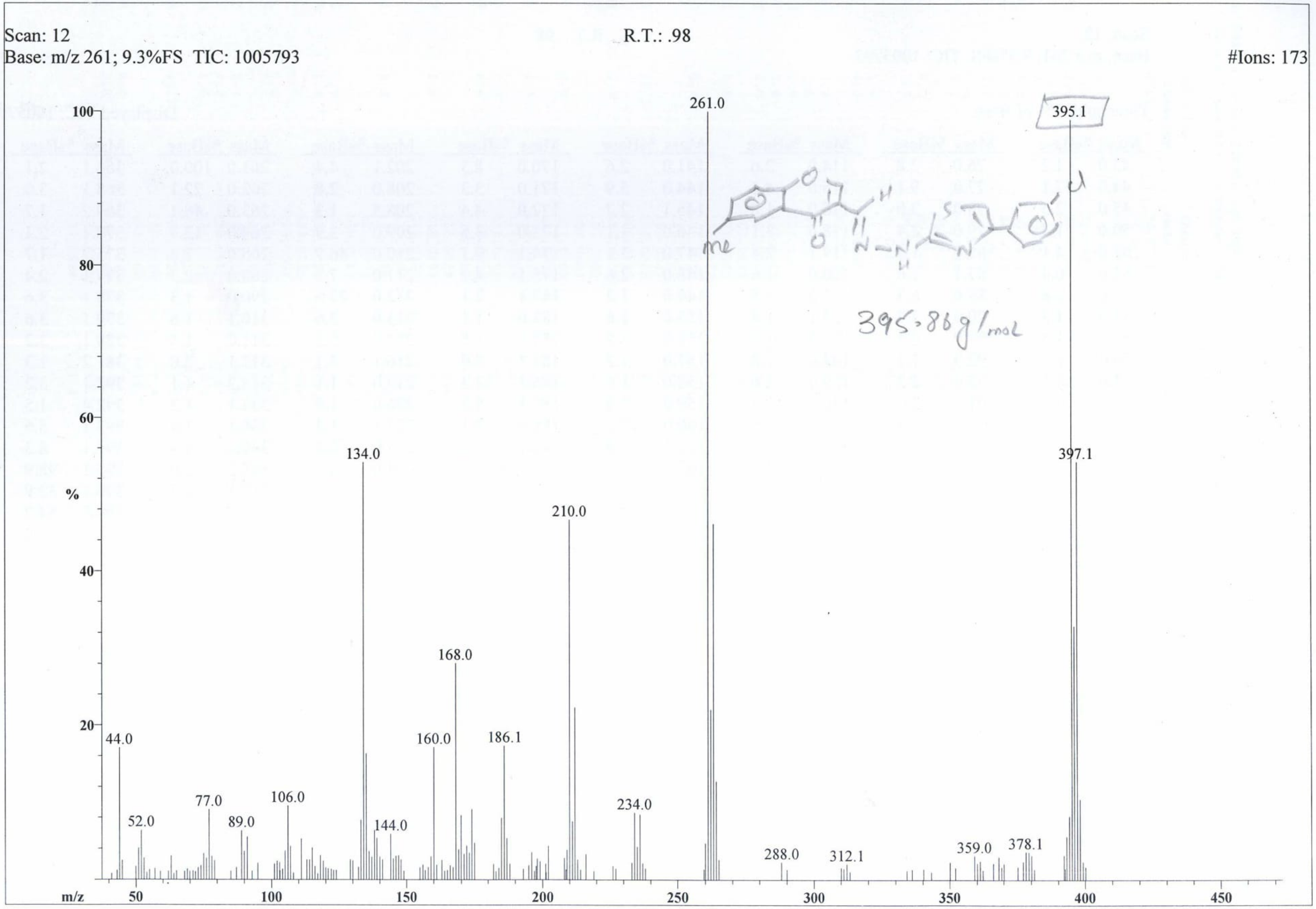

UZMA/DR,KHALID/US-IV-100/
ICCBS,U.O.K/

Compound 24

AVANCE 400
LAB NO 117

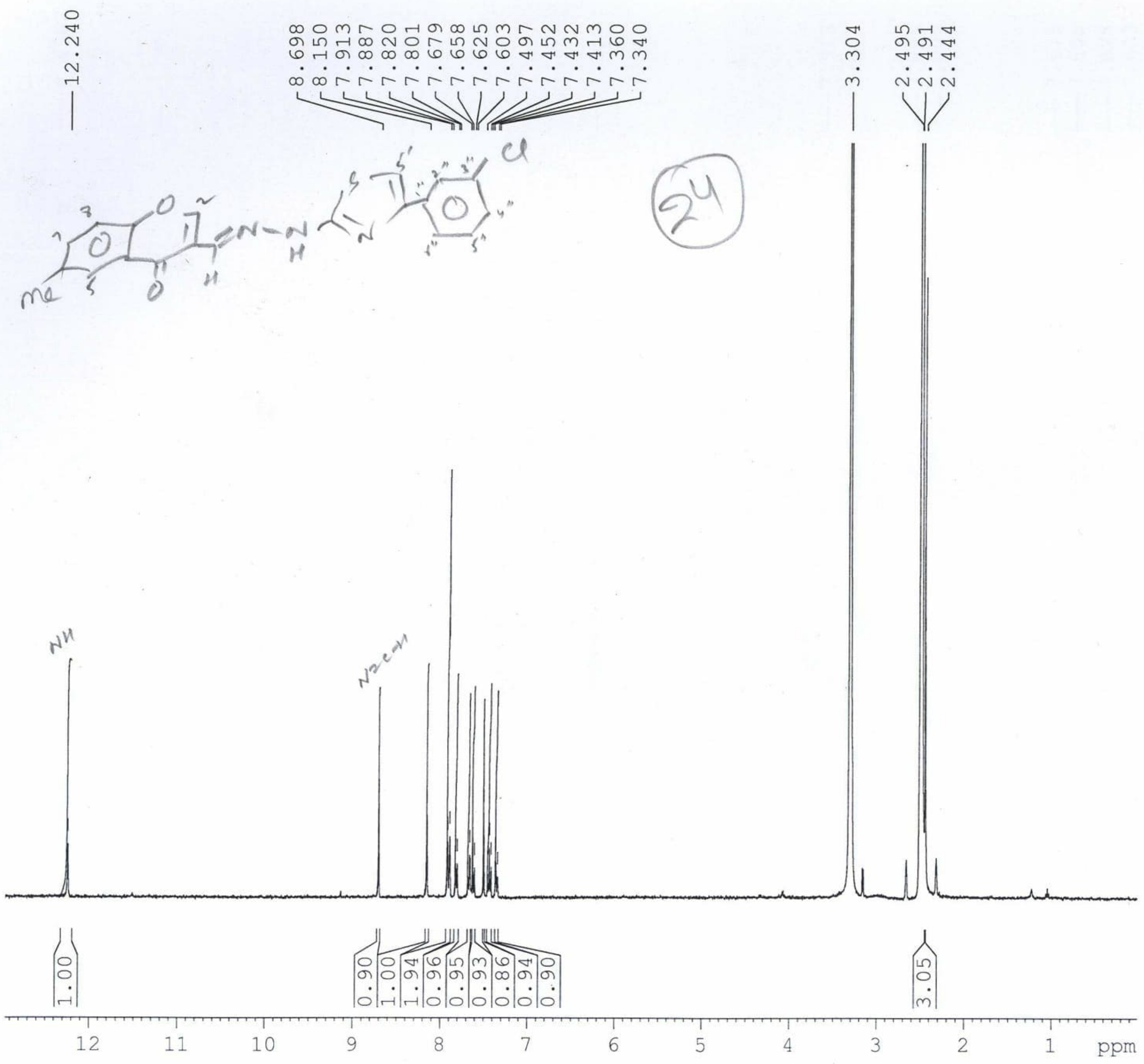

NAME	feb26-15
EXPNO	9
PROCNO	1
Date_	20150226
Time	9.38
INSTRUM	spect
PROBHD	5 mm DUL 13C-1
PULPROG	zg30
TD	32768
SOLVENT	DMSO
NS	64
DS	0
SWH	8012.820 Hz
FIDRES	0.244532 Hz
AQ	2.0447731 sec
RG	362
DW	62.400 usec
DE	6.50 usec
TE	300.0 K
D1	2.00000000 sec
TD0	1

======== CHANNEL f1 ========

NUC1	1H
P1	10.20 usec
PL1	0.00 dB
SFO1	400.1332010 MHz
SI	16384
SF	400.1300064 MHz
WDW	EM
SSB	0
LB	0.30 Hz
GB	0
PC	1.00

File: US-IV-~~96~~ 95
Sample: UZMA SALAR /DR. KHALID
Instrument: JEOL JMS 600-H
Inlet: My Inlet

Date Run: 09-10-2014 (Time Run: 16:07:38)

Ionization mode: EI+

Compound 25

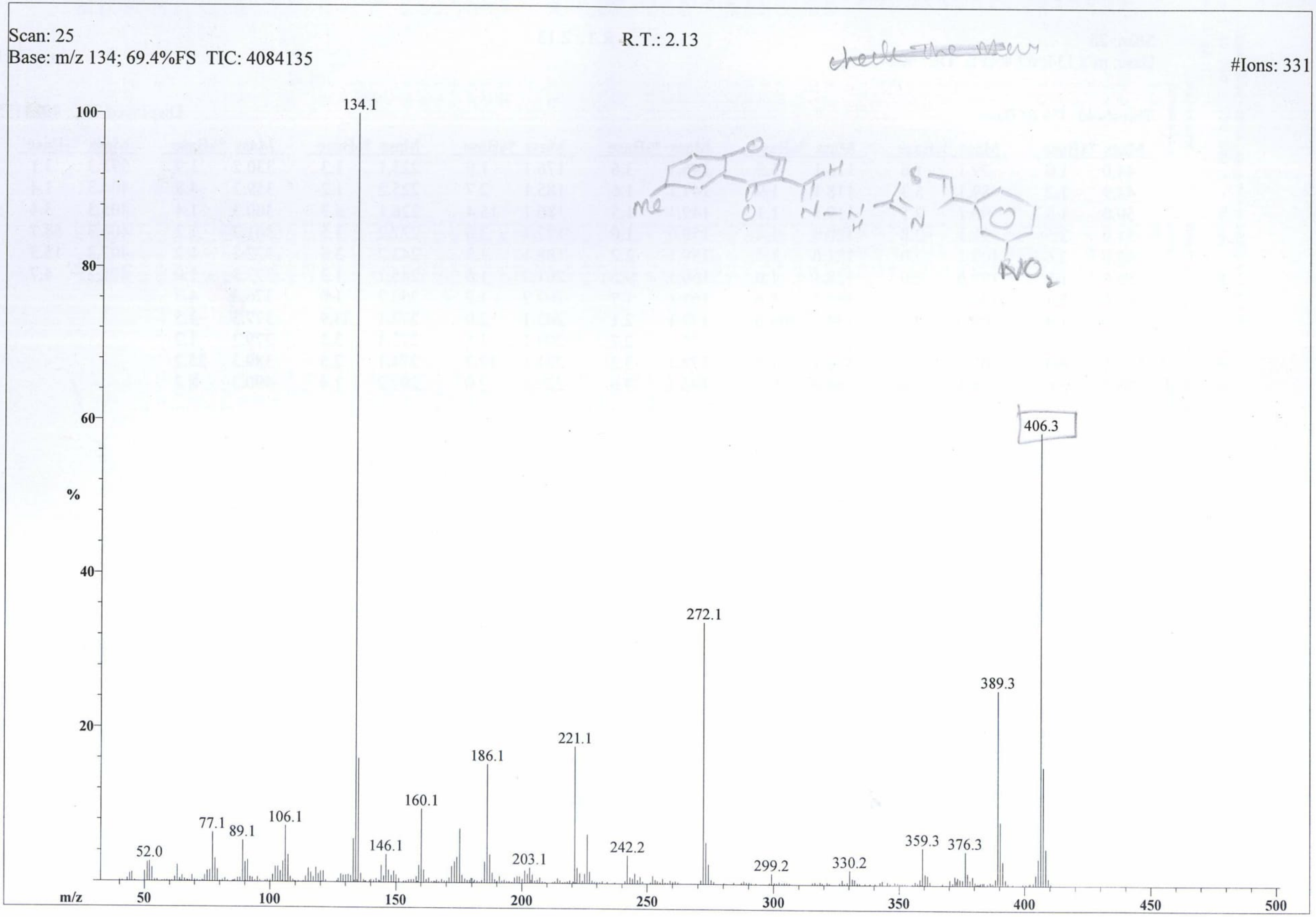

UZMA/DR,KHALID/US-IV-95/
ICCBS,U.O.K/

Compound 25

AVANCE 400
LAB NO 117

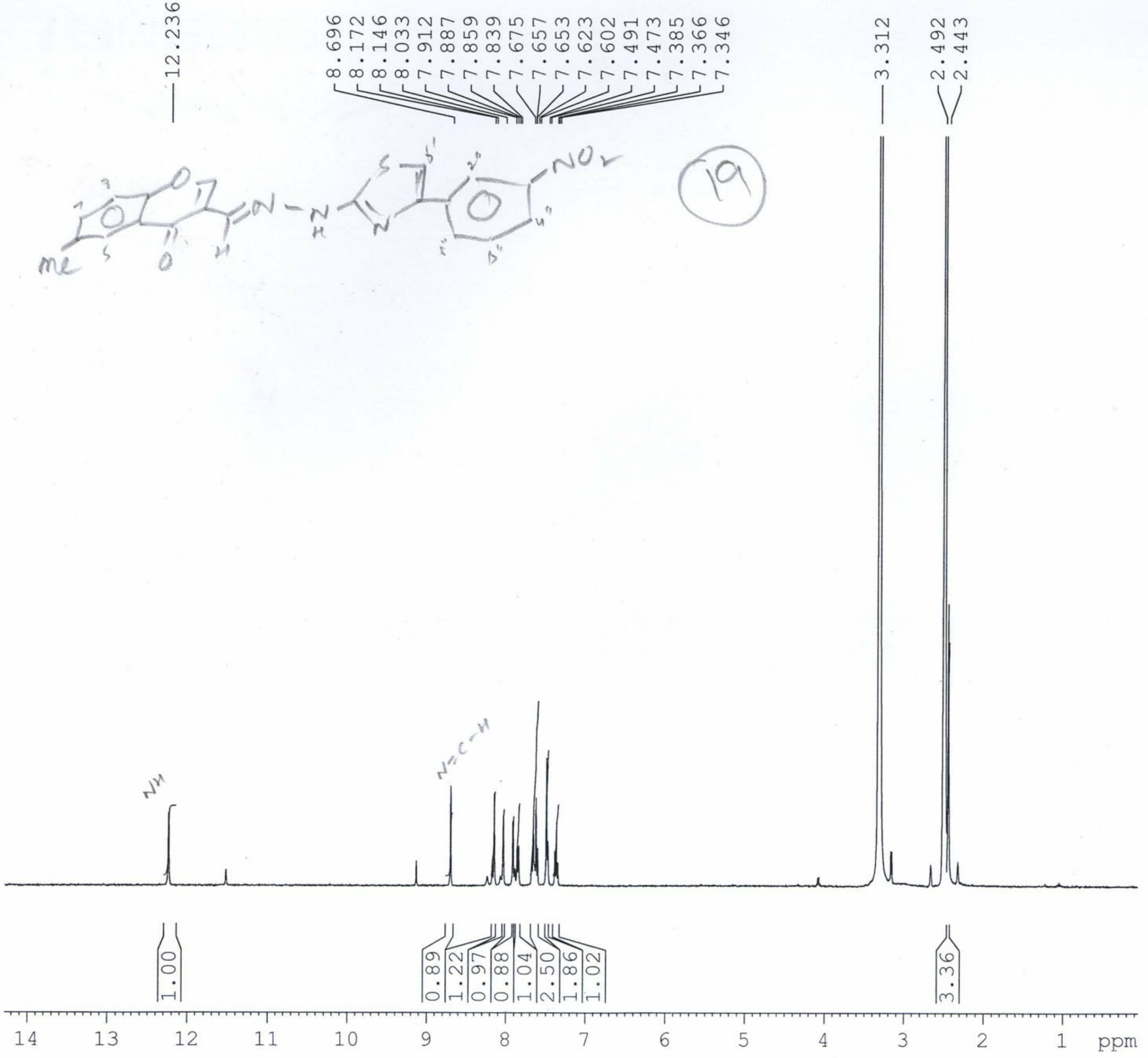


```
NAME          feb02-15
EXPNO                6
PROCNO               1
Date_         20150202
Time             10.42
INSTRUM          spect
PROBHD   5 mm DUL 13C-1
PULPROG           zg30
TD               32768
SOLVENT           DMSO
NS                  64
DS                   0
SWH          8012.820 Hz
FIDRES       0.244532 Hz
AQ          2.0447731 sec
RG                 362
DW              62.400 usec
DE                6.50 usec
TE               300.0 K
D1          2.00000000 sec
TD0                  1

======== CHANNEL f1 ========
NUC1                1H
P1               10.20 usec
PL1               0.00 dB
SFO1       400.1332010 MHz
SI               16384
SF         400.1300064 MHz
WDW                 EM
SSB                  0
LB                0.30 Hz
GB                   0
PC                1.00
```

File: US-IV-94
Sample: UZMA SALAR /DR. KHALID
Instrument: JEOL JMS 600-H
Inlet: My Inlet

Date Run: 09-11-2014 (Time Run: 08:58:33)

Ionization mode: EI+

Compound 26

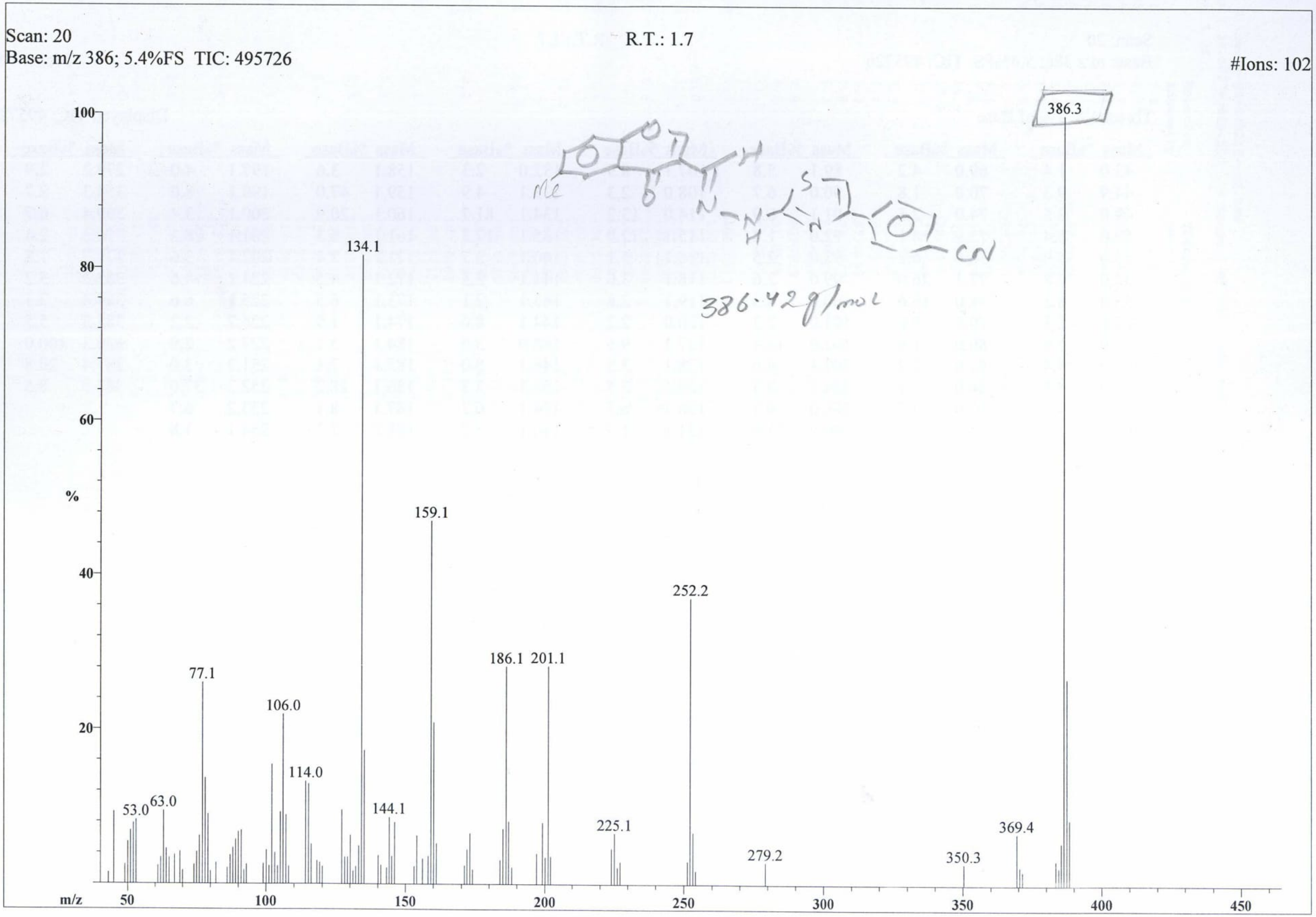

UZMA/DR,KHALID/US-IV-94/
ICCBS,U.O.K/

Compound 26

AVANCE 400
LAB NO 117

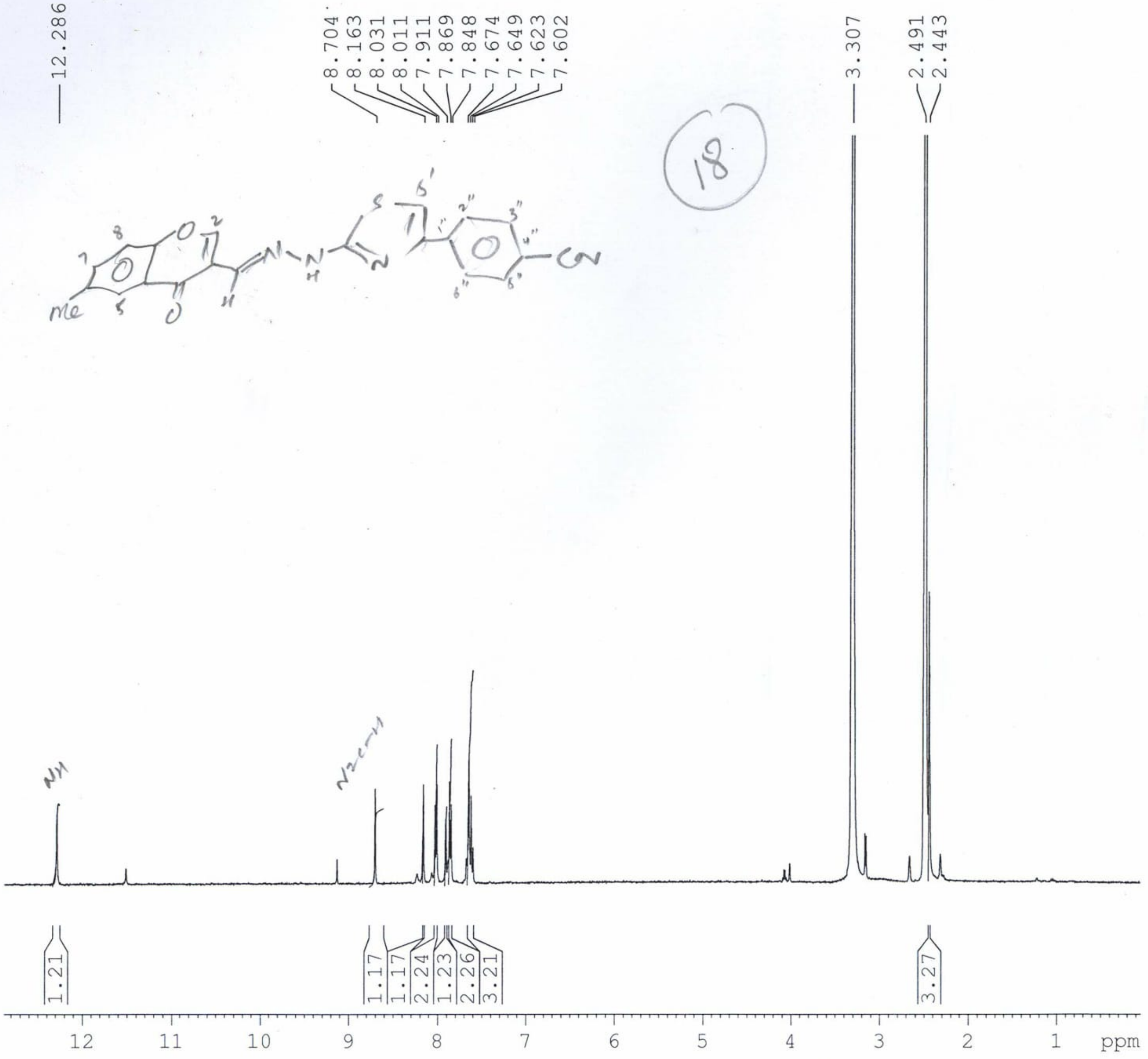

NAME	jan26-15
EXPNO	9
PROCNO	1
Date_	20150126
Time	10.35
INSTRUM	spect
PROBHD	5 mm DUL 13C-1
PULPROG	zg30
TD	32768
SOLVENT	DMSO
NS	64
DS	0
SWH	8012.820 Hz
FIDRES	0.244532 Hz
AQ	2.0447731 sec
RG	362
DW	62.400 usec
DE	6.50 usec
TE	300.0 K
D1	1.50000000 sec
TD0	1

======== CHANNEL f1 ========

NUC1	1H
P1	10.20 usec
PL1	0.00 dB
SFO1	400.1332010 MHz
SI	16384
SF	400.1300064 MHz
WDW	EM
SSB	0
LB	0.30 Hz
GB	0
PC	1.00

File: US-IV-93 **Date Run: 09-10-2014 (Time Run: 15:12:04)** Compound 27
Sample: UZMA SALAR /DR. KHALID
Instrument: JEOL JMS 600-H
Inlet: My Inlet **Ionization mode: EI+**

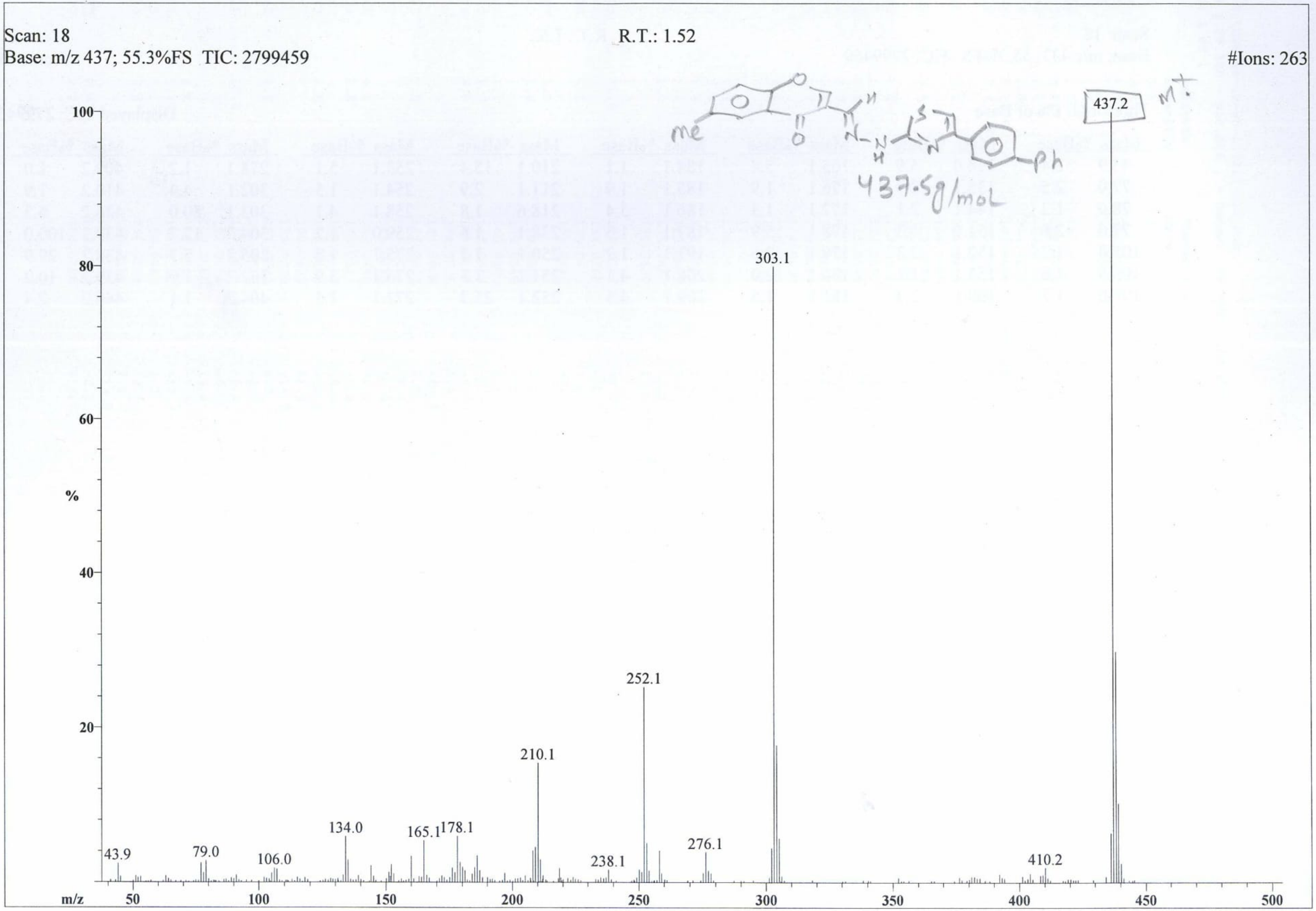

UZMA/DR,KHALID/US-IV-93/
ICCBS,U.O.K/

Compound 27

AVANCE 400
LAB NO 117

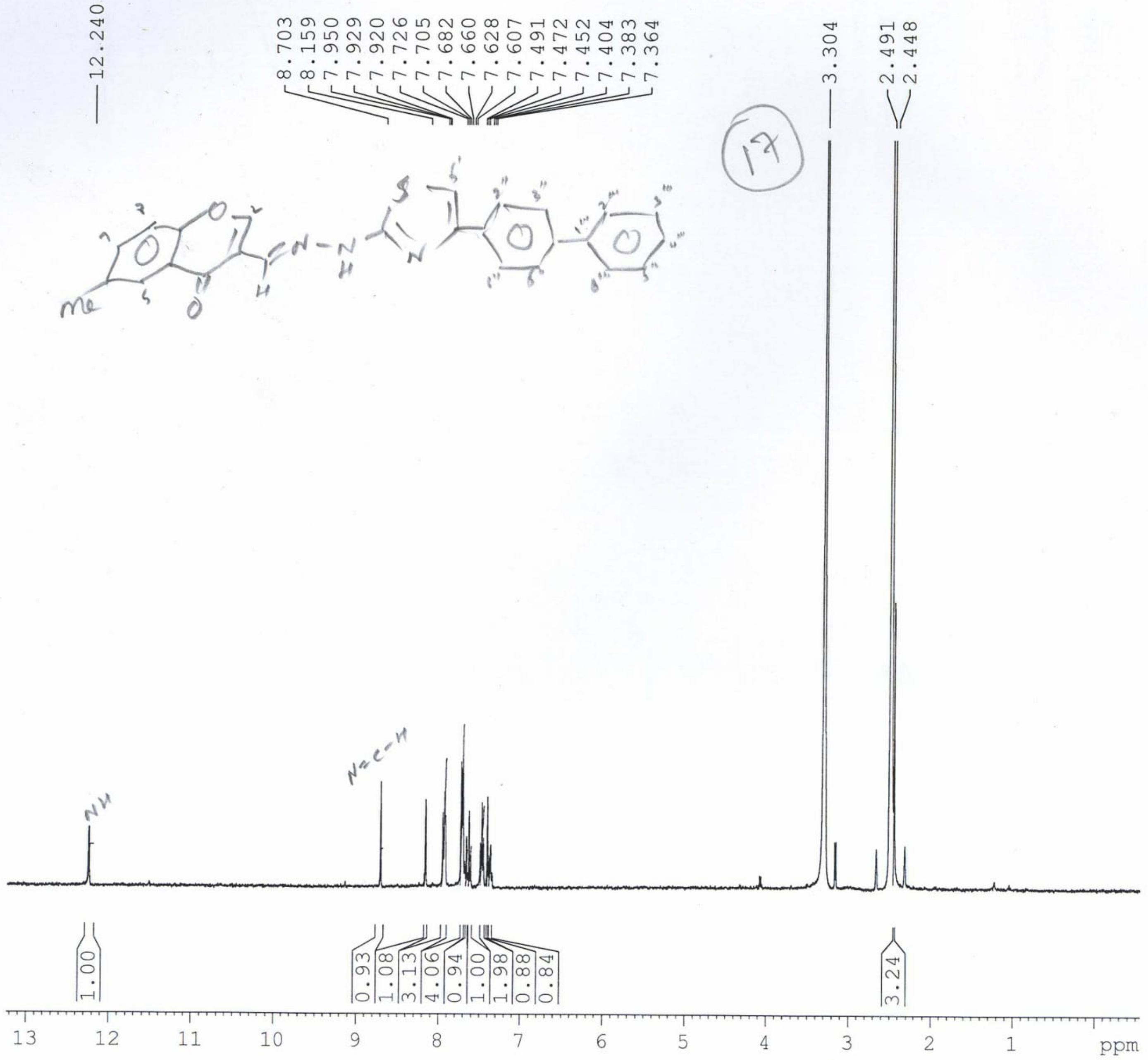

NAME	feb26-15	
EXPNO	10	
PROCNO	1	
Date_	20150226	
Time	9.48	
INSTRUM	spect	
PROBHD	5 mm DUL 13C-1	
PULPROG	zg30	
TD	32768	
SOLVENT	DMSO	
NS	64	
DS	0	
SWH	8012.820	Hz
FIDRES	0.244532	Hz
AQ	2.0447731	sec
RG	362	
DW	62.400	usec
DE	6.50	usec
TE	300.0	K
D1	2.00000000	sec
TD0	1	

======== CHANNEL f1 ========

NUC1	1H	
P1	10.20	usec
PL1	0.00	dB
SFO1	400.1332010	MHz
SI	16384	
SF	400.1300064	MHz
WDW	EM	
SSB	0	
LB	0.30	Hz
GB	0	
PC	1.00	
